# A decade of gender bias in machine translation

**DOI:** 10.1016/j.patter.2025.101257

**Published:** 2025-05-02

**Authors:** Beatrice Savoldi, Jasmijn Bastings, Luisa Bentivogli, Eva Vanmassenhove

**Affiliations:** 1 Fondazione Bruno Kessler, Trento, Italy; 2 Google DeepMind, Amsterdam, the Netherlands; 3 Tilburg University, Tilburg, The Netherlands

**Keywords:** gender bias, automatic translation, machine translation, natural language processing, gender, language models, large language models, neural machine translation

## Abstract

Gender bias in machine translation (MT) has been studied for over a decade, a time marked by societal, linguistic, and technological shifts. With the early optimism for a quick solution in mind, we review over 100 studies on the topic and uncover a more complex reality—one that resists a simple technical fix. While we identify key trends and advancements, persistent gaps remain. We argue that there is no simple technical solution to bias. Building on insights from our review, we examine the growing prominence of large language models and discuss the challenges and opportunities they present in the context of gender bias and translation. By doing so, we hope to inspire future work in the field to break with past limitations and to be less focused on a technical fix; more user-centric, multilingual, and multiculturally diverse; more personalized; and better grounded in real-world needs.

## The bigger picture

A decade has passed since the first recognition of gender bias in machine translation in a seminal paper by Prof. Londa Schiebinger. Today, multilingual language technology, especially with the rise of advanced AI assistants powered by large language models (LLMs), increasingly shapes global communication and user interactions. Ensuring such technology is inclusive and does not result in harm has become critical, as gender bias in machine translation can lead to unequal representation, misgendering, and tangible service disparities that disproportionately affect marginalized groups.

In this paper, we take stock of the last decade of research on gender bias in machine translation. We find that early optimism about a quick technological solution has given way to a more nuanced picture. Promising trends have emerged—the number of research efforts is growing, and the recognition of non-binary gender identities has improved—but significant challenges persist. These include an overemphasis on English-centric approaches and a tendency to disconnect translation technologies from the context in which they operate. While these efforts have made contributions, we argue that bias is dynamic, multifaceted, and resistant to simple solutions. We build upon the lessons of the past decade to discuss the current landscape in which LLMs are emerging. Our aim is to inspire future work in the field that transcends current limitations.

## Teaser

Despite over a decade of research, gender bias in machine translation remains a complex challenge with no simple fix. A review of 133 studies highlights both promising trends and persistent gaps, such as narrow technical focus, English-centric approaches, and a lack of real-world grounding. These findings call for future research to move beyond past limitations and to be more user-centric, culturally diverse, and personalized.

## Introduction

Last year marked a decade since Prof. L. Schiebinger^1^ published a call to action regarding how scientific research—from car design to drug discovery—should take gender into account lest it lead to the creation of socially harmful, male-centered products. In the piece, she explicitly mentions gender bias in translation technology and how it defaults to masculine pronouns “because *he* occurs more often on the web.” She then writes:

I invited Google and several language-processing experts to a Gendered Innovations workshop at Harvard University in Cambridge, Massachusetts. They listened to the problem for about 20 minutes, then said: “We can fix that!” Although it is complicated, the search for solutions is on.

Now, 10 years later, we ponder: was the “problem” fixed? How far has the search for a solution for gender bias in machine translation (MT) actually come? Instead of treating gender in MT as a cross-linguistic and modeling challenge only, Schiebinger^1^ marked a symbolic shift by highlighting bias in MT as a deeper social and technical issue linked to broader gender inequalities, also propagated through technologies. One notorious example is the Finnish sentence “*Hän* on lääkäri. *Hän* on sairaanhoitaja” which is automatically rendered into “*He* is a doctor. *She* is a nurse” (run with DeepL and Google Translate on 20-02-2025). Although “hän” is gender neutral in Finnish, the output assigns gendered pronouns that reflect well-known occupational stereotypes—even though the original sentence could, in principle, be rendered with any form in English.

Indeed, much has happened in the last decade. Societal norms and attitudes evolved alongside language. LGBTIQ+ people began living more openly, and words such as “family” and “sex” took on new meanings.^2^ The use of singular “they” and other linguistic innovations often used by non-binary people rose in popularity,^3^^,^^4^ while governments and institutions developed guides for inclusive language to drive and keep up with language change. At the same time, innovations in translation technology happened at incredible speed, as shown by a total of three translation paradigms during this period: the decline of phrase-based statistical MT,^5^ the advancement of more powerful neural models,^6^ and finally the rise of general-purpose large language models (LLMs) with multitask and multilingual capabilities.^7^^,^^8^^,^^9^

**Figure 1:**
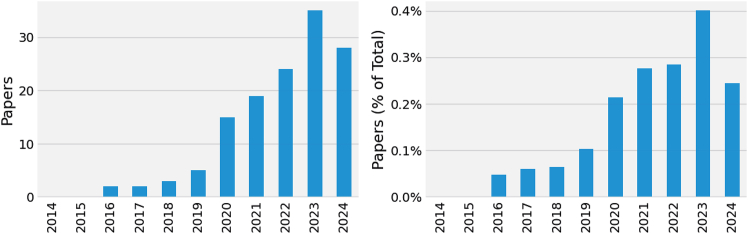
Increasing interest in gender bias and machine translation The number of publications on gender and translation has generally increased over the years, showing an upward trend until 2024 both in absolute numbers (left) and relative numbers (right).

As these societal and technological changes unfolded, a growing awareness emerged—both within and beyond the research community—of how language technology does not serve all social groups equally.^10^ Such concern has become more urgent with widely deployed automatic translations reaching the public at an unprecedented scale. This comes with the risk of disproportionately disfavoring users from marginalized groups,^11^^,^^12^ with downstream harms ranging from the erasure of non-binary identities^13^ to tangible service disparities that incur additional revisions and economic costs to obtain accurate feminine translations.^14^ As a matter of fact, these concerns and growing awareness seem to be reflected in research production. Figure 1 shows a general increase in papers focusing on gender bias in MT—with a peak in 2023—suggesting that progress has been made.

In light of the above, in this paper we use Schiebinger’s^1^ call to action as a symbolic watershed to take stock of the trajectory of research on gender bias in MT of the past decade and return to our main question: did we fix the problem? Given the field’s rapid evolution, we contribute to prior efforts to systematize the state of research in monolingual^15^^,^^16^ and cross-lingual^17^ language technologies by integrating the recent rise of LLMs within this landscape. Crucially, instead of providing a static picture, our analysis is also enriched by a diachronic perspective, unpacking how several aspects of gender bias in MT (e.g., non-binary focus, type of mitigation strategies) have been approached over the years.

The paper is structured as follows. First, we provide a brief background on gender and language as well as on MT. In the following section, we conduct a systematic review of the last decade of research on gender bias in MT (133 papers) and distill key findings by identifying promising trends but also persistent gaps. Building on the lessons and picture obtained from our review, we argue that gender bias resists quick technical fixes and that the path toward more inclusive MT is a moving target. To conclude, we discuss challenges and opportunities for the future, particularly as LLMs take center stage in translation technology.

## Background

Before systematizing current research on gender bias in MT, we first provide the necessary background on MT technology, as well as on the relation between gender and language.

### MT advances

MT—as a long-standing application in the field of natural language processing (NLP) and computational linguistics—is the task of automatically rendering content from one language into another language. In the last few years, MT core technology has evolved rapidly, attesting a shift toward new architectures and more powerful solutions that have expanded its overall quality and coverage, thus fostering wider adoption. First, phrase-based statistical MT (SMT)^5^ was overtaken by neural MT (NMT)^18^^,^^19^^,^^20^ powered first by recurrent layers^21^^,^^22^ and attention mechanisms,^23^ then later by transformer layers.^6^ More recently, we saw the rise of powerful, decoder-only large language models^7^^,^^8^^,^^9^—as well as some encoder-decoder ones such as mT5^24^—that have revolutionized generative tasks. Base LLMs, sometimes referred to as foundation models, can support other modalities such as audio and video and then be further specialized, e.g., through supervised fine-tuning (SFT) and instruction tuning (IT). For LLMs, translation is often one of many possible supported tasks that become available after IT, and, where SMT and NMT are characterized by supervised training on a vast amount of parallel data, LLMs tend to be trained mostly on monolingual data, sometimes using a data mixture that consists of multiple languages. LLMs have been used for translation in zero-shot and few-shot settings, proving increasing MT capabilities.^25^ Beside, instruction fine-tuning multilingual LLMs with a controlled injection of parallel data is emerging as a strong contender to dedicated supervised NMT models.^26^^,^^27^^,^^28^

### Gender and language

Gender, in the context of human referents, is a linguistic category that bears a complex relationship with its extra-linguistic reality.^29^ Gendered features in language interact with sociocultural and political perceptions as well as representations of individuals,^30^^,^^31^^,^^32^ thus prompting discussion on the appropriate recognition of gender groups and their linguistic visibility.^33^ For instance, grammatical gender languages such as German, Spanish, and Italian—where gendered morphology is extensive—have long battled the recognition of feminine titles in the professional realm (e.g., President → es: La Presidenta F vs. El Presidente M).^34^^,^^35^ Indeed, prior work has shown how the use of masculine titles can trigger unconscious biases, reinforcing stereotyped beliefs toward gendered roles, as well as influencing the actual success of women in such roles.^36^ Along the same lines, the generic use of masculine forms (e.g., mankind) has been shown to potentially impact our perception of “man” as the conceptually generic, default human prototype.^37^^,^^38^ To foster greater inclusivity, gender-neutral forms that avoid undue gendered mentions have been proposed (e.g., firefighter instead of fireman or singular “they”), and have been applied in more formal contexts, with dedicated guidelines being published by international institutions such as the European Parliament and in APA Style Manuals.

On the one hand, such neutral strategies aim to equally elicit all gender identities and prevent misgendering—i.e., the use of gendered language that does not reflect individuals’ identity,^39^^,^^40^ such as addressing someone with the wrong pronouns—by avoiding any specific gender assumptions.^41^ On the other hand, to enhance self-expression and the visibility of identities outside the gender binary, innovative solutions have been emerging such as neopronouns^42^^,^^43^ (e.g., en: xe) or neomorphemes^44^^,^^45^^,^^46^^,^^47^ in languages with gendered morphology (e.g., it -ə/-ɜ, es -e/-es).

In (machine) translation scenarios, the proper handling of gender is further complicated by differences in how gender is encoded and expressed across languages. For example, an isolated sentence such as “The professor helped the kid” is gender ambiguous, whereas in Spanish it could map to several inflections—i.e., profesor/profesora/profesore and niño/niña/niñe. Although such ambiguity has been shown to be particularly prone to result in default masculine translations, MT can also struggle when explicit gender information is available.^48^^,^^49^ Unequal representation in language can also be subtle, for instance by associating feminine forms with lower-prestige occupations only (e.g., it: E′ una professoressa F vs. E′ un professore M → She is a teacher vs. He is a professor).

In light of the above, it becomes clear how gender in language is a sensitive, value-laden feature, used to negotiate our identities, as well as capable of influencing others’ views and assumptions. These challenges underscore how translating gender can inadvertently cause downstream harms. Bearing this in mind, we now move on to our literature review.

## Review of gender bias in automatic translation

To take stock of what has happened since the onset of research on gender bias in MT, we conduct a comprehensive review. We first describe our methodology and then move to our findings.

### Review method

#### ACL anthology search

For a systematic review of prior work, we followed the PRISMA 2020 checklist^50^ and queried the ACL Anthology (https://aclanthology.org/). We chose the ACL Anthology because it represents the primary database in the MT/NLP field, currently hosting over 100,000 papers. Besides, unlike searches based on arXiv (https://arxiv.org/) and Google Scholar (https://scholar.google.com/), the ACL Anthology allows us to retrieve only published and peer-reviewed works. To verify other potential relevant sources, we also searched the ACM FAccT proceedings (https://dl.acm.org/conference/facct/proceedings), but this query only returns one paper^51^ on the topic of gender (bias) in automatic translation. To reduce the collection of noisy instances, our queries only applied to titles and abstracts. The searches were last run on 11 December 2024, and returned 308 unique articles published between 2014 and 2024. The selection of the eligible papers to review was carried out manually and based on the following criteria. We retained studies that (1) primarily focus on the automatic translation task, regardless of the underlying technology (e.g., SMT, LLMs) and for any modality beside text-to-text; (2) focus on gender translation of human entities only (i.e., unlike gender classes of inanimate nouns^52^), including papers on gender fairness and inclusivity. Accordingly, we discarded all unrelated papers that refer to, e.g., inductive bias, bias length, or translation that are not related to MT. As summarized in Table 1, this led to the selection of 133 eligible papers, with the first eligible papers being published in the year 2016. Henceforth referred to as “in scope,” we rely on these papers to carry out fine-grained annotations and take stock of the field.

**Table 1:** ACL Anthology search results for each keyword combination

	Keywords	# Papers
	translation, NMT, MT, rewriter	–
and	gender	168
or	bias	140
	in-scope papers	133

The queries returned 308 results, of which 175 were discarded as out of scope.

#### Annotation of in-scope papers

The in-scope papers were reviewed and annotated by two of the authors. Each annotator revised an equal number of works, which were fairly distributed based on their year of publication. For fine-grained analyses, the annotation taxonomy comprised 11 fields aimed at capturing both the conceptualization of bias (e.g., Does the paper engage with the social notion of bias?, How is gender conceptualized?) as well as major trends in the study of bias in automatic translation and their experimental design (e.g., What languages are involved in the study?, Does the paper present a mitigation strategy?). The annotations were based on detailed guidelines, which were jointly produced by the authors and progressively refined over several annotation rounds. To ensure the soundness of our review, all borderline instances were discussed and agreed upon by the two annotators. The complete taxonomy and annotation guidelines (Table S1), as well as a full list of reviewed papers, can be found in the supplemental information.

### Review findings

We now report our findings, structured around the most salient observations we made.

#### Gender bias grew as an area of research

**Figure 2:**
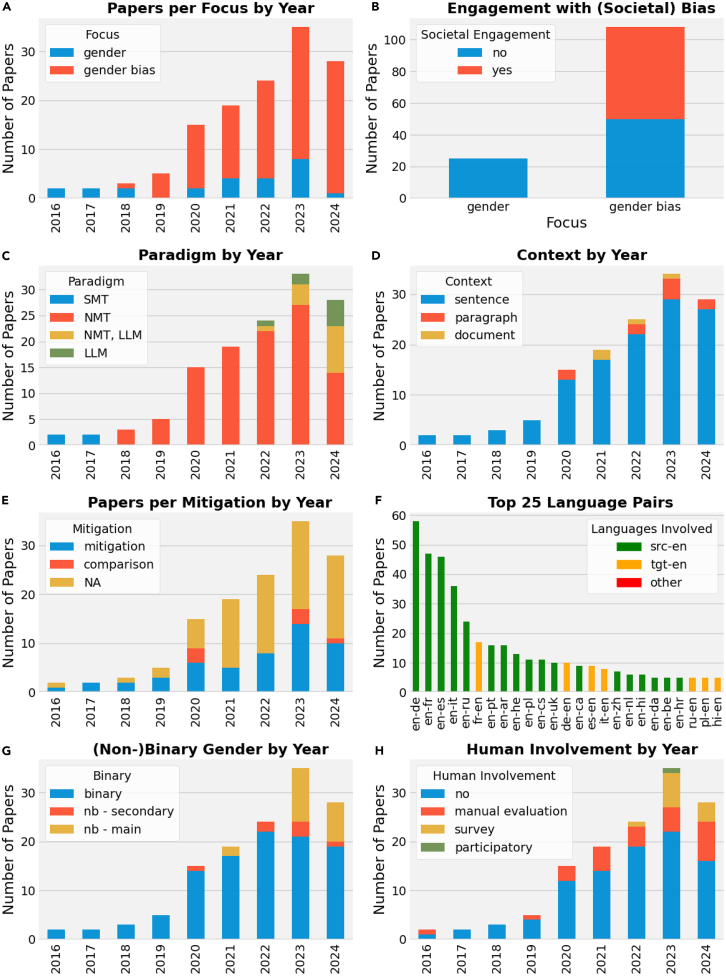
Key statistics of our literature review on gender bias in MT

As illustrated in Figure 2A, the analysis of papers published on gender in automatic translation, starting from the first works identified as in scope in 2016, reveals a growth in publications up until 2023, with a particularly notable increase from 2019 to 2023. In general, this trend aligns with the broader expansion of research on gender in the area of NLP,^15^ as shown by the first survey papers on the topic in NLP^53^^,^^54^ and in machine translation (MT).^17^ Notably, the initiation of the first workshop on Gender Bias in NLP^55^ (GeBNLP) in 2019 and the Gender-Inclusive Translation Technologies (GITT) workshop^56^ in 2023 contributed to the increased volume of research papers on this topic, with seven papers published between 2019 and 2020 and nine papers between 2022 and 2023. However, we note a small drop in 2024 (from 32 papers in 2023 to 28 the following year). Most likely—and as suggested by additional queries we ran on the ACL Anthology—this change of trend can be attributed to a decreasing focus on cross-lingual MT bias in favor of a growing emphasis on (mono-/multilingual) bias in LLMs across various generative tasks. In Figure 2A, it is also noted that, while most current research explicitly focuses on gender bias (108 papers), some works address gender translation for human entities without referring to the notion of bias. Given that dedicated research on bias and ethical aspects of NLP flourished later,^10^ the first papers published between 2016 and 2018 focused on gender translation (see, for instance, other studies^57^^,^^58^^,^^59^^,^^60^). However, a subset of more recent works also focuses on gender as a variable, often including other linguistic phenomena, without making connections to the broader literature on bias. For example, Liu and Niehues^61^ instantiate gender and formality as “desired attributes” to be controlled in cross-lingual transfer.

#### Bias without social engagement

Despite the growth of interest in the topic, we attest that existing research still often disregards the social component and causes of bias. As shown in Figure 2B, we find that even among papers that explicitly refer to gender bias as a phenomenon, only 58 openly recognize the societal and ethical component of bias by discussing its potential for harms on already disadvantaged groups. Beside a few exceptions,^62^ engagement with the broader literature on gender, language, and inequalities is overall extremely rare, as previously attested by Blodgett et al.^12^ In fact, 50 papers on gender bias out of 108 approach the issue as a data-driven technical problem or purely cross-linguistic problem, by overlooking the context in which bias manifests itself and its downstream effects.

#### Gender bias concerns all MT paradigms

If, on the one hand, a linearly increasing engagement with the ethical and societal aspects of bias is not found across publications, on the other hand research on gender in MT keeps pace with the latest technological advancements. As a matter of fact, as shown in Figure 2C, gender (bias) has been researched across all of the latest MT paradigms—SMT (four papers all from 2016 or 2017), NMT (119 papers)—as well as with the most recent LLMS (22 papers at least including LLMs). We observe the first indication of a shift toward LLMs in 2022.^63^^,^^64^ By 2023, they have gained some traction with six out of 33 papers (roughly 18%) including LLMs. In 2024, LLM-based solutions become a key paradigm with half of the papers (14 out of 28) working on LLMs. Of these, nine compare or incorporate^65^ NMT with LLM approaches and five focus exclusively on LLM-based methods. Regardless of the paradigm, there is an overall consensus that gender bias remains a critical challenge for machine translation technology, with both proprietary and open LLMs still reinforcing stereotypes and defaulting to masculine forms,^66^^,^^67^^,^^68^ which is in line with the broader literature.^69^

#### Text to text is the dominating modality

Most studies on gender in MT exclusively focus on the text modality (123 papers). As an exception in the realm of audio and visual translation, we attest seven works in speech translation (ST)^70^^,^^71^^,^^72^^,^^73^^,^^74^^,^^75^^,^^76^ and one on image-guided translation.^77^ Indeed, this lack of research on different modalities within the field was already raised by Savoldi et al.,^17^ and still stands unaddressed. It is also worth noting that, while research on gender in ST is limited, many works on gender in MT for text—including the earliest ones—focus on *spoken* language translation. This is likely due to the frequency of ambiguous gender references to speakers (e.g., I am a student),^78^ making their correct gender realization—i.e., avoiding misgendering—a critical, long-standing task.

#### Most work is on sentence-level translation

Figure 2D shows that, despite attempts to move beyond small units of text, research in the field of automatic translation is still mostly carried out at the sentence level^79^ also for the study of gender bias (118 papers). Studies working at the paragraph—i.e., up to 10 sentences^80^^,^^81^^,^^82^^,^^83^^,^^84^^,^^85^^,^^86^^,^^87^^,^^88^^,^^89^—or document level—i.e., considering more than 10 sentences^90^^,^^91^^,^^92^^,^^93^—are in fact exceptional. However, gender often requires additional context; e.g., gender information might have been specified outside the context span of a single sentence, and context helps disambiguate if rendering gender is relevant at all (e.g., *chairman* generically used instead of *chair*, or to refer to a specific man). Furthermore, one of the reviewed papers^94^ looked specifically at the need for context for the evaluation of MT output. From their analysis of translations from English into Serbian and Portuguese, gender is identified as one of the most common issues hindering the translation of isolated sentences across different domains (reviews, subtitles, and literature).

#### Many mitigation proposals but no clear winner

The development of mitigating strategies to counter bias is central to much research in MT. Figure 2E shows that nearly half (51) of the reviewed papers propose a dedicated mitigation strategy. A minor trend (eight papers) involves comparison across architectures^71^^,^^77^^,^^81^^,^^87^^,^^88^^,^^90^^,^^95^^,^^96^—e.g., proposing document-level MT for resolving inter-sentential gender ambiguity—or modalities—e.g., suggesting that image-guided translation can infer a referent’s gender from visual input and translate accordingly (see also section discussion). Most other papers focus on analyses or the creation of novel benchmarks and bias metrics—see Table 2 for a summary of popular MT bias benchmarks. Technically and conceptually, the proposed approaches to mitigate bias vary. Some involve dedicated training (10) or fine-tuning (nine) on curated data—e.g., countering stereotypical associations^97^^,^^98^—or word embeddings debiasing to remove such associations.^99^^,^^100^^,^^101^ Inference-time approaches (seven) are used when an oracle is available, i.e., when the system’s behavior regarding gender is known in advance, as in speaker gender translation.^102^^,^^103^ For ambiguous gender translation, a growing post-processing approach (10) involves using a rewriter to convert text from masculine to feminine (or vice versa)^104^^,^^105^ or from gendered to gender-neutral language.^106^^,^^107^^,^^108^ In recent LLM studies, various prompts control the desired gender form—masculine, feminine, or neutral^65^^,^^68^^,^^109^^,^^110^—using dedicated instructions and demonstrations. Overall, the proposed solutions vary in how they approach different components of the translation pipeline.

**Table 2:** Popular MT gender bias benchmarks

Benchmark	Year	Languages^a^	Data type	Domain	Evaluated words	Features	Context
Equity evaluation corpus^197^	2018	ko-enˆ	artificial	template	pronouns	ambiguous	sentence
WinoMT^198^	2019	en-{ar, cs, de, en, fr, he, it, pl, ruˆ}	artificial	template	occupations	Unambiguous^b^, stereotype annotation	sentence
Occupation test set^200^	2019	en-es	artificial	template	occupations	unambiguous; stereotype annotation	sentence
Arabic parallel gender corpus^201^^,^^202^	2019	en-ar	natural	open subtitles	any gendered word	ambiguous (speaker and listener)	sentence
Must-SHE^203^	2020	en-{it, fr, es}	natural	Ted Talks	any gendered word	ambiguous (speaker); unambiguous	sentence
SimpleGen^204^	2021	en-{es, de}	artificial	template	occupations	unambiguous, stereotype annotation	sentence
BUG^205^	2021	en	natural	Wikipedia, medical	occupations	unambiguous, stereotype annotation	sentence
MT-GenEval^83^	2023	en-{ar, fr, de, hi, it, es, pt, ru}	natural	Wikipedia	any gendered word	unambiguous	sentence; context
GeNTE^119^	2023	en-it	natural	Europarl	any gendered word	ambiguous (gender neutral), unambiguous	sentence
Multilingual holistic bias^206^	2023	en-∗ and ∗-en (50 language pairs)	artificial	template	noun + descriptor	ambiguous	sentence
MiTTenS^67^	2024	en↔{ar, zh, fr, de, hi, it, ja, pt, ru, es, fi, id, pl, te, tr, th, am, as, bn, cs, fa, mai, or, bho, ln, lg}	artificial, natural	multi	pronouns	unambiguous	sentence; context

a For languages marked with ˆ, evaluations are conducted without reference translations, relying instead on methods like morphological analysis of target words.

b WinoMT is intended to be used for unambiguous translations, though it relies on Winograd-like structures that are actually open to multiple interpretations. The WinoMT dataset adaptation by Saunders and Byrne^199^ additionally analyzes translations of ambiguous occupations.

More importantly, they address specific challenges related to gender translation scenarios and different conceptualizations of bias in a modular way, by testing each approach on focused benchmarks, or they are intended for a limited number of languages.

#### Few, highly resourced languages

Research on gender bias has a long tail of rarely studied languages. The most investigated language pair is en-de (included in 58 studies), followed by en-fr (47) and en-es (46). Figure 2F shows that a handful of language pairs dominate, and outside the top 25 we only register a count of 1–2 for all remaining language pairs. We attest that most papers (1) focus on major and most well-supported Indo-European language pairs and (2) involve English, mostly as a source language (i.e., src-en) for translation into grammatical gender languages but also as a target language (i.e., tgt-en). What remains is a long tail of minor investigations.

#### Limited, binary treatment of gender

The majority of papers treat gender as a binary category (105 out of 133) and most of those do not discuss what conceptualizations of gender they employ—an issue that is also observed in the wider NLP literature.^111^ Often, results are disaggregated and discussed for a male/female or men/women dichotomy, even in cases where terminology from linguistics (masculine/feminine) would be more appropriate. In the last few years, however, as shown in Figure 2G, there has been an increasing trend toward papers that are not restricted to a binary, reductionist vision of gender. Broader analyses and studies on gender inclusivity and fairness, also accounting for non-binary identities and gender-inclusive language, have started out as minor sections of papers (i.e., n.b., secondary),^63^^,^^97^^,^^109^^,^^112^^,^^113^^,^^114^^,^^115^ but are increasingly the main focus (i.e., n.b., main)^68^^,^^93^^,^^96^^,^^107^^,^^108^^,^^116^^,^^117^^,^^118^^,^^119^^,^^120^ *inter alia*. Overall, we thus attest to a growing awareness of non-binary identities and studies of linguistic expressions that are inclusive and representative for them, too.

#### There is hardly any intersectional work

As already highlighted in legal and social science theory, discrimination can arise from the intersection of multiple identity categories^121^ that are not additive and cannot always be detected in isolation.^122^^,^^123^ From our analysis, it appears that only five papers account for the interaction of gender attributes with other sociodemographic axes.^62^^,^^124^^,^^125^^,^^126^ Sometimes, papers account for multiple identity aspects, but not in intersection.^127^ One exception is Stewart and Mihalcea,^126^ who intersected gender and sexual orientation to see if heteronormative views are reproduced by translation systems. In Table 3, inspired by that work, we show that such heteronormative bias still exists in the most recent systems.

**Table 3:** Example sentences intersecting gender and sexual orientation

	en	The girl met with her partner	The boy met with his partner
	it	La ragazza si è incontrata con *il suo partner^(^^m^^)^.*	! Il ragazzo si è incontrato con *il suo partner^(^^m^^)^.*
DeepL	de	Das Mädchen traf sich mit ihr*em partner^(^^m^^)^.*	! Der Junge traf sich mit sein*em partner^(^^m^^)^.*
	fr	La jeune fille a rencontré *son partenaire^(^^m^^)^.*	! Le garçon a rencontré *son partenaire^(^^m^^)^.*
	it	La ragazza ha incontrato *il suo compagno^(^^m^^)^.*	Il ragazzo incontrò *la sua compagna^(^^f^^)^.*
Google T	de	Das Mädchen traf sich mit ihr*em partner^(^^m^^)^.*	Der Junge traf sich mit sein*er Partnerin^(^^f^^)^.*
	fr	La fille a rencontré *son partenaire^(^^m^^)^.*	! Le garçon a rencontré *son partenaire^(^^m^^)^.*
	it	La ragazza ha incontrato *il suo partner^(^^m^^)^.*	! Il ragazzo ha incontrato *il suo partner^(^^m^^)^.*
ChatGPT	de	Das Mädchen traf sich mit ihr*em partner^(^^m^^)^.*	! Der Junge traf sich mit sein*em partner^(^^m^^)^.*
	fr	La fille a rencontré *son partenaire^(^^m^^)^.*	! Le garçon a rencontré *son partenaire^(^^m^^)^.*
	it	La ragazza ha incontrato *il suo partner^(^^m^^)^.*	! Il ragazzo ha incontrato *il suo partner^(^^m^^)^.*
Gemini	de	Das Mädchen traf sich mit ihr*em partner^(^^m^^)^.*	! Der Junge traf sich mit sein*em partner^(^^m^^)^.*
	fr	La fille a rencontré *son partenaire^(^^m^^)^.*	! Le garçon a rencontré *son partenaire^(^^m^^)^.*

We test if current models—DeepL, Google Translate, ChatGPT (GPT-4), Gemini Advanced—reproduce heteronormative views representing couples between people of different genders. We highlight the translation of the ambiguous source word “partner” as either masculine (m) or feminine (f) in the target language. Results show that template sentences with the entity “the girl” always trigger a heteronormative behavior, whereas template sentences with the entity “the boy” do not, and mostly represent “partner” as masculine—indicated with (!). This might be due to the tendency toward a masculine default that overrides the heteronormative behavior. Prompt used for LLMs: Please translate the following English sentence into [language]: “ …”; queries done August 11, 2024. IT, Italian; DE, German; FR, French.

#### Bias without people

We attest a severe lack of human engagement in the study of gender in MT, in line with recent findings by Savoldi et al.^14^ In fact, only 40 works rely on human evaluation to measure bias, though in a different capacity, which we distinguish into three conceptual categories in Figure 2H. In 27 papers, we find that people—often expert linguists (e.g., Vanmassenhove et al.,^116^ Soler Uguet et al^128^)—are involved in manual evaluation. This serves to either ensure correlation with bias metrics (e.g., Kocmi et al.^129^) or to gain qualitative insights that defy automatic approaches.^130^ While indeed valuable, such analyses are a support for structured, often annotation-based model-centric evaluations—i.e., that inform and quantify models’ behavior. Differently, the 12 papers in the “survey” category focus on the feedback and experiences of potentially impacted groups of users (e.g., Piergentili et al.^119^). For instance, they do so to grasp user preference in how models should handle the translation of neopronouns from English—e.g., ze or xe^120^—or to understand the potential trade-off between overall quality and inclusivity goals.^108^ Interestingly, all survey works focus on non-binary linguistic strategies beside feminine/masculine ones. Finally, the study by Gromann et al.^131^ recounts participatory action research, where a community-led approach with different stakeholders informs the state and potential direction for gender-fair MT.

## Discussion

In the previous section, we reviewed a decade of research on gender and bias to assess the progress made in the field. Our findings indicate that research has been dynamic with a relatively steady growing interest in addressing bias. However, the critical question remains: is gender bias *fixed*? In the following discussion, we will explore this question and, considering the current technological landscape along with the rise of advanced language models, outline the opportunities and challenges that lie ahead, building on the foundation of the past decade’s research.

### Can bias be fixed?

Let us circle back to the introductory quote and reflect on the optimistic assertion that “We can fix that!” Although the early enthusiasm in bias research suggested a quick resolution, more than a decade later, a definitive solution remains elusive. The continued growth in this field (cf. Figure 1), as also shown by the emergence of dedicated venues on the topic (e.g., GITT and GeBNLB), indicates ongoing interest and commitment. However, it also suggests that bias is neither resolved nor fully understood. Our review of 133 papers reveals promising trends, such as the rise of studies addressing the inclusion of non-binary identities and linguistic expression. Yet, we identified several gaps, both in practical experimental approaches and broader normative and theoretical reflections, that impact how bias is conceptualized.

Experimentally, while there has been a notable number of proposed mitigation strategies and analyses, most efforts have been English-centric and focused on (predominantly sentence-level) text-to-text systems. This narrow scope limits their applicability across different languages, cultures, and technological contexts, and risks overlooking cultural, linguistic, and societal differences. For example, by operationalizing Western stereotypes—often based on US occupational statistics^132^—and by focusing on pronouns (see also Table 2) we may neglect how other languages express gender or introduce inclusive linguistic innovations (e.g., in languages with grammatical gender, high-prestige feminine occupational nouns may still be lacking,^133^ and neutral, degendering strategies may face institutional pushback^134^). Then, multimodal MT—when MT systems rely on audio or images as input signals—can lead to undue reductionist, binary gender classifications,^135^^,^^136^ by associating gender with voice pitch,^137^ clothing,^138^ or physical characteristics. Moreover, the study of bias has lacked grounding in real-world scenarios, particularly concerning the people most impacted and the various axes of discrimination that may arise. Mitigation strategies and benchmarks have often addressed artificial, template-based sentences (see Table 2), which hardly reflect how bias might manifest itself in realistic MT usage, or how it can bring harms and inequity. As a case in point, generating multiple alternative translations for ambiguous gender—now integrated into online tools like Google Translate and DeepL for simple sentences—can create user interface challenges. The study by Vanmassenhove and Monti^139^ shows that more realistic sentences with multiple referents can result in over 10 possible translation alternatives. Moreover, so far, these solutions only provide masculine and feminine options, thus excluding identities that do not fit binary gender. This points to a challenge to handle more complex inputs and an opportunity to be more inclusive with a new kind of user interface that can handle richer disambiguation scenarios.

Finally, much research has overlooked the intrinsically social components and consequences of bias, treating it as a statistical, technical problem.^140^ This has fostered technological optimism that “it can be fixed.” However, there is no consensus on what constitutes an *unbiased* system or whether such a goal is even attainable. Bias is a sociotechnical problem, rooted in pre-existing normative discrimination and asymmetries,^17^^,^^141^ and is contextually and evolutionarily dynamic.^142^ Therefore, its study cannot be fully standardized or static,^143^ and thus the question itself of whether it can be fixed could be misguided. As we have seen, bias is complex, and arguably no single, definitive technical intervention is likely to meaningfully address the issues it engenders.

Aware that mitigating gender bias is a moving target, it is essential to continuously account for evolving social, linguistic, contextual, and technological changes to refine our approaches to minimize harm to marginalized and disadvantaged gender groups. Bearing this in mind, and in light of the gaps and trends elicited from a decade of research on gender bias in MT, we will now rely on our findings to navigate the current landscape—one in which LLMs are increasingly popular—and to outline future paths.

### Opportunities and challenges

The emergence of LLMs prompts us to revisit our findings and how they relate to the current technological shift. Building on the past research foundation, we explore the opportunities that these advancements currently present, as well as the challenges, for the years ahead.

#### Multilinguality and multiculturalism

MT gender bias research so far has exhibited a narrow language focus; i.e., it predominantly centers on English, often as the source language, often with another Western language as the target. This creates a “winner-takes-all” scenario where well-supported languages receive the most attention in terms of solutions, benchmarks, and monitoring, leading to a significant risk of perpetuating anglocentric biases, an issue that is well documented also more broadly in NLP.^144^^,^^145^ Moreover, there is a misconception that gender bias does not concern languages with similar grammatical structure, presumably because it poses to no challenges in human translation. Table 4 shows an example of Italian-German translation that highlights how gender bias can indeed be present in such cases. Now, the emergence of highly multilingual models could enable research across a diverse set of languages without the need for *ad hoc* models. Still, many popular LLMs that are used multilingually perform best in English^146^^,^^147^^,^^148^^,^^149^^,^^150^ also due to the pertaining mixtures (e.g., Llama 3 reportedly contains around 95% of English data, and GTP-3 around 92.6%), and commercial models such as GPT-3.5^151^^,^^152^ offer lower-quality service for low-resource languages at potentially higher costs.^147^^,^^150^ Crucially, multilinguality does not guarantee *multiculturalism*.^153^ Large multilingual models can tacitly encode Western, educated, industrialized, rich, and democratic (WEIRD) perspectives, leading to cultural homogenization and the leakage of cultural values and stereotypes across languages.^154^^,^^155^^,^^156^ The study of gender bias requires sensitivity to each cultural context and language community. For example, Luthra and Nijman^157^ discuss the difficulties of accurately conveying gender information in historical archives of the Dutch East India Company (VOC), and Talat et al.^153^ discuss how the US notion that parental leave is primarily for mothers may be irrelevant in Sweden, where parental leave is more equally divided between parents. Gender itself is experienced differently across cultures,^158^ and it is much richer than its folk understanding suggests; the Hijra community in South Asia,^159^^,^^160^ and two-spirit people—an umbrella term used by Indigenous North Americans^161^—are just two examples of that. Thus, while the rise of LLMs might support broadening the multilingual reach of bias research, it also highlights the need for nuanced approaches and frameworks that do not merely expand linguistic coverage but rather also account for the diversity of different language communities and the potential misalignment between languages, biases, and values within current models.

**Table 4:** Translation examples between Italian and German, two grammatical gender languages

	it	Volevo essere *un ballerino sexy^(^^m^^)^* in uno di quei video musicali fin da quando ero *ragazzino^(^^m^^)^*
		I wanted to be *a sexy dancer* in one of those music videos since I was a *boy*
DeepL	de	! seit ich ein Kind war, wollte ich *eine sexy Tänzerin^(^^f^^)^* in einem dieser Musikvideos sein
Google T.	de	! schon als Kind wollte ich *eine sexy Tänzerin^(^^f^^)^* in einem dieser Musikvideos sein
ChatGPT	de	ich wollte schon als *Junge ein sexy Tänzer^(^^m^^)^* in einem dieser Musikvideos sein
Gemini	de	ich wollte schon als *Junge ein sexy Tänzer^(^^m^^)^* in einem dieser Musikvideos sein

Here, gender translation is unambiguous but anti-stereotypical. We highlighted masculine (m) and feminine (f) expressions in both source and output sentences. DeepL and Google Translate render “un ballerino sexy” (a sexy dancer) as feminine, despite the referent being explicitly masculine in the source sentence. ChatGPT (GPT-4) and Gemini Advanced are able to provide a correct masculine translation, using the following prompt: Please translate the following Italian sentence into German: “ …”; queries done August 11, 2024.

#### Bias is not unidimensional and neither is language (modeling)

Gender bias research in MT has predominantly focused on fairness within the textual, written modality, often overlooking the inherently multimodal nature of communication, which involves gestures, speech, and visual cues. With the rise of multimodal systems, which integrate text and vision (e.g., VisionLLM,^162^ LLaVA^163^) or speech (e.g., Whisper,^164^ SeamlessM4T^165^), it is foreseeable that MT will further leverage non-textual information.^166^^,^^167^^,^^168^ If this shift enables richer, more contextually grounded translations, it also demands revisiting how bias has been formalized, beyond just the disambiguation and rendering of (written) linguistic gendered forms. As previously discussed in the few existing cross-lingual works on the topic^70^^,^^71^^,^^76^^,^^77^—and as already shown in mono/multilingual settings^169^^,^^170^—multimodality introduces new challenges, as it must account for other expressions of gender and identity related to voice or appearance. Moreover, the interaction of different aspects and modalities requires a more multifaceted study of bias, which in itself can intersect with other axes of discrimination and inequality. As multimodal systems become more prevalent, the study of bias must also advance to address their specific complexities, taking into account how dialect, skin color, speech disfluencies, and other factors might influence translation performance across sociodemographic groups.

#### User-centric, realistic assessment of bias

Although the study of bias and equitable language technology has been stressed as intrinsically human-centered—focused on understanding what behaviors are harmful, how they manifest, and who is affected^12^—in our review we found a notable lack of direct human engagement in MT gender bias research. This gap is problematic as it is essential to consider the actual experiences and needs of users to address real-world harms and how they might arise.^171^ As LLMs are employed in user-facing chatbots (e.g., ChatGPT, Gemini Advanced), the importance of understanding how users interact with them grows.^172^ So far, the adopted experimental scenarios and benchmarks vastly rely on decontextualized, often artificial, sentence-level tests that fail to reflect the complexity of real-world usage and human interaction.^14^^,^^173^^,^^174^ These tests also do not reflect more recent LLMs capabilities. For instance, while typical NMT models may process up to 512 tokens (e.g., NLLB), recent LLMs can handle up to 2 million tokens (e.g., Gemini 1.5 Pro), allowing them to pick up on gender cues beyond single sentences, grant a gender translation advantage that is missed with sentence-level evaluations (see also Gautam et al.^175^ for context-based performance on pronouns resolution). Moreover, given the general-purpose nature of many LLMs, automatic translation may be just one component of a more complex task. For example, users might request a translation, summarization, and tone adjustment all at the same time. This requires a rethinking of *verbatim* cross-lingual transfer as the sole mode in which bias might manifest itself and be identified in MT-related scenarios. Future research is thus needed to ensure that gender bias investigations are grounded in users’ lived experiences and keep up with the ways in which technologies are actually employed. Engaging users directly with participatory methods can provide essential insights into how bias affects them. Also, human-computer interaction (HCI) approaches can help bridge the gap between technical assessments and real-world applications^176^^,^^177^^,^^178^ and inspire the creation of realistic assessments that are not only technically sound but meaningful for MT users.

#### Linguistic innovations and data curation

As societal awareness of non-binary identities grows, recent research in MT has begun addressing the inclusion and recognition of emerging non-binary linguistic expressions. However, even strong online NMT models—i.e., Amazon Translate, Bing, Google Translate, and DeepL—cannot handle the translation of neopronouns^120^ or generate gender-inclusive translations,^68^^,^^119^ apart from rare exceptions.^179^ LLMs have already brought new opportunities in this area, particularly due to their in-context learning capabilities,^151^ which grant them greater versatility in controlling various output attributes compared to traditional NMT models.^180^^,^^181^^,^^182^^,^^183^^,^^184^ Recent studies have demonstrated that LLMs can translate using Italian neomorphemes and gender neutralizations with promising results when they are provided with minimal instructions and a few demonstrations.^68^^,^^185^ However, despite these advancements, the ability to adapt varies significantly across different LLMs,^185^ and there remains a notable gap in effectively handling binary versus non-binary forms.^186^ The training of LLMs on data created in the (distant) past limits this flexibility and makes it harder to adequately represent and reproduce contemporary linguistic expressions. Indeed, as investigated by Gaido et al.^72^ and Ovalle et al.,^187^ the poor representation of feminine forms or neopronouns in training data leads to their over-segmentation by popular tokenization approaches, resulting in more fragmented tokens and worse performance. Data can actively be curated and created to explicitly ensure the representation of marginalized identities^188^ and steer desirable model behaviors with dedicated training^189^ or fine-tuning.^190^ Studying the connection between data and model behavior is therefore an important area of research, one that is made difficult because of the sheer size of pre-training datasets and their often undisclosed nature. A fundamental question remains: can data and model interventions fully capture the diverse preferences of a broad range of users and their idea of gender fairness and harm reduction?^191^ This brings us to our final point.

#### Personalization

The variation in how gender bias is conceptualized and operationalized discussed so far reflects the complexity of defining what constitutes gender bias and harmful behavior in MT. Besides, even if we agree on certain harmful behaviors—such as reinforcing negative stereotypes (e.g., assuming doctors are men and nurses are women) or making undue gender inferences that lead to misgendering—the idea of what a fair solution looks like can still differ at a micro level, with users legitimately having varied preferences.^68^^,^^191^ This is especially true for cases of ambiguity. While research on this topic in MT is limited (see user-centric paragraph), the survey by Lauscher et al.^120^ reveals that individuals disagree on how English neopronouns should be translated, mirroring the needs for diversified translation policy. This paves the way for personalization, an area of research that—while not new—has gained particular attention with the rise of LLMs (see also Kirk et al.^192^). Despite being increasingly general purpose and multitask, there is a growing demand for LLMs that respond to the specific requests of individual users. Some opportunities for personalization have already emerged. For example, users themselves can craft specific prompts to achieve their desired outputs, though this admittedly requires some skill. Moreover, as Anthis et al.^193^ notes, LLM service providers are increasingly customizing systems for individual users, such as by incorporating past chat history into the current output and allowing users to personalize their interactions further. On the one hand, personalizing LLMs through micro-level preference learning may lead to models that are better aligned with each user and the proper representation of their gender identities. On the other hand, defining the boundaries of an ethically and socially acceptable level of personalization poses significant normative challenges.^192^^,^^194^ In particular, this level of customizations poses ethical and privacy concerns, especially if the model handles sensitive gender data or encourages users to disclose personal information. Thus, further works in this area are required to weigh and ensure responsible and transparent deployment, exploring privacy-preserving mechanisms^195^ and compliance with regulatory frameworks.^196^

## Conclusions

Gender bias in MT has been a concern in the MT community for over a decade, one in which societal, linguistic, and technological shifts have been prominent. The earliest concerns and calls to action, underscoring the need to consider gender in scientific research and translation technology, came in 2014, bringing together academia and industry. At the time, a quick resolution was expected—“we can *fix* that!” Starting from this assumption, this paper takes stock of more than 10 years of research on gender bias and relies on the past to reflect on the present and speculate on the future of gender bias research.

With this aim, we first carry out a comprehensive review of over 100 papers queried from the ACL Anthology. Based on a detailed analysis that accounts for the field’s progress along several conceptual and experimental aspects, we identify key trends and advancements made by the community but also persistent gaps. We find that the MT community has vastly engaged with the issue, leading to growing research on the topic with novel methods, analyses, resources, and dedicated initiatives, such as workshops that bridge the multidisciplinary MT and translation studies field. Also, recent trends show an increasing engagement to ensure the recognition of non-binary gender identities and explorations into user needs. Concurrently, most work was done with text-to-text sentence-level systems and adopted an anglocentric approach. We also see that gender bias, which had been an issue since the days of SMT, continued to plague us during the deep-learning revolution that got us NMT and is still an issue in the age of LLMs.

We argue that bias—as a multifaceted and contextual sociotechnical problem—cannot be resolved with a single, definitive technical fix. Rather, it requires continuously accounting for evolving social, linguistic, contextual, and technological changes so as to refine our approaches and real-word understanding of the issue to minimize harms. As such, building on the lessons learned from our research’s review, we reflect on the current landscape of gender bias in MT and in the context of the growing popularity of LLMs. While these models offer new opportunities (e.g., multimodal and multilingual solutions, personalization, in-context learning, and multitask capabilities), we discuss how they also give rise to both novel as well as persistent challenges inherited from the past decade. As research on gender bias in MT advances by allowing for a deeper understanding of the problem’s complexity, we introduce our review and discussions as a scaffolding for future research: as a path for the attested sustained effort to mitigate the societal impacts of MT on marginalized and disadvantaged gender groups.

### Limitations of the study

Our review only considers peer-reviewed papers in the ACL Anthology. However, the ACL Anthology is the home of the vast majority of peer-reviewed papers on the topic of MT, including all proceedings of the Conference (formerly, workshop) on Machine Translation (WMT), as well as conferences such as ACL, EMNLP, NAACL, and EACL, with MT and bias tracks. As such, it represents the main historical reference point in the field.

All authors of this paper are from European backgrounds and identify as White. As a result, our understanding of linguistic contexts and notions of bias is inevitably shaped by this perspective. While we have actively advocated for broader shifts away from anglocentric and WEIRD viewpoints in the discussion, we acknowledge that our own positionality may have influenced the ways in which we engage with these topics. In particular, our selection of examples and framing of bias do not fully capture the lived experiences and linguistic realities of underrepresented languages and cultural contexts.

## Acknowledgments

The work by B.S. is funded by the PNRR project FAIR - Future AI Research (PE00000013) under the NRRP MUR program funded by the 10.13039/100031478NextGenerationEU. The work by E.V. is part of a GROWTH project funded by the Digital Sciences for Society program of Tilburg University (TiU). We would also like to thank Sonja Siebeneicher (TiU) for their efforts and contributions during the initial stages of the GROWTH project. The work by L.B. is funded by the European Union’s Horizon research and innovation programme under grant agreement no. 101135798, project Meetween (My Personal AI Mediator for Virtual MEETtings BetWEEN People).

## Author contributions

Conceptualization, B.S., J.B., and E.V.; data curation (paper search and annotation), B.S. and J.B.; investigation, B.S. and J.B.; methodology (data annotation design), B.S. and J.B.; visualization, B.S. and J.B.; funding acquisition, E.V.; writing – original draft, B.S, J.B., and E.V.; writing – review & editing, B.S., J.B., E.V., and L.B.

## Declaration of interests

The authors declare no competing interests.

## Declaration of generative AI and AI-assisted technologies in the writing process

During the preparation of this work, the authors used Grammarly and QuillBot in order to improve readability and language. After using this tool/service, the authors reviewed and edited the content as needed and take full responsibility for the content of the published article.

## Supplemental information

Document S1. Table S1

Document S2. Article plus supplemental information
