# Supplementary material for: A decade of gender bias in machine translation
Source: Patterns (N Y). 2025 May 2;6(6):101257. doi: 10.1016/j.patter.2025.101257 (PMC12191736; doi:10.1016/j.patter.2025.101257)
Supplement: Document S1. Table S1 [file mmc1.pdf]

Patterns, Volume *6*

Supplemental information

A decade of gender bias in machine translation

Beatrice Savoldi, Jasmijn Bastings, Luisa Bentivogli, and Eva Vanmassenhove

Supplemental information

Supplemental Table: Papers' Annotation Guidelines

The annotation guidelines are listed in Table S1.

Field	Description
Focus	Is the focus on gender bias?<br>• GENDER: focus on human gender translation, without explicit mention to the notion of bias<br>• GENDER BIAS: focus on human gender translation, with explicit mention to the notion of bias
Binary	How is gender conceptualized?<br>• BINARY: only binary gender, even if only implicitly<br>• NB-SECONDARY: discusses *at least* a third, non-binary category considered in a minor section<br>• NB - MAIN: centers on non-binary gender
Context	How much context is taken into account?<br>• SENTENCE: no additional context beside the sentence-level<br>• PARAGRAPH: from one up to 10 sentences as context<br>• DOCUMENT: more than 10 sentences as context
Language	What are the considered languages or language pairs?<br>• LANGUAGE CODE: indicate the languages or language pairs pairs considered using two or three letter codes, e.g., en-de for English to German. Consider only the languages for which a gender-based metric score (i.e. disaggregated by gender) is provided, and disregard aggregate overall quality translation scores used only as baselines<br>• NA: the label does not apply. The paper is not experimental and does not consider a specified set of languages.
Bias	Does the paper explicitely engage with the societal notion of gender bias?<br>i.e. it recognizes the societal and ethical component of bias, e.g. by discussing its potential for harms on already disadvantaged groups. Use Bentivogli et al. [1] as reference point of minimal engagement.<br>• YES: it engages with the societal notion of gender bias, at least as much as Bentivogli et al. [1]<br>• NO: it does not engage with the societal notion of gender bias at all, or not as much as Bentivogli et al. [1]
Human Involvement	Are people involved in assessing bias?<br>This excludes annotation work for data creation.<br>• NO: no human participant is involved<br>• MANUAL EVALUATION: human participants are involved for (model-centric) manual evaluations or validations, e.g. to correlate human judgments with automatic metrics<br>• SURVEY: human participants are involved to elicit their feedback, experiences and preferences. Can be further distinguished into SUREVY-NOMT if a survey is a available but not MT model is used in the study<br>• PARTICIPATORY: human participants are actively involved in participatory actions and approaches
Modality	Which modality is considered?<br>• TEXT: text-to-text<br>• AUDIO: speech-to-text<br>• VISUAL: image-guided-text-to-text

Mitigation	Is an approach to improve gender translation or a bias mitigation strategy proposed?<br>• YES: an approach to improve gender translation or a bias mitigation strategy is proposed<br>• NO: approach to improve gender translation or a bias mitigation strategy is not proposed (e.g. only analyses)<br>• COMPARISON: a comparison across baseline models for different modalities or architectures (e.g. document-level MT) is proposed to improve gender translation
Mitigation type	Specify the type of mitigation—only if previous field was annotated as either YES or OTHER.<br>• TRAINING: the mitigation strategy implies retraining from scratch<br>• FINE-TUNING: the mitigation strategy implies dedicated fine-tuning<br>• INFERENCE: the mitigation strategy applies at inference time<br>• DOUBLE-OUTPUT-REWRITER: a rewriter is applied a postprocessing step to the MT output to offer an alternative translation<br>• CONTROLLED LLM GENERATION: the mitigaton strategy relies on dedicated prompts to control the realization of gender translation—either as single or double output<br>• COMPARISON: a baseline model for different modalities or architectures (e.g. document-level MT) is proposed to improve gender translation<br>• OTHER: mitigations that do not fit any of the above, e.g., embeddings interventions, approaches to Statistical MT<br>• NA: the label does not apply, no mitigation strategy is proposed<br>.
Paradigm	Which MT paradigm is considered?<br>• SMT: Statistical Machine Translation<br>• NMT: neural machine translation<br>• LLM: large language models<br>• NA: the label does not apply. There is not explicit mention to any model, or focus only on human translation
Intersectional	Which sociodemographic axis are considered for potential bias and discrimination?<br>More than one label can apply.<br>• GENDER, AGE, RACE, RELIGION, SOCIAL CLASS, SEXUAL ORIENTATION

Table S1: Annotation Guidelines.

Supplemental Note: Full List of Reviewed Papers

2016 van der Wees et al.[2], Bawden et al.[3]

2017 Rabinovich et al.[4], Bawden[5]

2018 Popel[6], Vanmassenhove et al.[7], Michel and Neubig[8]

2019 Moryossef et al.[9], Escudé Font and Costa-jussà[10], Cho et al.[11], Stanovsky et al.[12], Habash et al.[13]

2020 Stafanovičs et al.[14], Basta et al.[15], Costa-jussà and de Jorge[16], Saunders et al.[17], Gonen and Webster[18], Gaido et al.[19], Stojanovski et al.[20], Rescigno et al.[21], Bentivogli et al.[22], Saunders and Byrne[23], Hovy et al.[24], González et al.[25], Kocmi et al.[26], Costa-jussà et al.[27], Caglayan et al.[28]

2021 Troles and Schmid[29], Savoldi et al.[30], Wisniewski et al.[31], Ciora et al.[32], Escolano et al.[33], Ramesh et al.[34], Levy et al.[35], Choubey et al.[36], Gaido et al.[37], Vanmassenhove et al.[38], Popović[39], Vincent[40], Renduchintala et al.[41], Castilho et al.[42], Wisniewski et al.[43], Jain et al.[44], Vamvas and Sennrich[45], Vanmassenhove et al.[46], Vanmassenhove and Monti[47]

2022 Wisniewski et al.[48], Costa-jussà et al.[49], Castilho[50], Gete et al.[51], Sólmundsdóttir et al.[52], Savoldi et al.[53], Měchura[54], Corral and Saralegi[55], Mohammadshahi et al.[56], Saunders et al.[57], Currey et al.[58], Karpinska et al.[59], Zhu et al.[60], Sharma et al.[61], Wisniewski et al.[62], Vincent et al.[63], Daems and Hackenbuchner[64], Wang et al.[65], Renduchintala and Williams[66], Wairagala et al.[67], Savoldi et al.[68], Alrowili and Shanker[69], Alhafni et al.[70],[71]

2023 Savoldi et al.[72], Gete and Etchegoyhen[73], Dinh and Niehues[74], Singh[75], Iluz et al.[76], Alhafni et al.[77], Sandoval et al.[78], Wicks and Post[79], Triboulet and Bouillon[80], Gromann et al.[81], Paolucci et al.[82], Lardelli and Gromann[83], Piergentili et al.[84], Saunders and Olsen[85], Kostikova et al.[86], Costa-jussà et al.[87],[88], Cabrera and Niehues[89], Daems[90], Fucci et al.[91], Piergentili et al.[92], Lu et al.[93], Castilho et al.[94], Paulo et al.[95], Le et al.[96], Sarti et al.[97], Lauscher et al.[98], Vincent et al.[99], Attanasio et al.[100], Lee et al.[101], Wang et al.[102], Veloso et al.[103], Amrhein et al.[104], Soler Uguet et al.[105], Sarti et al.[106]

2024 Savoldi et al.[107], Liu and Niehues[108], Mash et al.[109], Lee et al.[110], Costa-jussà et al.[111], Daems[112], Friðriksdóttir[113], Garg et al.[114], Gete and Etchegoyhen[115], Hackenbuchner et al.[116], Iluz et al.[117], Lardelli et al.[118],[119], Luthra and Nijman[120], Nunziatini and Diego[121], Piergentili et al.[122], Pikuliak et al.[123], Popovic and Lapshinova-Koltunski[124], Rarrick et al.[125], Robinson et al.[126], Sánchez et al.[127], Sant et al.[128], Savoldi et al.[129],[130], Sewunetie et al.[131], Stewart and Mihalcea[132], Üstün et al.[133], Zerva et al.[134]

Supplemental Method: Paper Selection Procedure for Literature Review

Our paper selection procedure was systematic and based on peer-reviewed papers in the ACL Anthology that mentioned machine translation (and paraphrases of that) as well as "gender" (and/or "bias") in the title and/or abstract. We kept 133 papers that were considered in-scope based on the following parameters:

- MT needs to be the main focus of the paper, not a method towards a different goal. Based on this criterion, we excluded for example papers such as Adelani et al.[135]
- MT can be text-to-text as well as other modalities (e.g., speech-to-text).
- Rewriters for MT output were considered in scope, as they are a popular method to improve output, e.g., to make it more inclusive.
- Even if MT is not part of the core experiments, but the findings are overall intended to also inform and guide more inclusive MT such as in Paolucci et al.[82], then we considered the paper in-scope.
- (Human) gender translation needs to be in focus in the paper. This can be gender translation, gender bias, fairness, etc.

Mind that the above search selection method naturally includes all relevant LLM papers , as they need to focus on translation, and hence are expected to mention "translation" in their title or abstract.

Supplemental References

[S1] Bentivogli, L., Savoldi, B., Negri, M., Di Gangi, M. A., Cattoni, R., and Turchi, M. (2020). Gender in danger? evaluating speech translation technology on the MuST-SHE corpus. Proceedings of the 58th Annual Meeting of the Association for Computational Linguistics (ACL). (6923–6933). `10.18653/v1/2020.acl-main.619`.

[S2] van der Wees, M., Bisazza, A., and Monz, C. (2016). Measuring the effect of conversational aspects on machine translation quality. Proceedings of COLING 2016, the 26th International Conference on Computational Linguistics: Technical Papers. (2571–2581).

[S3] Bawden, R., Wisniewski, G., and Maynard, H. (2016). Investigating gender adaptation for speech translation. Actes de la conférence conjointe JEP-TALN-RECITAL 2016. volume 2 : TALN (Posters). (490–497).

[S4] Rabinovich, E., Patel, R. N., Mirkin, S., Specia, L., and Wintner, S. (2017). Personalized machine translation: Preserving original author traits. Proceedings of the 15th Conference of the European Chapter of the Association for Computational Linguistics: Volume 1, Long Papers. Valencia, Spain (1074–1084). 10.18653/v1/E17-1101.

[S5] Bawden, R. (2017). Machine translation of speech-like texts: Strategies for the inclusion of context. Actes des 24ème Conférence sur le Traitement Automatique des Langues Naturelles. 19es REncontres jeunes Chercheurs en Informatique pour le TAL (RECITAL 2017). (1–14).

[S6] Popel, M. (2018). CUNI transformer neural MT system for WMT18. Proceedings of the Third Conference on Machine Translation: Shared Task Papers. (482–487). 10.18653/v1/W18-6424.

[S7] Vanmassenhove, E., Hardmeier, C., and Way, A. (2018). Getting gender right in neural machine translation. Proceedings of the 2018 Conference on Empirical Methods in Natural Language Processing. (3003–3008). 10.18653/v1/D18-1334.

[S8] Michel, P., and Neubig, G. (2018). Extreme adaptation for personalized neural machine translation. Proceedings of the 56th Annual Meeting of the Association for Computational Linguistics (Volume 2: Short Papers). (312–318). 10.18653/v1/P18-2050.

[S9] Moryossef, A., Aharoni, R., and Goldberg, Y. (2019). Filling gender & number gaps in neural machine translation with black-box context injection. Proceedings of the First Workshop on Gender Bias in Natural Language Processing. (49–54). 10.18653/v1/W19-3807.

[S10] Escudé Font, J., and Costa-jussà, M. R. (2019). Equalizing gender bias in neural machine translation with word embeddings techniques. Proceedings of the First Workshop on Gender Bias in Natural Language Processing. (147–154). 10.18653/v1/W19-3821.

[S11] Cho, W. I., Kim, J. W., Kim, S. M., and Kim, N. S. (2019). On measuring gender bias in translation of gender-neutral pronouns. Proceedings of the First Workshop on Gender Bias in Natural Language Processing. (173–181). 10.18653/v1/W19-3824.

[S12] Stanovsky, G., Smith, N. A., and Zettlemoyer, L. (2019). Evaluating gender bias in machine translation. Proceedings of the 57th Annual Meeting of the Association for Computational Linguistics. (1679–1684). 10.18653/v1/P19-1164.

[S13] Habash, N., Bouamor, H., and Chung, C. (2019). Automatic gender identification and reinflection in Arabic. Proceedings of the First Workshop on Gender Bias in Natural Language Processing. (155–165). 10.18653/v1/W19-3822.

[S14] Stafanovičs, A., Bergmanis, T., and Pinnis, M. (2020). Mitigating gender bias in machine translation with target gender annotations. Proceedings of the Fifth Conference on Machine Translation. (629–638).

[S15] Basta, C., Costa-jussà, M. R., and Fonollosa, J. A. R. (2020). Towards mitigating gender bias in a decoder-based neural machine translation model by adding contextual information. Proceedings of the The Fourth Widening Natural Language Processing Workshop. (99–102). 10.18653/v1/2020.winlp-1.25.

[S16] Costa-jussà, M. R., and de Jorge, A. (2020). Fine-tuning neural machine translation on gender-balanced datasets. Proceedings of the Second Workshop on Gender Bias in Natural Language Processing. (26–34).

[S17] Saunders, D., Sallis, R., and Byrne, B. (2020). Neural machine translation doesn't translate gender coreference right unless you make it. Proceedings of the Second Workshop on Gender Bias in Natural Language Processing. (35–43).

[S18] Gonen, H., and Webster, K. (2020). Automatically identifying gender issues in machine translation using perturbations. Findings of the Association for Computational Linguistics: EMNLP 2020. (1991–1995). 10.18653/v1/2020.findings-emnlp.180.

[S19] Gaido, M., Savoldi, B., Bentivogli, L., Negri, M., and Turchi, M. (2020). Breeding gender-aware direct speech translation systems. Proceedings of the 28th International Conference on Computational Linguistics. (3951–3964). 10.18653/v1/2020.coling-main.350.

[S20] Stojanovski, D., Krojer, B., Peskov, D., and Fraser, A. (2020). ContraCAT: Contrastive coreference analytical templates for machine translation. Proceedings of the 28th International Conference on Computational Linguistics. (4732–4749). 10.18653/v1/2020.coling-main.417.

[S21] Rescigno, A. A., Monti, J., Way, A., and Vanmassenhove, E. (2020). A case study of natural gender phenomena in translation: A comparison of Google Translate, bing Microsoft translator and DeepL for English to Italian, French and Spanish. Workshop on the Impact of Machine Translation (iMpacT 2020). (62–90).

[S22] Bentivogli, L., Savoldi, B., Negri, M., Di Gangi, M. A., Cattoni, R., and Turchi, M. (2020). Gender in danger? evaluating speech translation technology on the MuST-SHE corpus. Proceedings of the 58th Annual Meeting of the Association for Computational Linguistics. (6923–6933). 10.18653/v1/2020.acl-main.619.

[S23] Saunders, D., and Byrne, B. (2020). Reducing gender bias in neural machine translation as a domain adaptation problem. Proceedings of the 58th Annual Meeting of the Association for Computational Linguistics. (7724–7736). 10.18653/v1/2020.acl-main.690.

[S24] Hovy, D., Bianchi, F., and Fornaciari, T. (2020). "you sound just like your father" commercial machine translation systems include stylistic biases. Proceedings of the 58th Annual Meeting of the Association for Computational Linguistics. (1686–1690). 10.18653/v1/2020.acl-main.154.

[S25] González, A. V., Barrett, M., Hvingelby, R., Webster, K., and Søgaard, A. (2020). Type B reflexivization as an unambiguous testbed for multilingual multi-task gender bias. Proceedings of the 2020 Conference on Empirical Methods in Natural Language Processing (EMNLP). (2637–2648). 10.18653/v1/2020.emnlp-main.209.

[S26] Kocmi, T., Limisiewicz, T., and Stanovsky, G. (2020). Gender coreference and bias evaluation at WMT 2020. Proceedings of the Fifth Conference on Machine Translation. (357–364).

[S27] Costa-jussà, M. R., Li Lin, P., and España-Bonet, C. (2020). GeBioToolkit: Automatic extraction of gender-balanced multilingual corpus of Wikipedia biographies. Proceedings of the Twelfth Language Resources and Evaluation Conference. (4081–4088).

[S28] Caglayan, O., Ive, J., Haralampieva, V., Madhyastha, P., Barrault, L., and Specia, L. (2020). Simultaneous machine translation with visual context. Proceedings of the 2020 Conference on Empirical Methods in Natural Language Processing (EMNLP). (2350–2361). 10.18653/v1/2020.emnlp-main.184.

[S29] Troles, J.-D., and Schmid, U. (2021). Extending challenge sets to uncover gender bias in machine translation: Impact of stereotypical verbs and adjectives. Proceedings of the Sixth Conference on Machine Translation. (531–541).

[S30] Savoldi, B., Gaido, M., Bentivogli, L., Negri, M., and Turchi, M. (2021). Gender bias in machine translation. Transactions of the Association for Computational Linguistics *9*, 845–874. 10.1162/tacl_a_00401.

[S31] Wisniewski, G., Zhou, L., Ballier, N., and Yvon, F. (2021). Biais de genre dans un système de traduction automatiqueneuronale : une étude préliminaire (gender bias in neural translation : a preliminary study). Actes de la 28e Conférence sur le Traitement Automatique des Langues Naturelles. Volume 1 : conférence principale. (11–25).

[S32] Ciora, C., Iren, N., and Alikhani, M. (2021). Examining covert gender bias: A case study in Turkish and English machine translation models. Proceedings of the 14th International Conference on Natural Language Generation. (55–63).

[S33] Escolano, C., Ojeda, G., Basta, C., and Costa-jussa, M. R. (2021). Multi-task learning for improving gender accuracy in neural machine translation. Proceedings of the 18th International Conference on Natural Language Processing (ICON). (12–17).

[S34] Ramesh, K., Gupta, G., and Singh, S. (2021). Evaluating gender bias in Hindi-English machine translation. Proceedings of the 3rd Workshop on Gender Bias in Natural Language Processing. (16–23). 10.18653/v1/2021.gebnlp-1.3.

[S35] Levy, S., Lazar, K., and Stanovsky, G. (2021). Collecting a large-scale gender bias dataset for coreference resolution and machine translation. Findings of the Association for Computational Linguistics: EMNLP 2021. (2470–2480). 10.18653/v1/2021.findings-emnlp.211.

[S36] Choubey, P. K., Currey, A., Mathur, P., and Dinu, G. (2021). GFST: Gender-filtered self-training for more accurate gender in translation. Proceedings of the 2021 Conference on Empirical Methods in Natural Language Processing. (1640–1654). 10.18653/v1/2021.emnlp-main.123.

[S37] Gaido, M., Savoldi, B., Bentivogli, L., Negri, M., and Turchi, M. (2021). How to split: The effect of word segmentation on gender bias in speech translation. Findings of the Association for Computational Linguistics: ACL-IJCNLP 2021. (3576–3589). 10.18653/v1/2021.findings-acl.313.

[S38] Vanmassenhove, E., Shterionov, D., and Gwilliam, M. (2021). Machine translationese: Effects of algorithmic bias on linguistic complexity in machine translation. Proceedings of the 16th Conference of the European Chapter of the Association for Computational Linguistics: Main Volume. (2203–2213). 10.18653/v1/2021.eacl-main.188.

[S39] Popović, M. (2021). Agree to disagree: Analysis of inter-annotator disagreements in human evaluation of machine translation output. Proceedings of the 25th Conference on Computational Natural Language Learning. (234–243). 10.18653/v1/2021.conll-1.18.

[S40] Vincent, S. (2021). Towards personalised and document-level machine translation of dialogue. Proceedings of the 16th Conference of the European Chapter of the Association for Computational Linguistics: Student Research Workshop. (137–147). 10.18653/v1/2021.eacl-srw.19.

[S41] Renduchintala, A., Diaz, D., Heafield, K., Li, X., and Diab, M. (2021). Gender bias amplification during speed-quality optimization in neural machine translation. Proceedings of the 59th Annual Meeting of the Association for Computational Linguistics and the 11th International Joint Conference on Natural Language Processing (Volume 2: Short Papers). (99–109). 10.18653/v1/2021.acl-short.15.

[S42] Castilho, S., Cavalheiro Camargo, J. L., Menezes, M., and Way, A. (2021). Dela corpus - a document-level corpus annotated with context-related issues. Proceedings of the Sixth Conference on Machine Translation. Online (566–577).

[S43] Wisniewski, G., Zhu, L., Bailler, N., and Yvon, F. (2021). Screening gender transfer in neural machine translation. Proceedings of the Fourth BlackboxNLP Workshop on Analyzing and Interpreting Neural Networks for NLP. (311–321). 10.18653/v1/2021.blackboxnlp-1.24.

[S44] Jain, N., Popović, M., Groves, D., and Vanmassenhove, E. (2021). Generating gender augmented data for NLP. Proceedings of the 3rd Workshop on Gender Bias in Natural Language Processing. (93–102). 10.18653/v1/2021.gebnlp-1.11.

[S45] Vamvas, J., and Sennrich, R. (2021). Contrastive conditioning for assessing disambiguation in MT: A case study of distilled bias. Proceedings of the 2021 Conference on Empirical Methods in Natural Language Processing. (10246–10265). 10.18653/v1/2021.emnlp-main.803.

[S46] Vanmassenhove, E., Emmery, C., and Shterionov, D. (2021). NeuTral Rewriter: A rule-based and neural approach to automatic rewriting into gender neutral alternatives. Proceedings of the 2021 Conference on Empirical Methods in Natural Language Processing. (8940–8948). 10.18653/v1/2021.emnlp-main.704.

[S47] Vanmassenhove, E., and Monti, J. (2021). gENder-IT: An annotated English-Italian parallel challenge set for cross-linguistic natural gender phenomena. Proceedings of the 3rd Workshop on Gender Bias in Natural Language Processing. (1–7). 10.18653/v1/2021.gebnlp-1.1.

[S48] Wisniewski, G., Zhu, L., Ballier, N., and Yvon, F. (2022). Biais de genre dans un système de traduction automatique neuronale : une étude des mécanismes de transfert cross-langue [gender bias in a neural machine translation system: a study of crosslingual transfer mechanisms]. Traitement Automatique des Langues, Volume 63, Numéro 1 : Varia [Varia]. (37–61).

[S49] Costa-jussà, M. R., Basta, C., and Gállego, G. I. (2022). Evaluating gender bias in speech translation. Proceedings of the Thirteenth Language Resources and Evaluation Conference. (2141–2147). 10.18653/v1/2022.lrec-1.230.

[S50] Castilho, S. (2022). How much context span is enough? examining context-related issues for document-level MT. Proceedings of the Thirteenth Language Resources and Evaluation Conference. (3017–3025).

[S51] Gete, H., Etchegoyhen, T., Ponce, D., Labaka, G., Aranberri, N., Corral, A., Saralegi, X., Ellakuria, I., and Martin, M. (2022). Tando: A corpus for document-level machine translation. Proceedings of the Thirteenth Language Resources and Evaluation Conference. (3026–3037).

[S52] Sólmundsdóttir, A., Guðmundsdóttir, D., Stefánsdóttir, L. B., and Ingason, A. (2022). Mean machine translations: On gender bias in Icelandic machine translations. Proceedings of the Thirteenth Language Resources and Evaluation Conference. (3113–3121).

[S53] Savoldi, B., Gaido, M., Bentivogli, L., Negri, M., and Turchi, M. (2022). On the dynamics of gender learning in speech translation. Proceedings of the 4th Workshop on Gender Bias in Natural Language Processing (GeBNLP). (94–111). 10.18653/v1/2022.gebnlp-1.12.

[S54] Měchura, M. (2022). A taxonomy of bias-causing ambiguities in machine translation. Proceedings of the 4th Workshop on Gender Bias in Natural Language Processing (GeBNLP). (168–173). 10.18653/v1/2022.gebnlp-1.18.

[S55] Corral, A., and Saralegi, X. (2022). Gender bias mitigation for NMT involving genderless languages. Proceedings of the Seventh Conference on Machine Translation (WMT). (165–176).

[S56] Mohammadshahi, A., Nikoulina, V., Berard, A., Brun, C., Henderson, J., and Besacier, L. (2022). What do compressed multilingual machine translation models forget? Findings of the Association for Computational Linguistics: EMNLP 2022. (4308–4329). 10.18653/v1/2022.findings-emnlp.317.

[S57] Saunders, D., Sallis, R., and Byrne, B. (2022). First the worst: Finding better gender translations during beam search. Findings of the Association for Computational Linguistics: ACL 2022. (3814–3823). 10.18653/v1/2022.findings-acl.301.

[S58] Currey, A., Nadejde, M., Pappagari, R. R., Mayer, M., Lauly, S., Niu, X., Hsu, B., and Dinu, G. (2022). MT-GenEval: A counterfactual and contextual dataset for evaluating gender accuracy in machine translation. Proceedings of the 2022 Conference on Empirical Methods in Natural Language Processing. (4287–4299). 10.18653/v1/2022.emnlp-main.288.

[S59] Karpinska, M., Raj, N., Thai, K., Song, Y., Gupta, A., and Iyyer, M. (2022). DEMETR: Diagnosing evaluation metrics for translation. Proceedings of the 2022 Conference on Empirical Methods in Natural Language Processing. (9540–9561). 10.18653/v1/2022.emnlp-main.649.

[S60] Zhu, L., Wisniewski, G., Ballier, N., and Yvon, F. (2022). Flux d'informations dans les systèmes encodeur-décodeur. application à l'explication des biais de genre dans les systèmes de traduction automatique. (information flow in encoder-decoder systems applied to the explanation of gender bias in machine translation systems). Actes de la 29e Conférence sur le Traitement Automatique des Langues Naturelles. Atelier TAL et Humanités Numériques (TAL-HN). (10–18).

[S61] Sharma, S., Dey, M., and Sinha, K. (2022). How sensitive are translation systems to extra contexts? mitigating gender bias in neural machine translation models through relevant contexts. Findings of the Association for Computational Linguistics: EMNLP 2022. (1968–1984). 10.18653/v1/2022.findings-emnlp.143.

[S62] Wisniewski, G., Zhu, L., Ballier, N., and Yvon, F. (2022). Analyzing gender translation errors to identify information flows between the encoder and decoder of a NMT system. Proceedings of the Fifth BlackboxNLP Workshop on Analyzing and Interpreting Neural Networks for NLP. (153–163). 10.18653/v1/2022.blackboxnlp-1.13.

[S63] Vincent, S. T., Barrault, L., and Scarton, C. (2022). Controlling extra-textual attributes about dialogue participants: A case study of English-to-Polish neural machine translation. Proceedings of the 23rd Annual Conference of the European Association for Machine Translation. (121–130).

[S64] Daems, J., and Hackenbuchner, J. (2022). DeBiasByUs: Raising awareness and creating a database of MT bias. Proceedings of the 23rd Annual Conference of the European Association for Machine Translation. (289–290).

[S65] Wang, J., Rubinstein, B., and Cohn, T. (2022). Measuring and mitigating name biases in neural machine translation. Proceedings of the 60th Annual Meeting of the Association for Computational Linguistics (Volume 1: Long Papers). (2576–2590). 10.18653/v1/2022.acl-long.184.

[S66] Renduchintala, A., and Williams, A. (2022). Investigating failures of automatic translationin the case of unambiguous gender. Proceedings of the 60th Annual Meeting of the Association for Computational Linguistics (Volume 1: Long Papers). (3454–3469). 10.18653/v1/2022.acl-long.243.

[S67] Wairagala, E. P., Mukiibi, J., Tusubira, J. F., Babirye, C., Nakatumba-Nabende, J., Katumba, A., and Ssenkungu, I. (2022). Gender bias evaluation in Luganda-English machine translation. Proceedings of the 15th biennial conference of the Association for Machine Translation in the Americas (Volume 1: Research Track). (274–286).

[S68] Savoldi, B., Gaido, M., Bentivogli, L., Negri, M., and Turchi, M. (2022). Under the morphosyntactic lens: A multifaceted evaluation of gender bias in speech translation. Proceedings of the 60th Annual Meeting of the Association for Computational Linguistics (Volume 1: Long Papers). (1807–1824). 10.18653/v1/2022.acl-long.127.

[S69] Alrowili, S., and Shanker, V. (2022). Generative approach for gender-rewriting task with arabict5. Proceedings of the Seventh Arabic Natural Language Processing Workshop (WANLP). (491–495). 10.18653/v1/2022.wanlp-1.55.

[S70] Alhafni, B., Habash, N., and Bouamor, H. (2022). User-centric gender rewriting. Proceedings of the 2022 Conference of the North American Chapter of the Association for Computational Linguistics: Human Language Technologies. (618–631). 10.18653/v1/2022.naacl-main.46.

[S71] Alhafni, B., Habash, N., and Bouamor, H. (2022). The Arabic parallel gender corpus 2.0: Extensions and analyses. Proceedings of the Thirteenth Language Resources and Evaluation Conference. (1870–1884).

[S72] Savoldi, B., Gaido, M., Negri, M., and Bentivogli, L. (2023). Test suites task: Evaluation of gender fairness in MT with MuST-SHE and INES. Proceedings of the Eighth Conference on Machine Translation. (252–262). 10.18653/v1/2023.wmt-1.25.

[S73] Gete, H., and Etchegoyhen, T. (2023). An evaluation of source factors in concatenation-based context-aware neural machine translation. Proceedings of the 14th International Conference on Recent Advances in Natural Language Processing. (399–407).

[S74] Dinh, T. A., and Niehues, J. (2023). Perturbation-based QE: An explainable, unsupervised word-level quality estimation method for blackbox machine translation. Proceedings of Machine Translation Summit XIX, Vol. 1: Research Track. (59–71).

[S75] Singh, P. (2023). Gender inflected or bias inflicted: On using grammatical gender cues for bias evaluation in machine translation. Proceedings of the 13th International Joint Conference on Natural Language Processing and the 3rd Conference of the Asia-Pacific Chapter of the Association for Computational Linguistics: Student Research Workshop. (17–23). 10.18653/v1/2023.ijcnlp-srw.3.

[S76] Iluz, B., Limisiewicz, T., Stanovsky, G., and Mareček, D. (2023). Exploring the impact of training data distribution and subword tokenization on gender bias in machine translation. Proceedings of the 13th International Joint Conference on Natural Language Processing and the 3rd Conference of the Asia-Pacific Chapter of the Association for Computational Linguistics (Volume 1: Long Papers). (885–896). 10.18653/v1/2023.ijcnlp-main.57.

[S77] Alhafni, B., Obeid, O., and Habash, N. (2023). The user-aware Arabic gender rewriter. Proceedings of the First Workshop on Gender-Inclusive Translation Technologies. (3–11).

[S78] Sandoval, S., Zhao, J., Carpuat, M., and Daumé III, H. (2023). A rose by any other name would not smell as sweet: Social bias in names mistranslation. Proceedings of the 2023 Conference on Empirical Methods in Natural Language Processing. (3933–3945). 10.18653/v1/2023.emnlp-main.239.

[S79] Wicks, R., and Post, M. (2023). Identifying context-dependent translations for evaluation set production. Proceedings of the Eighth Conference on Machine Translation. (452–467). 10.18653/v1/2023.wmt-1.42.

[S80] Triboulet, B., and Bouillon, P. (2023). Evaluating the impact of stereotypes and language combinations on gender bias occurrence in NMT generic systems. Proceedings of the Third Workshop on Language Technology for Equality, Diversity and Inclusion. (62–70).

[S81] Gromann, D., Lardelli, M., Spiel, K., Burtscher, S., Klausner, L. D., Mettinger, A., Miladinovic, I., Schefer-Wenzl, S., Duh, D., and Bühn, K. (2023). Participatory research as a path to community-informed, gender-fair machine translation. Proceedings of the First Workshop on Gender-Inclusive Translation Technologies. (49–59).

[S82] Paolucci, A. B., Lardelli, M., and Gromann, D. (2023). Gender-fair language in translation: A case study. Proceedings of the First Workshop on Gender-Inclusive Translation Technologies. (13–23).

[S83] Lardelli, M., and Gromann, D. (2023). Gender-fair post-editing: A case study beyond the binary. Proceedings of the 24th Annual Conference of the European Association for Machine Translation. (251–260).

[S84] Piergentili, A., Fucci, D., Savoldi, B., Bentivogli, L., and Negri, M. (2023). Gender neutralization for an inclusive machine translation: from theoretical foundations to open challenges. Proceedings of the First Workshop on Gender-Inclusive Translation Technologies. (71–83).

[S85] Saunders, D., and Olsen, K. (2023). Gender, names and other mysteries: Towards the ambiguous for gender-inclusive translation. Proceedings of the First Workshop on Gender-Inclusive Translation Technologies. (85–93).

[S86] Kostikova, A., Daems, J., and Lazarov, T. (2023). How adaptive is adaptive machine translation, really? a gender-neutral language use case. Proceedings of the First Workshop on Gender-Inclusive Translation Technologies. (95–97).

[S87] Costa-jussà, M., Smith, E., Ropers, C., Licht, D., Maillard, J., Ferrando, J., and Escolano, C. (2023). Toxicity in multilingual machine translation at scale. Findings of the Association for Computational Linguistics: EMNLP 2023. (9570–9586). 10.18653/v1/2023.findings-emnlp.642.

[S88] Costa-jussà, M., Andrews, P., Smith, E., Hansanti, P., Ropers, C., Kalbassi, E., Gao, C., Licht, D., and Wood, C. (2023). Multilingual holistic bias: Extending descriptors and patterns to unveil demographic biases in languages at scale. Proceedings of the 2023 Conference on Empirical Methods in Natural Language Processing. (14141–14156). 10.18653/v1/2023.emnlp-main.874.

[S89] Cabrera, L., and Niehues, J. (2023). Gender lost in translation: How bridging the gap between languages affects gender bias in zero-shot multilingual translation. Proceedings of the First Workshop on Gender-Inclusive Translation Technologies. (25–35).

[S90] Daems, J. (2023). Gender-inclusive translation for a gender-inclusive sport: strategies and translator perceptions at the international quadball association. Proceedings of the First Workshop on Gender-Inclusive Translation Technologies. (37–47).

[S91] Fucci, D., Gaido, M., Papi, S., Cettolo, M., Negri, M., and Bentivogli, L. (2023). Integrating language models into direct speech translation: An inference-time solution to control gender inflection. Proceedings of the 2023 Conference on Empirical Methods in Natural Language Processing. Singapore (11505–11517). 10.18653/v1/2023.emnlp-main.705.

[S92] Piergentili, A., Savoldi, B., Fucci, D., Negri, M., and Bentivogli, L. (2023). Hi guys or hi folks? benchmarking gender-neutral machine translation with the GeNTE corpus. Proceedings of the 2023 Conference on Empirical Methods in Natural Language Processing. (14124–14140). 10.18653/v1/2023.emnlp-main.873.

[S93] Lu, T., Aepli, N., and Rios, A. (2023). Reducing gender bias in NMT with FUDGE. Proceedings of the First Workshop on Gender-Inclusive Translation Technologies. (61–69).

[S94] Castilho, S., Mallon, C. Q., Meister, R., and Yue, S. (2023). Do online machine translation systems care for context? what about a gpt model? Proceedings of the 24th Annual Conference of the European Association for Machine Translation. (393–417).

[S95] Paulo, M., Cabarrão, V., Moniz, H., Menezes, M., Grewcock, R., and Farah, E. (2023). Context-aware and gender-neutral translation memories. Proceedings of the 24th Annual Conference of the European Association for Machine Translation. (437–444).

[S96] Le, N. T., Hansal, O., and Sadat, F. (2023). Challenges and issue of gender bias in under-represented languages: An empirical study on Inuktitut-English NMT. Proceedings of the Sixth Workshop on the Use of Computational Methods in the Study of Endangered Languages. (89–97).

[S97] Sarti, G., Htut, P. M., Niu, X., Hsu, B., Currey, A., Dinu, G., and Nadejde, M. (2023). RAMP: Retrieval and attribute-marking enhanced prompting for attribute-controlled translation. Proceedings of the 61st Annual Meeting of the Association for Computational Linguistics (Volume 2: Short Papers). (1476–1490). 10.18653/v1/2023.acl-short.126.

[S98] Lauscher, A., Nozza, D., Miltersen, E., Crowley, A., and Hovy, D. (2023). What about “em”? how commercial machine translation fails to handle (neo-)pronouns. Proceedings of the 61st Annual Meeting of the Association for Computational Linguistics (Volume 1: Long Papers). (377–392). 10.18653/v1/2023.acl-long.23.

[S99] Vincent, S., Flynn, R., and Scarton, C. (2023). Mtcue: Learning zero-shot control of extra-textual attributes by leveraging unstructured context in neural machine translation. Findings of the Association for Computational Linguistics: ACL 2023. Toronto, Canada (8210–8226). 10.18653/v1/2023.findings-acl.521.

[S100] Attanasio, G., Plaza del Arco, F. M., Nozza, D., and Lauscher, A. (2023). A tale of pronouns: Interpretability informs gender bias mitigation for fairer instruction-tuned machine translation. Proceedings of the 2023 Conference on Empirical Methods in Natural Language Processing. (3996–4014). 10.18653/v1/2023.emnlp-main.243.

[S101] Lee, M., Koh, H., Lee, K.-i., Zhang, D., Kim, M., and Jung, K. (2023). Target-agnostic gender-aware contrastive learning for mitigating bias in multilingual machine translation. Proceedings of the 2023 Conference on Empirical Methods in Natural Language Processing. (16825–16839). 10.18653/v1/2023.emnlp-main.1046.

[S102] Wang, L., Liu, S., Xu, M., Song, L., Shi, S., and Tu, Z. (2023). A survey on zero pronoun translation. Proceedings of the 61st Annual Meeting of the Association for Computational Linguistics (Volume 1: Long Papers). (3325–3339). 10.18653/v1/2023.acl-long.187.

[S103] Veloso, L., Coheur, L., and Ribeiro, R. (2023). A rewriting approach for gender inclusivity in Portuguese. Findings of the Association for Computational Linguistics: EMNLP 2023. (8747–8759). 10.18653/v1/2023.findings-emnlp.585.

[S104] Amrhein, C., Schottmann, F., Sennrich, R., and Läubli, S. (2023). Exploiting biased models to de-bias text: A gender-fair rewriting model. Proceedings of the 61st Annual Meeting of the Association for Computational Linguistics (Volume 1: Long Papers). (4486–4506). 10.18653/v1/2023.acl-long.246.

[S105] Soler Uguet, C., Bane, F., Aymo, M., Fernandes Torres, J. P., Zaretskaya, A., and Blanch Miró, T. (2023). Enhancing gender representation in neural machine translation: A comparative analysis of annotating strategies for English-Spanish and English-Polish language pairs. Proceedings of Machine Translation Summit XIX, Vol. 2: Users Track. (171–172).

[S106] Sarti, G., Feldhus, N., Sickert, L., and van der Wal, O. (2023). Inseq: An interpretability toolkit for sequence generation models. Proceedings of the 61st Annual Meeting of the Association for Computational Linguistics (Volume 3: System Demonstrations). (421–435). 10.18653/v1/2023.acl-demo.40.

[S107] Savoldi, B., Piergentili, A., Fucci, D., Negri, M., and Bentivogli, L. (2024). A prompt response to the demand for automatic gender-neutral translation. Proceedings of the 18th Conference of the European Chapter of the Association for Computational Linguistics (Volume 2: Short Papers). (256–267).

[S108] Liu, D., and Niehues, J. (2024). How transferable are attribute controllers on pretrained multilingual translation models? Proceedings of the 18th Conference of the European Chapter of the Association for Computational Linguistics (Volume 1: Long Papers). (334–348).

[S109] Mash, A., Escolano, C., Sant, A., Melero, M., and de Luca Fornaciari, F. (2024). Unmasking biases: Exploring gender bias in English-Catalan machine translation through tokenization analysis and novel dataset. Proceedings of the 2024 Joint International Conference on Computational Linguistics, Language Resources and Evaluation (LREC-COLING 2024). (17144–17153).

[S110] Lee, M., Koh, H., Kim, M., and Jung, K. (2024). Fine-grained gender control in machine translation with large language models. Proceedings of the 2024 Conference of the North American Chapter of the Association for Computational Linguistics: Human Language Technologies (Volume 1: Long Papers). (5416–5430).

[S111] Costa-jussà, M., Andrews, P., Basta, C., Ciro, J., Falenska, A., Goldfarb-Tarrant, S., Mosquera, R., Nozza, D., and Sánchez, E. (2024). Overview of the shared task on machine translation gender bias evaluation with multilingual holistic bias. Proceedings of the 5th Workshop on Gender Bias in Natural Language Processing (GeBNLP). (399–404). `10.18653/v1/2024.gebnlp-1.26`.

[S112] Daems, J. (2024). Pilot testing gender-inclusive translations and machine translations for German quadball referee certification test takers. Proceedings of the 2nd International Workshop on Gender-Inclusive Translation Technologies. (56–57).

[S113] Friðriksdóttir, S. R. (2024). The GenderQueer test suite. Proceedings of the Ninth Conference on Machine Translation. (327–340). `10.18653/v1/2024.wmt-1.26`.

[S114] Garg, S., Gheini, M., Emmanuel, C., Likhomanenko, T., Gao, Q., and Paulik, M. (2024). Generating gender alternatives in machine translation. Proceedings of the 5th Workshop on Gender Bias in Natural Language Processing (GeBNLP). (237–254). `10.18653/v1/2024.gebnlp-1.15`.

[S115] Gete, H., and Etchegoyhen, T. (2024). Does context help mitigate gender bias in neural machine translation? Findings of the Association for Computational Linguistics: EMNLP 2024. (14788–14794). `10.18653/v1/2024.findings-emnlp.868`.

[S116] Hackenbuchner, J., Daems, J., Tezcan, A., and Maladry, A. (2024). You shall know a word‘s gender by the company it keeps: Comparing the role of context in human gender assumptions with MT. Proceedings of the 2nd International Workshop on Gender-Inclusive Translation Technologies. (31–41).

[S117] Iluz, B., Elazar, Y., Yehudai, A., and Stanovsky, G. (2024). Applying intrinsic debiasing on downstream tasks: Challenges and considerations for machine translation. Proceedings of the 2024 Conference on Empirical Methods in Natural Language Processing. (14914–14921). `10.18653/v1/2024.emnlp-main.829`.

[S118] Lardelli, M., Attanasio, G., and Lauscher, A. (2024). Building bridges: A dataset for evaluating gender-fair machine translation into German. Findings of the Association for Computational Linguistics: ACL 2024. (7542–7550). `10.18653/v1/2024.findings-acl.448`.

[S119] Lardelli, M., Dill, T., Attanasio, G., and Lauscher, A. (2024). Sparks of fairness: Preliminary evidence of commercial machine translation as English-to-German gender-fair dictionaries. Proceedings of the 2nd International Workshop on Gender-Inclusive Translation Technologies. (12–21).

[S120] Luthra, M., and Nijman, B. (2024). Lost in translation? approaches to gender representation in multilingual archives. Proceedings of the 2nd International Workshop on Gender-Inclusive Translation Technologies. (42–55).

[S121] Nunziatini, M., and Diego, S. (2024). Implementing gender-inclusivity in mt output using automatic post-editing with llms. Proceedings of the 25th Annual Conference of the European Association for Machine Translation (Volume 1). (580–589).

[S122] Piergentili, A., Savoldi, B., Negri, M., and Bentivogli, L. (2024). Enhancing gender-inclusive machine translation with neomorphemes and large language models. Proceedings of the 25th Annual Conference of the European Association for Machine Translation (Volume 1). (300–314).

[S123] Pikuliak, M., Oresko, S., Hrckova, A., and Simko, M. (2024). Women are beautiful, men are leaders: Gender stereotypes in machine translation and language modeling. Findings of the Association for Computational Linguistics: EMNLP 2024. (3060–3083). 10.18653/v1/2024.findings-emnlp.173.

[S124] Popovic, M., and Lapshinova-Koltunski, E. (2024). Gender and bias in Amazon review translations: by humans, MT systems and ChatGPT. Proceedings of the 2nd International Workshop on Gender-Inclusive Translation Technologies. (22–30).

[S125] Rarrick, S., Naik, R., Poudel, S., and Chowdhary, V. (2024). GATE X-E : A challenge set for gender-fair translations from weakly-gendered languages. Findings of the Association for Computational Linguistics: ACL 2024. (8526–8546). 10.18653/v1/2024.findings-acl.504.

[S126] Robinson, K., Kudugunta, S., Stella, R., Dev, S., and Bastings, J. (2024). MiTTenS: A dataset for evaluating gender mistranslation. Proceedings of the 2024 Conference on Empirical Methods in Natural Language Processing. (4115–4124). 10.18653/v1/2024.emnlp-main.238.

[S127] Sánchez, E., Andrews, P., Stenetorp, P., Artetxe, M., and Costa-jussà, M. R. (2024). Gender-specific machine translation with large language models. Proceedings of the Fourth Workshop on Multilingual Representation Learning (MRL 2024). (148–158). 10.18653/v1/2024.mrl-1.10.

[S128] Sant, A., Escolano, C., Mash, A., De Luca Fornaciari, F., and Melero, M. (2024). The power of prompts: Evaluating and mitigating gender bias in MT with LLMs. Proceedings of the 5th Workshop on Gender Bias in Natural Language Processing (GeBNLP). (94–139). 10.18653/v1/2024.gebnlp-1.7.

[S129] Savoldi, B., Gaido, M., Negri, M., and Bentivogli, L. (2024). FBK@IWSLT test suites task: Gender bias evaluation with MuST-SHE. Proceedings of the 21st International Conference on Spoken Language Translation (IWSLT 2024). (65–71). 10.18653/v1/2024.iwslt-1.10.

[S130] Savoldi, B., Papi, S., Negri, M., Guerberof-Arenas, A., and Bentivogli, L. (2024). What the harm? quantifying the tangible impact of gender bias in machine translation with a human-centered study. Proceedings of the 2024 Conference on Empirical Methods in Natural Language Processing. (18048–18076). 10.18653/v1/2024.emnlp-main.1002.

[S131] Sewunetie, W., Tonja, A., Belay, T., Nigatu, H. H., Gebremeskel, G., Mossie, Z., Seid, H., and Yimam, S. (2024). Gender bias evaluation in machine translation for Amharic, Tigrigna, and afaan oromoo. Proceedings of the 2nd International Workshop on Gender-Inclusive Translation Technologies. (1–11).

[S132] Stewart, I., and Mihalcea, R. (2024). Whose wife is it anyway? assessing bias against same-gender relationships in machine translation. Proceedings of the 5th Workshop on Gender Bias in Natural Language Processing (GeBNLP). (365–375). 10.18653/v1/2024.gebnlp-1.23.

[S133] Üstün, A., Aryabumi, V., Yong, Z., Ko, W.-Y., D'souza, D., Onilude, G., Bhandari, N., Singh, S., Ooi, H.-L., Kayid, A., Vargus, F., Blunsom, P., Longpre, S., Muennighoff, N., Fadaee, M., Kreutzer, J., and Hooker, S. (2024). Aya model: An instruction finetuned open-access multilingual language model. Proceedings of the 62nd Annual Meeting of the Association for Computational Linguistics (Volume 1: Long Papers). (15894–15939). 10.18653/v1/2024.acl-long.845.

[S134] Zerva, C., Blain, F., C. De Souza, J. G., Kanojia, D., Deoghare, S., Guerreiro, N. M., Attanasio, G., Rei, R., Orasan, C., Negri, M., Turchi, M., Chatterjee, R., Bhattacharyya, P., Freitag, M., and Martins, A. (2024). Findings of the quality estimation shared task at WMT 2024: Are LLMs closing the gap in QE? Proceedings of the Ninth Conference on Machine Translation. (82–109). 10.18653/v1/2024.wmt-1.3.

[S135] Adelani, D., Zhang, M., Shen, X., Davody, A., Kleinbauer, T., and Klakow, D. (2021). Preventing author profiling through zero-shot multilingual back-translation. Proceedings of the 2021 Conference on Empirical Methods in Natural Language Processing. (8687–8695). `10.18653/v1/2021.emnlp-main.684`.
