# Supplementary material for: A decade of gender bias in machine translation
Source: Patterns (N Y). 2025 May 2;6(6):101257. doi: 10.1016/j.patter.2025.101257 (PMC12191736; doi:10.1016/j.patter.2025.101257)
Supplement: Document S2. Article plus supplemental information [file mmc2.pdf]

Patterns

CellPress
OPEN ACCESS

Review

A decade of gender bias in machine translation

Beatrice Savoldi,[1,4,*] Jasmijn Bastings,[2,4,*] Luisa Bentivogli,[1] and Eva Vanmassenhove[3]
[1]Fondazione Bruno Kessler, Trento, Italy
[2]Google DeepMind, Amsterdam, the Netherlands
[3]Tilburg University, Tilburg, The Netherlands
[4]These authors contributed equally
*Correspondence: bsavoldi@fbk.eu (B.S.), bastings@google.com (J.B.)
https://doi.org/10.1016/j.patter.2025.101257

THE BIGGER PICTURE A decade has passed since the first recognition of gender bias in machine translation in a seminal paper by Prof. Londa Schiebinger. Today, multilingual language technology, especially with the rise of advanced AI assistants powered by large language models (LLMs), increasingly shapes global communication and user interactions. Ensuring such technology is inclusive and does not result in harm has become critical, as gender bias in machine translation can lead to unequal representation, misgendering, and tangible service disparities that disproportionately affect marginalized groups.

In this paper, we take stock of the last decade of research on gender bias in machine translation. We find that early optimism about a quick technological solution has given way to a more nuanced picture. Promising trends have emerged—the number of research efforts is growing, and the recognition of non-binary gender identities has improved—but significant challenges persist. These include an overemphasis on English-centric approaches and a tendency to disconnect translation technologies from the context in which they operate. While these efforts have made contributions, we argue that bias is dynamic, multifaceted, and resistant to simple solutions. We build upon the lessons of the past decade to discuss the current landscape in which LLMs are emerging. Our aim is to inspire future work in the field that transcends current limitations.

SUMMARY

Gender bias in machine translation (MT) has been studied for over a decade, a time marked by societal, linguistic, and technological shifts. With the early optimism for a quick solution in mind, we review over 100 studies on the topic and uncover a more complex reality—one that resists a simple technical fix. While we identify key trends and advancements, persistent gaps remain. We argue that there is no simple technical solution to bias. Building on insights from our review, we examine the growing prominence of large language models and discuss the challenges and opportunities they present in the context of gender bias and translation. By doing so, we hope to inspire future work in the field to break with past limitations and to be less focused on a technical fix; more user-centric, multilingual, and multiculturally diverse; more personalized; and better grounded in real-world needs.

INTRODUCTION

Last year marked a decade since Prof. L. Schiebinger[1] published a call to action regarding how scientific research—from car design to drug discovery—should take gender into account lest it lead to the creation of socially harmful, male-centered products. In the piece, she explicitly mentions gender bias in translation technology and how it defaults to masculine pronouns "because *he* occurs more often on the web." She then writes:

> I invited Google and several language-processing experts to a Gendered Innovations workshop at Harvard University in Cambridge, Massachusetts. They listened to the problem for about 20 minutes, then said: "We can fix that!" Although it is complicated, the search for solutions is on.

Now, 10 years later, we ponder: was the "problem" fixed? How far has the search for a solution for gender bias in machine translation (MT) actually come? Instead of treating gender in MT as a cross-linguistic and modeling challenge only, Schiebinger[1] marked a symbolic shift by highlighting bias in MT as a deeper social and technical issue linked to broader gender inequalities, also propagated through technologies. One notorious example is the Finnish sentence "*Hän* on lääkäri. *Hän* on sairaanhoitaja" which is automatically rendered into "*He* is a doctor. *She* is a nurse" (run with DeepL and Google Translate on 20-02-2025).

This is an open access article under the CC BY license (http://creativecommons.org/licenses/by/4.0/).

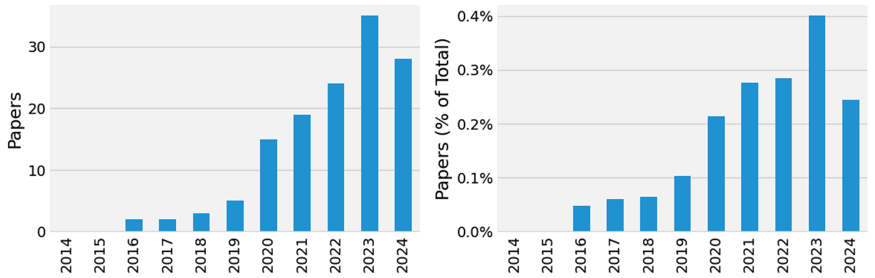

Figure 1. Increasing interest in gender bias and machine translation
The number of publications on gender and translation has generally increased over the years, showing an upward trend until 2024 both in absolute numbers (left) and relative numbers (right).

Although "hän" is gender neutral in Finnish, the output assigns gendered pronouns that reflect well-known occupational stereotypes—even though the original sentence could, in principle, be rendered with any form in English.

Indeed, much has happened in the last decade. Societal norms and attitudes evolved alongside language. LGBTIQ+ people began living more openly, and words such as "family" and "sex" took on new meanings.[2] The use of singular "they" and other linguistic innovations often used by non-binary people rose in popularity,[3,4] while governments and institutions developed guides for inclusive language to drive and keep up with language change. At the same time, innovations in translation technology happened at incredible speed, as shown by a total of three translation paradigms during this period: the decline of phrase-based statistical MT,[5] the advancement of more powerful neural models,[6] and finally the rise of general-purpose large language models (LLMs) with multitask and multilingual capabilities.[7–9]

As these societal and technological changes unfolded, a growing awareness emerged—both within and beyond the research community—of how language technology does not serve all social groups equally.[10] Such concern has become more urgent with widely deployed automatic translations reaching the public at an unprecedented scale. This comes with the risk of disproportionately disfavoring users from marginalized groups,[11,12] with downstream harms ranging from the erasure of non-binary identities[13] to tangible service disparities that incur additional revisions and economic costs to obtain accurate feminine translations.[14] As a matter of fact, these concerns and growing awareness seem to be reflected in research production. Figure 1 shows a general increase in papers focusing on gender bias in MT—with a peak in 2023—suggesting that progress has been made.

In light of the above, in this paper we use Schiebinger's[1] call to action as a symbolic watershed to take stock of the trajectory of research on gender bias in MT of the past decade and return to our main question: did we fix the problem? Given the field's rapid evolution, we contribute to prior efforts to systematize the state of research in monolingual[15,16] and cross-lingual[17] language technologies by integrating the recent rise of LLMs within this landscape. Crucially, instead of providing a static picture, our analysis is also enriched by a diachronic perspective, unpacking how several aspects of gender bias in MT (e.g., non-binary focus, type of mitigation strategies) have been approached over the years.

The paper is structured as follows. First, we provide a brief background on gender and language as well as on MT. In the following section, we conduct a systematic review of the last decade of research on gender bias in MT (133 papers) and distill key findings by identifying promising trends but also persistent gaps. Building on the lessons and picture obtained from our review, we argue that gender bias resists quick technical fixes and that the path toward more inclusive MT is a moving target. To conclude, we discuss challenges and opportunities for the future, particularly as LLMs take center stage in translation technology.

BACKGROUND

Before systematizing current research on gender bias in MT, we first provide the necessary background on MT technology, as well as on the relation between gender and language.

MT advances

MT—as a long-standing application in the field of natural language processing (NLP) and computational linguistics—is the task of automatically rendering content from one language into another language. In the last few years, MT core technology has evolved rapidly, attesting a shift toward new architectures and more powerful solutions that have expanded its overall quality and coverage, thus fostering wider adoption. First, phrase-based statistical MT (SMT)[5] was overtaken by neural MT (NMT)[18–20] powered first by recurrent layers[21,22] and attention mechanisms,[23] then later by transformer layers.[6] More recently, we saw the rise of powerful, decoder-only large language models[7–9]—as well as some encoder-decoder ones such as mT5[24]—that have revolutionized generative tasks. Base LLMs, sometimes referred to as foundation models, can support other modalities such as audio and video and then be further specialized, e.g., through supervised fine-tuning (SFT) and instruction tuning (IT). For LLMs, translation is often one of many possible supported tasks that become available after IT, and, where SMT and NMT are characterized by supervised training on a vast amount of parallel data, LLMs tend to be trained mostly on monolingual data, sometimes using a data mixture that consists of multiple languages. LLMs have been used for translation in zero-shot and few-shot settings, proving increasing MT capabilities.[25] Beside, instruction fine-tuning multilingual LLMs with a controlled injection of parallel data is emerging as a strong contender to dedicated supervised NMT models.[26–28]

Gender and language

Gender, in the context of human referents, is a linguistic category that bears a complex relationship with its extra-linguistic reality.[29] Gendered features in language interact with sociocultural and political perceptions as well as representations of individuals,[30–32] thus prompting discussion on the appropriate

Table 1. ACL Anthology search results for each keyword combination

	Keywords	# Papers
	translation, NMT, MT, rewriter	–
and	gender	168
or	bias	140
	in-scope papers	133

The queries returned 308 results, of which 175 were discarded as out of scope.

recognition of gender groups and their linguistic visibility.[33] For instance, grammatical gender languages such as German, Spanish, and Italian—where gendered morphology is extensive—have long battled the recognition of feminine titles in the professional realm (e.g., President → es: La Presidenta F vs. El Presidente M).[34,35] Indeed, prior work has shown how the use of masculine titles can trigger unconscious biases, reinforcing stereotyped beliefs toward gendered roles, as well as influencing the actual success of women in such roles.[36] Along the same lines, the generic use of masculine forms (e.g., mankind) has been shown to potentially impact our perception of "man" as the conceptually generic, default human prototype.[37,38] To foster greater inclusivity, gender-neutral forms that avoid undue gendered mentions have been proposed (e.g., firefighter instead of fireman or singular "they"), and have been applied in more formal contexts, with dedicated guidelines being published by international institutions such as the European Parliament and in APA Style Manuals.

On the one hand, such neutral strategies aim to equally elicit all gender identities and prevent misgendering—i.e., the use of gendered language that does not reflect individuals' identity,[39,40] such as addressing someone with the wrong pronouns—by avoiding any specific gender assumptions.[41] On the other hand, to enhance self-expression and the visibility of identities outside the gender binary, innovative solutions have been emerging such as neopronouns[42,43] (e.g., en: xe) or neomorphemes[44–47] in languages with gendered morphology (e.g., it -ə/-ɜ, es -e/-es).

In (machine) translation scenarios, the proper handling of gender is further complicated by differences in how gender is encoded and expressed across languages. For example, an isolated sentence such as "The professor helped the kid" is gender ambiguous, whereas in Spanish it could map to several inflections—i.e., profesor/profesora/profesore and niño/niña/niñe. Although such ambiguity has been shown to be particularly prone to result in default masculine translations, MT can also struggle when explicit gender information is available.[48,49] Unequal representation in language can also be subtle, for instance by associating feminine forms with lower-prestige occupations only (e.g., it: E′ una professoressa F vs. E′ un professore M → She is a teacher vs. He is a professor).

In light of the above, it becomes clear how gender in language is a sensitive, value-laden feature, used to negotiate our identities, as well as capable of influencing others' views and assumptions. These challenges underscore how translating gender can inadvertently cause downstream harms. Bearing this in mind, we now move on to our literature review.

REVIEW OF GENDER BIAS IN AUTOMATIC TRANSLATION

To take stock of what has happened since the onset of research on gender bias in MT, we conduct a comprehensive review. We first describe our methodology and then move to our findings.

Review method

ACL anthology search

For a systematic review of prior work, we followed the PRISMA 2020 checklist[50] and queried the ACL Anthology (https://aclanthology.org/). We chose the ACL Anthology because it represents the primary database in the MT/NLP field, currently hosting over 100,000 papers. Besides, unlike searches based on arXiv (https://arxiv.org/) and Google Scholar (https://scholar.google.com/), the ACL Anthology allows us to retrieve only published and peer-reviewed works. To verify other potential relevant sources, we also searched the ACM FAccT proceedings (https://dl.acm.org/conference/facct/proceedings), but this query only returns one paper[51] on the topic of gender (bias) in automatic translation. To reduce the collection of noisy instances, our queries only applied to titles and abstracts. The searches were last run on 11 December 2024, and returned 308 unique articles published between 2014 and 2024. The selection of the eligible papers to review was carried out manually and based on the following criteria. We retained studies that (1) primarily focus on the automatic translation task, regardless of the underlying technology (e.g., SMT, LLMs) and for any modality beside text-to-text; (2) focus on gender translation of human entities only (i.e., unlike gender classes of inanimate nouns[52]), including papers on gender fairness and inclusivity. Accordingly, we discarded all unrelated papers that refer to, e.g., inductive bias, bias length, or translation that are not related to MT. As summarized in Table 1, this led to the selection of 133 eligible papers, with the first eligible papers being published in the year 2016. Henceforth referred to as "in scope," we rely on these papers to carry out fine-grained annotations and take stock of the field.

Annotation of in-scope papers

The in-scope papers were reviewed and annotated by two of the authors. Each annotator revised an equal number of works, which were fairly distributed based on their year of publication. For fine-grained analyses, the annotation taxonomy comprised 11 fields aimed at capturing both the conceptualization of bias (e.g., Does the paper engage with the social notion of bias?, How is gender conceptualized?) as well as major trends in the study of bias in automatic translation and their experimental design (e.g., What languages are involved in the study?, Does the paper present a mitigation strategy?). The annotations were based on detailed guidelines, which were jointly produced by the authors and progressively refined over several annotation rounds. To ensure the soundness of our review, all borderline instances were discussed and agreed upon by the two annotators. The complete taxonomy and annotation guidelines (Table S1), as well as a full list of reviewed papers, can be found in the supplemental information.

Review findings

We now report our findings, structured around the most salient observations we made.

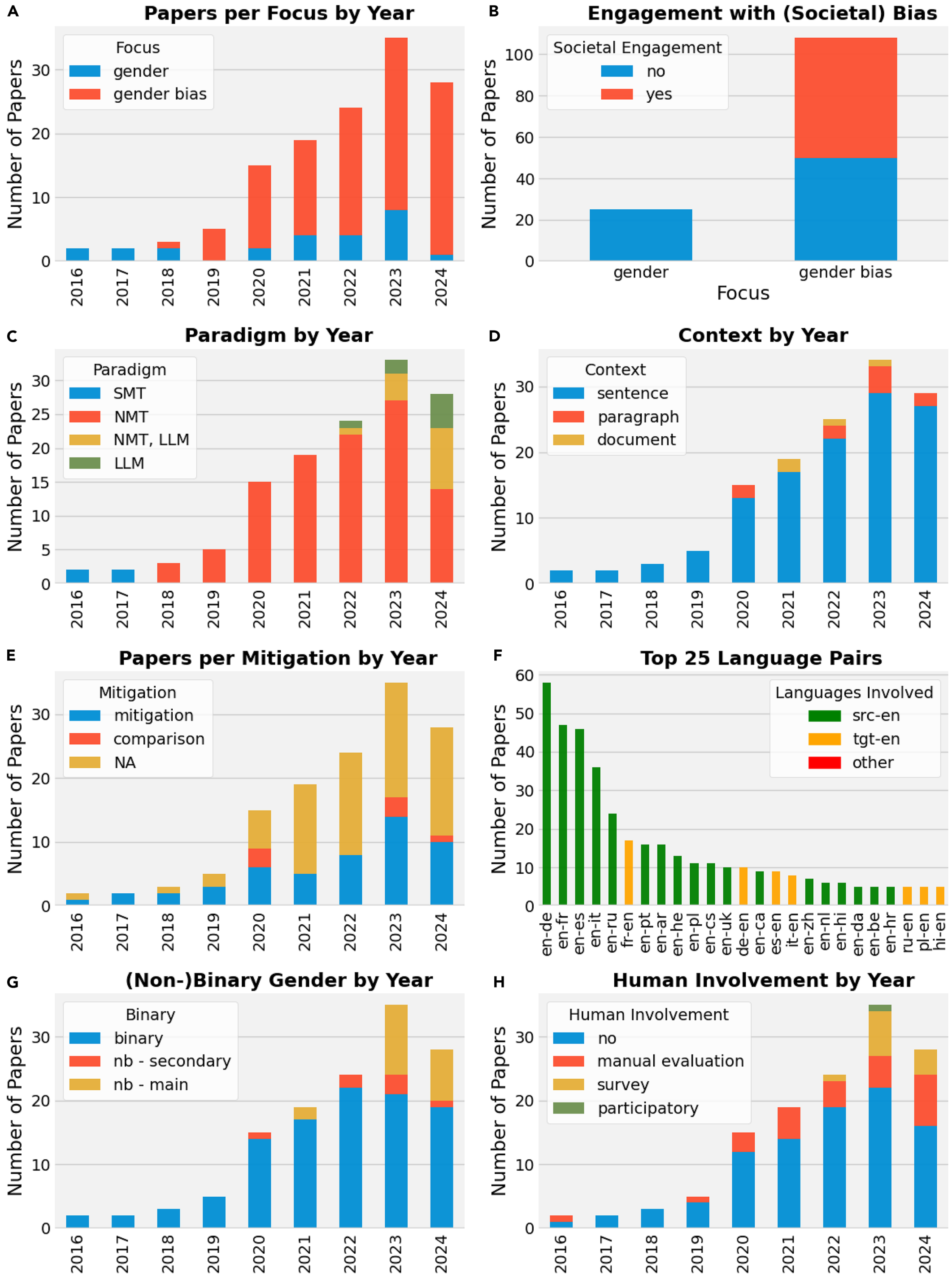

Figure 2. Key statistics of our literature review on gender bias in MT

Gender bias grew as an area of research

As illustrated in Figure 2A, the analysis of papers published on gender in automatic translation, starting from the first works identified as in scope in 2016, reveals a growth in publications up until 2023, with a particularly notable increase from 2019 to 2023. In general, this trend aligns with the broader expansion of research on gender in the area of NLP,[15] as shown by the first survey papers on the topic in NLP[53,54] and in machine translation

CellPress
OPEN ACCESS

(MT).[17] Notably, the initiation of the first workshop on Gender Bias in NLP[55] (GeBNLP) in 2019 and the Gender-Inclusive Translation Technologies (GITT) workshop[56] in 2023 contributed to the increased volume of research papers on this topic, with seven papers published between 2019 and 2020 and nine papers between 2022 and 2023. However, we note a small drop in 2024 (from 32 papers in 2023 to 28 the following year). Most likely—and as suggested by additional queries we ran on the ACL Anthology—this change of trend can be attributed to a decreasing focus on cross-lingual MT bias in favor of a growing emphasis on (mono-/multilingual) bias in LLMs across various generative tasks. In Figure 2A, it is also noted that, while most current research explicitly focuses on gender bias (108 papers), some works address gender translation for human entities without referring to the notion of bias. Given that dedicated research on bias and ethical aspects of NLP flourished later,[10] the first papers published between 2016 and 2018 focused on gender translation (see, for instance, other studies[57–60]). However, a subset of more recent works also focuses on gender as a variable, often including other linguistic phenomena, without making connections to the broader literature on bias. For example, Liu and Niehues[61] instantiate gender and formality as “desired attributes” to be controlled in cross-lingual transfer.

Bias without social engagement

Despite the growth of interest in the topic, we attest that existing research still often disregards the social component and causes of bias. As shown in Figure 2B, we find that even among papers that explicitly refer to gender bias as a phenomenon, only 58 openly recognize the societal and ethical component of bias by discussing its potential for harms on already disadvantaged groups. Beside a few exceptions,[62] engagement with the broader literature on gender, language, and inequalities is overall extremely rare, as previously attested by Blodgett et al.[12] In fact, 50 papers on gender bias out of 108 approach the issue as a data-driven technical problem or purely cross-linguistic problem, by overlooking the context in which bias manifests itself and its downstream effects.

Gender bias concerns all MT paradigms

If, on the one hand, a linearly increasing engagement with the ethical and societal aspects of bias is not found across publications, on the other hand research on gender in MT keeps pace with the latest technological advancements. As a matter of fact, as shown in Figure 2C, gender (bias) has been researched across all of the latest MT paradigms—SMT (four papers all from 2016 or 2017), NMT (119 papers)—as well as with the most recent LLMS (22 papers at least including LLMs). We observe the first indication of a shift toward LLMs in 2022.[63,64] By 2023, they have gained some traction with six out of 33 papers (roughly 18%) including LLMs. In 2024, LLM-based solutions become a key paradigm with half of the papers (14 out of 28) working on LLMs. Of these, nine compare or incorporate[65] NMT with LLM approaches and five focus exclusively on LLM-based methods. Regardless of the paradigm, there is an overall consensus that gender bias remains a critical challenge for machine translation technology, with both proprietary and open LLMs still reinforcing stereotypes and defaulting to masculine forms,[66–68] which is in line with the broader literature.[69]

Text to text is the dominating modality

Most studies on gender in MT exclusively focus on the TEXT modality (123 papers). As an exception in the realm of audio and visual translation, we attest seven works in speech translation (ST)[70–76] and one on image-guided translation.[77] Indeed, this lack of research on different modalities within the field was already raised by Savoldi et al.,[17] and still stands unaddressed. It is also worth noting that, while research on gender in ST is limited, many works on gender in MT for TEXT—including the earliest ones—focus on *spoken* language translation. This is likely due to the frequency of ambiguous gender references to speakers (e.g., I am a student),[78] making their correct gender realization—i.e., avoiding misgendering—a critical, long-standing task.

Most work is on sentence-level translation

Figure 2D shows that, despite attempts to move beyond small units of text, research in the field of automatic translation is still mostly carried out at the sentence level[79] also for the study of gender bias (118 papers). Studies working at the paragraph—i.e., up to 10 sentences[80–89]—or document level—i.e., considering more than 10 sentences[90–93]—are in fact exceptional. However, gender often requires additional context; e.g., gender information might have been specified outside the context span of a single sentence, and context helps disambiguate if rendering gender is relevant at all (e.g., *chairman* generically used instead of *chair*, or to refer to a specific man). Furthermore, one of the reviewed papers[94] looked specifically at the need for context for the evaluation of MT output. From their analysis of translations from English into Serbian and Portuguese, gender is identified as one of the most common issues hindering the translation of isolated sentences across different domains (reviews, subtitles, and literature).

Many mitigation proposals but no clear winner

The development of mitigating strategies to counter bias is central to much research in MT. Figure 2E shows that nearly half (51) of the reviewed papers propose a dedicated mitigation strategy. A minor trend (eight papers) involves comparison across architectures[71,77,81,87,88,90,95,96]—e.g., proposing document-level MT for resolving inter-sentential gender ambiguity—or modalities—e.g., suggesting that image-guided translation can infer a referent’s gender from visual input and translate accordingly (see also section discussion). Most other papers focus on analyses or the creation of novel benchmarks and bias metrics—see Table 2 for a summary of popular MT bias benchmarks. Technically and conceptually, the proposed approaches to mitigate bias vary. Some involve dedicated training (10) or fine-tuning (nine) on curated data—e.g., countering stereotypical associations[97,98]—or word embeddings debiasing to remove such associations.[99–101] Inference-time approaches (seven) are used when an oracle is available, i.e., when the system’s behavior regarding gender is known in advance, as in speaker gender translation.[102,103] For ambiguous gender translation, a growing post-processing approach (10) involves using a rewriter to convert text from masculine to feminine (or vice versa)[104,105] or from gendered to gender-neutral language.[106–108] In recent LLM studies, various prompts control the desired gender form—masculine, feminine, or neutral[65,68,109,110]—using dedicated instructions and demonstrations. Overall, the proposed solutions

Table 2. Popular MT gender bias benchmarks

Benchmark	Year	Languages[a]	Data type	Domain	Evaluated words	Features	Context
Equity evaluation corpus[197]	2018	ko-enˆ	artificial	template	pronouns	ambiguous	sentence
WinoMT[198]	2019	en-{ar, cs, de, en, fr, he, it, pl, ruˆ}	artificial	template	occupations	Unambiguous[b], stereotype annotation	sentence
Occupation test set[200]	2019	en-es	artificial	template	occupations	unambiguous; stereotype annotation	sentence
Arabic parallel gender corpus[201,202]	2019	en-ar	natural	open subtitles	any gendered word	ambiguous (speaker and listener)	sentence
Must-SHE[203]	2020	en-{it, fr, es}	natural	Ted Talks	any gendered word	ambiguous (speaker); unambiguous	sentence
SimpleGen[204]	2021	en-{es, de}	artificial	template	occupations	unambiguous, stereotype annotation	sentence
BUG[205]	2021	en	natural	Wikipedia, medical	occupations	unambiguous, stereotype annotation	sentence
MT-GenEval[83]	2023	en-{ar, fr, de, hi, it, es, pt, ru}	natural	Wikipedia	any gendered word	unambiguous	sentence; context
GeNTE[119]	2023	en-it	natural	Europarl	any gendered word	ambiguous (gender neutral), unambiguous	sentence
Multilingual holistic bias[206]	2023	en-* and *-en (50 language pairs)	artificial	template	noun + descriptor	ambiguous	sentence
MiTTenS[67]	2024	en ↔ {ar, zh, fr, de, hi, it, ja, pt, ru, es, fi, id, pl, te, tr, th, am, as, bn, cs, fa, mai, or, bho, ln, lg}	artificial, natural	multi	pronouns	unambiguous	sentence; context

[a]For languages marked with ˆ, evaluations are conducted without reference translations, relying instead on methods like morphological analysis of target words.
[b]WinoMT is intended to be used for unambiguous translations, though it relies on Winograd-like structures that are actually open to multiple interpretations. The WinoMT dataset adaptation by Saunders and Byrne[199] additionally analyzes translations of ambiguous occupations.

CellPress
OPEN ACCESS

Table 3. Example sentences intersecting gender and sexual orientation

	EN	The girl met with her partner	The boy met with his partner
	IT	La ragazza si è incontrata con *il suo partner*$^{(m)}$.	! Il ragazzo si è incontrato con *il suo partner*$^{(m)}$.
DeepL	DE	Das Mädchen traf sich mit ihr*em partner*$^{(m)}$.	! Der Junge traf sich mit sein*em partner*$^{(m)}$.
	FR	La jeune fille a rencontré *son partenaire*$^{(m)}$.	! Le garçon a rencontré *son partenaire*$^{(m)}$.
	IT	La ragazza ha incontrato *il suo compagno*$^{(m)}$.	Il ragazzo incontrò *la sua compagna*$^{(f)}$.
Google T	DE	Das Mädchen traf sich mit ihr*em partner*$^{(m)}$.	Der Junge traf sich mit seiner *Partnerin*$^{(f)}$.
	FR	La fille a rencontré *son partenaire*$^{(m)}$.	! Le garçon a rencontré *son partenaire*$^{(m)}$.
	IT	La ragazza ha incontrato *il suo partner*$^{(m)}$.	! Il ragazzo ha incontrato *il suo partner*$^{(m)}$.
ChatGPT	DE	Das Mädchen traf sich mit ihr*em partner*$^{(m)}$.	! Der Junge traf sich mit sein*em partner*$^{(m)}$.
	FR	La fille a rencontré *son partenaire*$^{(m)}$.	! Le garçon a rencontré *son partenaire*$^{(m)}$.
	IT	La ragazza ha incontrato *il suo partner*$^{(m)}$.	! Il ragazzo ha incontrato *il suo partner*$^{(m)}$.
Gemini	DE	Das Mädchen traf sich mit ihr*em partner*$^{(m)}$.	! Der Junge traf sich mit sein*em partner*$^{(m)}$.
	FR	La fille a rencontré *son partenaire*$^{(m)}$.	! Le garçon a rencontré *son partenaire*$^{(m)}$.

We test if current models—DeepL, Google Translate, ChatGPT (GPT-4), Gemini Advanced—reproduce heteronormative views representing couples between people of different genders. We highlight the translation of the ambiguous source word "partner" as either masculine (m) or feminine (f) in the target language. Results show that template sentences with the entity "the girl" always trigger a heteronormative behavior, whereas template sentences with the entity "the boy" do not, and mostly represent "partner" as masculine—indicated with (!). This might be due to the tendency toward a masculine default that overrides the heteronormative behavior. Prompt used for LLMs: Please translate the following English sentence into [language]: " …"; queries done August 11, 2024. IT, Italian; DE, German; FR, French.

vary in how they approach different components of the translation pipeline.

More importantly, they address specific challenges related to gender translation scenarios and different conceptualizations of bias in a modular way, by testing each approach on focused benchmarks, or they are intended for a limited number of languages.

Few, highly resourced languages

Research on gender bias has a long tail of rarely studied languages. The most investigated language pair is en-de (included in 58 studies), followed by en-fr (47) and en-es (46). Figure 2F shows that a handful of language pairs dominate, and outside the top 25 we only register a count of 1–2 for all remaining language pairs. We attest that most papers (1) focus on major and most well-supported Indo-European language pairs and (2) involve English, mostly as a source language (i.e., src-en) for translation into grammatical gender languages but also as a target language (i.e., tgt-en). What remains is a long tail of minor investigations.

Limited, binary treatment of gender

The majority of papers treat gender as a binary category (105 out of 133) and most of those do not discuss what conceptualizations of gender they employ—an issue that is also observed in the wider NLP literature.[111] Often, results are disaggregated and discussed for a male/female or men/women dichotomy, even in cases where terminology from linguistics (masculine/feminine) would be more appropriate. In the last few years, however, as shown in Figure 2G, there has been an increasing trend toward papers that are not restricted to a binary, reductionist vision of gender. Broader analyses and studies on gender inclusivity and fairness, also accounting for non-binary identities and gender-inclusive language, have started out as minor sections of papers (i.e., n.b., secondary),[63,97,109,112–115] but are increasingly the main focus (i.e., n.b., main)[68,93,96,107,108,116–120] *inter alia*. Overall, we thus attest to a growing awareness of non-binary identities and studies of linguistic expressions that are inclusive and representative for them, too.

There is hardly any intersectional work

As already highlighted in legal and social science theory, discrimination can arise from the intersection of multiple identity categories[121] that are not additive and cannot always be detected in isolation.[122,123] From our analysis, it appears that only five papers account for the interaction of gender attributes with other sociodemographic axes.[62,124–126] Sometimes, papers account for multiple identity aspects, but not in intersection.[127] One exception is Stewart and Mihalcea,[126] who intersected gender and sexual orientation to see if heteronormative views are reproduced by translation systems. In Table 3, inspired by that work, we show that such heteronormative bias still exists in the most recent systems.

Bias without people

We attest a severe lack of human engagement in the study of gender in MT, in line with recent findings by Savoldi et al.[14] In fact, only 40 works rely on human evaluation to measure bias, though in a different capacity, which we distinguish into three conceptual categories in Figure 2H. In 27 papers, we find that people—often expert linguists (e.g., Vanmassenhove et al.,[116] Soler Uguet et al[128])—are involved in manual evaluation. This serves to either ensure correlation with bias metrics (e.g., Kocmi et al.[129]) or to gain qualitative insights that defy automatic approaches.[130] While indeed valuable, such analyses are a support for structured, often annotation-based model-centric evaluations—i.e., that inform and quantify models' behavior. Differently, the 12 papers in the "survey" category focus on the feedback and experiences of potentially impacted groups of users (e.g., Piergentili et al.[119]). For instance, they do so to grasp user preference

Table 4. Translation examples between Italian and German, two grammatical gender languages

	IT	Volevo essere *un ballerino sexy*$^{(m)}$ in uno di quei video musicali fin da quando ero *ragazzino*$^{(m)}$
		I wanted to be *a sexy dancer* in one of those music videos since I was a *boy*
DeepL	DE	! seit ich ein Kind war, wollte ich *eine sexy Tänzerin*$^{(f)}$ in einem dieser Musikvideos sein
Google T.	DE	! schon als Kind wollte ich *eine sexy Tänzerin*$^{(f)}$ in einem dieser Musikvideos sein
ChatGPT	DE	ich wollte schon als *Junge ein sexy Tänzer*$^{(m)}$ in einem dieser Musikvideos sein
Gemini	DE	ich wollte schon als *Junge ein sexy Tänzer*$^{(m)}$ in einem dieser Musikvideos sein

Here, gender translation is unambiguous but anti-stereotypical. We highlighted masculine (m) and feminine (f) expressions in both source and output sentences. DeepL and Google Translate render "un ballerino sexy" (a sexy dancer) as feminine, despite the referent being explicitly masculine in the source sentence. ChatGPT (GPT-4) and Gemini Advanced are able to provide a correct masculine translation, using the following prompt: Please translate the following Italian sentence into German: " …"; queries done August 11, 2024.

in how models should handle the translation of neopronouns from English—e.g., ze or xe[120]—or to understand the potential trade-off between overall quality and inclusivity goals.[108] Interestingly, all survey works focus on non-binary linguistic strategies beside feminine/masculine ones. Finally, the study by Gromann et al.[131] recounts participatory action research, where a community-led approach with different stakeholders informs the state and potential direction for gender-fair MT.

DISCUSSION

In the previous section, we reviewed a decade of research on gender and bias to assess the progress made in the field. Our findings indicate that research has been dynamic with a relatively steady growing interest in addressing bias. However, the critical question remains: is gender bias *fixed*? In the following discussion, we will explore this question and, considering the current technological landscape along with the rise of advanced language models, outline the opportunities and challenges that lie ahead, building on the foundation of the past decade's research.

Can bias be fixed?

Let us circle back to the introductory quote and reflect on the optimistic assertion that "We can fix that!" Although the early enthusiasm in bias research suggested a quick resolution, more than a decade later, a definitive solution remains elusive. The continued growth in this field (cf. Figure 1), as also shown by the emergence of dedicated venues on the topic (e.g., GITT and GeBNLB), indicates ongoing interest and commitment. However, it also suggests that bias is neither resolved nor fully understood. Our review of 133 papers reveals promising trends, such as the rise of studies addressing the inclusion of non-binary identities and linguistic expression. Yet, we identified several gaps, both in practical experimental approaches and broader normative and theoretical reflections, that impact how bias is conceptualized.

Experimentally, while there has been a notable number of proposed mitigation strategies and analyses, most efforts have been English-centric and focused on (predominantly sentence-level) text-to-text systems. This narrow scope limits their applicability across different languages, cultures, and technological contexts, and risks overlooking cultural, linguistic, and societal differences. For example, by operationalizing Western stereotypes—often based on US occupational statistics[132]—and by focusing on pronouns (see also Table 2) we may neglect how other languages express gender or introduce inclusive linguistic innovations (e.g., in languages with grammatical gender, high-prestige feminine occupational nouns may still be lacking,[133] and neutral, degendering strategies may face institutional push-back[134]). Then, multimodal MT—when MT systems rely on audio or images as input signals—can lead to undue reductionist, binary gender classifications,[135,136] by associating gender with voice pitch,[137] clothing,[138] or physical characteristics. Moreover, the study of bias has lacked grounding in real-world scenarios, particularly concerning the people most impacted and the various axes of discrimination that may arise. Mitigation strategies and benchmarks have often addressed artificial, template-based sentences (see Table 2), which hardly reflect how bias might manifest itself in realistic MT usage, or how it can bring harms and inequity. As a case in point, generating multiple alternative translations for ambiguous gender—now integrated into online tools like Google Translate and DeepL for simple sentences—can create user interface challenges. The study by Vanmassenhove and Monti[139] shows that more realistic sentences with multiple referents can result in over 10 possible translation alternatives. Moreover, so far, these solutions only provide masculine and feminine options, thus excluding identities that do not fit binary gender. This points to a challenge to handle more complex inputs and an opportunity to be more inclusive with a new kind of user interface that can handle richer disambiguation scenarios.

Finally, much research has overlooked the intrinsically social components and consequences of bias, treating it as a statistical, technical problem.[140] This has fostered technological optimism that "it can be fixed." However, there is no consensus on what constitutes an *unbiased* system or whether such a goal is even attainable. Bias is a sociotechnical problem, rooted in pre-existing normative discrimination and asymmetries,[17,141] and is contextually and evolutionarily dynamic.[142] Therefore, its study cannot be fully standardized or static,[143] and thus the question itself of whether it can be fixed could be misguided. As we have seen, bias is complex, and arguably no single, definitive technical intervention is likely to meaningfully address the issues it engenders.

Aware that mitigating gender bias is a moving target, it is essential to continuously account for evolving social, linguistic, contextual, and technological changes to refine our approaches to minimize harm to marginalized and disadvantaged gender groups. Bearing this in mind, and in light of the gaps and trends elicited from a decade of research on gender bias in MT, we will now rely on our findings to navigate the current landscape—one in which LLMs are increasingly popular—and to outline future paths.

Opportunities and challenges

The emergence of LLMs prompts us to revisit our findings and how they relate to the current technological shift. Building on the past research foundation, we explore the opportunities that these advancements currently present, as well as the challenges, for the years ahead.

Multilinguality and multiculturalism

MT gender bias research so far has exhibited a narrow language focus; i.e., it predominantly centers on English, often as the source language, often with another Western language as the target. This creates a "winner-takes-all" scenario where well-supported languages receive the most attention in terms of solutions, benchmarks, and monitoring, leading to a significant risk of perpetuating anglocentric biases, an issue that is well documented also more broadly in NLP.[144,145] Moreover, there is a misconception that gender bias does not concern languages with similar grammatical structure, presumably because it poses to no challenges in human translation. Table 4 shows an example of Italian-German translation that highlights how gender bias can indeed be present in such cases. Now, the emergence of highly multilingual models could enable research across a diverse set of languages without the need for *ad hoc* models. Still, many popular LLMs that are used multilingually perform best in English[146–150] also due to the pertaining mixtures (e.g., Llama 3 reportedly contains around 95% of English data, and GTP-3 around 92.6%), and commercial models such as GPT-3.5[151,152] offer lower-quality service for low-resource languages at potentially higher costs.[147,150] Crucially, multilinguality does not guarantee *multiculturalism*.[153] Large multilingual models can tacitly encode Western, educated, industrialized, rich, and democratic (WEIRD) perspectives, leading to cultural homogenization and the leakage of cultural values and stereotypes across languages.[154–156] The study of gender bias requires sensitivity to each cultural context and language community. For example, Luthra and Nijman[157] discuss the difficulties of accurately conveying gender information in historical archives of the Dutch East India Company (VOC), and Talat et al.[153] discuss how the US notion that parental leave is primarily for mothers may be irrelevant in Sweden, where parental leave is more equally divided between parents. Gender itself is experienced differently across cultures,[158] and it is much richer than its folk understanding suggests; the Hijra community in South Asia,[159,160] and two-spirit people—an umbrella term used by Indigenous North Americans[161]—are just two examples of that. Thus, while the rise of LLMs might support broadening the multilingual reach of bias research, it also highlights the need for nuanced approaches and frameworks that do not merely expand linguistic coverage but rather also account for the diversity of different language communities and the potential misalignment between languages, biases, and values within current models.

Bias is not unidimensional and neither is language (modeling)

Gender bias research in MT has predominantly focused on fairness within the textual, written modality, often overlooking the inherently multimodal nature of communication, which involves gestures, speech, and visual cues. With the rise of multimodal systems, which integrate text and vision (e.g., VisionLLM,[162] LLaVA[163]) or speech (e.g., Whisper,[164] SeamlessM4T[165]), it is foreseeable that MT will further leverage non-textual information.[166–168] If this shift enables richer, more contextually grounded translations, it also demands revisiting how bias has been formalized, beyond just the disambiguation and rendering of (written) linguistic gendered forms. As previously discussed in the few existing cross-lingual works on the topic[70,71,76,77]—and as already shown in mono/multilingual settings[169,170]—multimodality introduces new challenges, as it must account for other expressions of gender and identity related to voice or appearance. Moreover, the interaction of different aspects and modalities requires a more multifaceted study of bias, which in itself can intersect with other axes of discrimination and inequality. As multimodal systems become more prevalent, the study of bias must also advance to address their specific complexities, taking into account how dialect, skin color, speech disfluencies, and other factors might influence translation performance across sociodemographic groups.

User-centric, realistic assessment of bias

Although the study of bias and equitable language technology has been stressed as intrinsically human-centered—focused on understanding what behaviors are harmful, how they manifest, and who is affected[12]—in our review we found a notable lack of direct human engagement in MT gender bias research. This gap is problematic as it is essential to consider the actual experiences and needs of users to address real-world harms and how they might arise.[171] As LLMs are employed in user-facing chatbots (e.g., ChatGPT, Gemini Advanced), the importance of understanding how users interact with them grows.[172] So far, the adopted experimental scenarios and benchmarks vastly rely on decontextualized, often artificial, sentence-level tests that fail to reflect the complexity of real-world usage and human interaction.[14,173,174] These tests also do not reflect more recent LLMs capabilities. For instance, while typical NMT models may process up to 512 tokens (e.g., NLLB), recent LLMs can handle up to 2 million tokens (e.g., Gemini 1.5 Pro), allowing them to pick up on gender cues beyond single sentences, grant a gender translation advantage that is missed with sentence-level evaluations (see also Gautam et al.[175] for context-based performance on pronouns resolution). Moreover, given the general-purpose nature of many LLMs, automatic translation may be just one component of a more complex task. For example, users might request a translation, summarization, and tone adjustment all at the same time. This requires a rethinking of *verbatim* cross-lingual transfer as the sole mode in which bias might manifest itself and be identified in MT-related scenarios. Future research is thus needed to ensure that gender bias investigations are grounded in users' lived experiences and keep up with the ways in which technologies are actually employed. Engaging users directly with participatory methods can provide essential insights into how bias affects them. Also, human-computer interaction (HCI) approaches can help bridge the gap between technical assessments and real-world applications[176–178] and inspire the creation of realistic assessments that are not only technically sound but meaningful for MT users.

Linguistic innovations and data curation

As societal awareness of non-binary identities grows, recent research in MT has begun addressing the inclusion and recognition of emerging non-binary linguistic expressions. However, even strong online NMT models—i.e., Amazon Translate, Bing, Google Translate, and DeepL—cannot handle the translation of

neopronouns[120] or generate gender-inclusive translations,[68,119] apart from rare exceptions.[179] LLMs have already brought new opportunities in this area, particularly due to their in-context learning capabilities,[151] which grant them greater versatility in controlling various output attributes compared to traditional NMT models.[180–184] Recent studies have demonstrated that LLMs can translate using Italian neomorphemes and gender neutralizations with promising results when they are provided with minimal instructions and a few demonstrations.[68,185] However, despite these advancements, the ability to adapt varies significantly across different LLMs,[185] and there remains a notable gap in effectively handling binary versus non-binary forms.[186] The training of LLMs on data created in the (distant) past limits this flexibility and makes it harder to adequately represent and reproduce contemporary linguistic expressions. Indeed, as investigated by Gaido et al.[72] and Ovalle et al.,[187] the poor representation of feminine forms or neopronouns in training data leads to their over-segmentation by popular tokenization approaches, resulting in more fragmented tokens and worse performance. Data can actively be curated and created to explicitly ensure the representation of marginalized identities[188] and steer desirable model behaviors with dedicated training[189] or fine-tuning.[190] Studying the connection between data and model behavior is therefore an important area of research, one that is made difficult because of the sheer size of pre-training datasets and their often undisclosed nature. A fundamental question remains: can data and model interventions fully capture the diverse preferences of a broad range of users and their idea of gender fairness and harm reduction?[191] This brings us to our final point.

Personalization

The variation in how gender bias is conceptualized and operationalized discussed so far reflects the complexity of defining what constitutes gender bias and harmful behavior in MT. Besides, even if we agree on certain harmful behaviors—such as reinforcing negative stereotypes (e.g., assuming doctors are men and nurses are women) or making undue gender inferences that lead to misgendering—the idea of what a fair solution looks like can still differ at a micro level, with users legitimately having varied preferences.[68,191] This is especially true for cases of ambiguity. While research on this topic in MT is limited (see user-centric paragraph), the survey by Lauscher et al.[120] reveals that individuals disagree on how English neopronouns should be translated, mirroring the needs for diversified translation policy. This paves the way for personalization, an area of research that—while not new—has gained particular attention with the rise of LLMs (see also Kirk et al.[192]). Despite being increasingly general purpose and multitask, there is a growing demand for LLMs that respond to the specific requests of individual users. Some opportunities for personalization have already emerged. For example, users themselves can craft specific prompts to achieve their desired outputs, though this admittedly requires some skill. Moreover, as Anthis et al.[193] notes, LLM service providers are increasingly customizing systems for individual users, such as by incorporating past chat history into the current output and allowing users to personalize their interactions further. On the one hand, personalizing LLMs through micro-level preference learning may lead to models that are better aligned with each user and the proper representation of their gender identities. On the other hand, defining the boundaries of an ethically and socially acceptable level of personalization poses significant normative challenges.[192,194] In particular, this level of customizations poses ethical and privacy concerns, especially if the model handles sensitive gender data or encourages users to disclose personal information. Thus, further works in this area are required to weigh and ensure responsible and transparent deployment, exploring privacy-preserving mechanisms[195] and compliance with regulatory frameworks.[196]

CONCLUSIONS

Gender bias in MT has been a concern in the MT community for over a decade, one in which societal, linguistic, and technological shifts have been prominent. The earliest concerns and calls to action, underscoring the need to consider gender in scientific research and translation technology, came in 2014, bringing together academia and industry. At the time, a quick resolution was expected—"we can *fix* that!" Starting from this assumption, this paper takes stock of more than 10 years of research on gender bias and relies on the past to reflect on the present and speculate on the future of gender bias research.

With this aim, we first carry out a comprehensive review of over 100 papers queried from the ACL Anthology. Based on a detailed analysis that accounts for the field's progress along several conceptual and experimental aspects, we identify key trends and advancements made by the community but also persistent gaps. We find that the MT community has vastly engaged with the issue, leading to growing research on the topic with novel methods, analyses, resources, and dedicated initiatives, such as workshops that bridge the multidisciplinary MT and translation studies field. Also, recent trends show an increasing engagement to ensure the recognition of non-binary gender identities and explorations into user needs. Concurrently, most work was done with text-to-text sentence-level systems and adopted an anglocentric approach. We also see that gender bias, which had been an issue since the days of SMT, continued to plague us during the deep-learning revolution that got us NMT and is still an issue in the age of LLMs.

We argue that bias—as a multifaceted and contextual sociotechnical problem—cannot be resolved with a single, definitive technical fix. Rather, it requires continuously accounting for evolving social, linguistic, contextual, and technological changes so as to refine our approaches and real-word understanding of the issue to minimize harms. As such, building on the lessons learned from our research's review, we reflect on the current landscape of gender bias in MT and in the context of the growing popularity of LLMs. While these models offer new opportunities (e.g., multimodal and multilingual solutions, personalization, in-context learning, and multitask capabilities), we discuss how they also give rise to both novel as well as persistent challenges inherited from the past decade. As research on gender bias in MT advances by allowing for a deeper understanding of the problem's complexity, we introduce our review and discussions as a scaffolding for future research: as a path for the attested sustained effort to mitigate the societal impacts of MT on marginalized and disadvantaged gender groups.

Limitations of the study

Our review only considers peer-reviewed papers in the ACL Anthology. However, the ACL Anthology is the home of the vast

CellPress
OPEN ACCESS

majority of peer-reviewed papers on the topic of MT, including all proceedings of the Conference (formerly, workshop) on Machine Translation (WMT), as well as conferences such as ACL, EMNLP, NAACL, and EACL, with MT and bias tracks. As such, it represents the main historical reference point in the field.

All authors of this paper are from European backgrounds and identify as White. As a result, our understanding of linguistic contexts and notions of bias is inevitably shaped by this perspective. While we have actively advocated for broader shifts away from anglocentric and WEIRD viewpoints in the discussion, we acknowledge that our own positionality may have influenced the ways in which we engage with these topics. In particular, our selection of examples and framing of bias do not fully capture the lived experiences and linguistic realities of underrepresented languages and cultural contexts.

ACKNOWLEDGMENTS

The work by B.S. is funded by the PNRR project FAIR - Future AI Research (PE00000013) under the NRRP MUR program funded by the NextGenerationEU. The work by E.V. is part of a GROWTH project funded by the Digital Sciences for Society program of Tilburg University (TiU). We would also like to thank Sonja Siebeneicher (TiU) for their efforts and contributions during the initial stages of the GROWTH project. The work by L.B. is funded by the European Union's Horizon research and innovation programme under grant agreement no. 101135798, project Meetween (My Personal AI Mediator for Virtual MEETtings BetWEEN People).

AUTHOR CONTRIBUTIONS

Conceptualization, B.S., J.B., and E.V.; data curation (paper search and annotation), B.S. and J.B.; investigation, B.S. and J.B.; methodology (data annotation design), B.S. and J.B.; visualization, B.S. and J.B.; funding acquisition, E. V.; writing – original draft, B.S, J.B., and E.V.; writing – review & editing, B.S., J. B., E.V., and L.B.

DECLARATION OF INTERESTS

The authors declare no competing interests.

DECLARATION OF GENERATIVE AI AND AI-ASSISTED TECHNOLOGIES IN THE WRITING PROCESS

During the preparation of this work, the authors used Grammarly and QuillBot in order to improve readability and language. After using this tool/service, the authors reviewed and edited the content as needed and take full responsibility for the content of the published article.

SUPPLEMENTAL INFORMATION

Supplemental information can be found online at https://doi.org/10.1016/j.patter.2025.101257.

REFERENCES

1. Schiebinger, L. (2014). Scientific research must take gender into account. Nature *507*, 9. https://doi.org/10.1038/507009a.
2. Butler, J. (2024). Who's Afraid of Gender? (Macmillan Publishers).
3. Conrod, K. (2019). Pronouns Raising and Emerging (Ph.d. dissertation University of Washington).
4. Conrod, K. (2020). Pronouns and gender in language. The Oxford Handbook of Language and Sexuality (Oxford University Press). https://doi.org/10.1093/oxfordhb/9780190212926.013.63.
5. Koehn, P., Och, F.J., and Marcu, D. (2003). Statistical phrase-based translation. In Proceedings of the 2003 Conference of the North American Chapter of the Association for Computational Linguistics on Human Language Technology - Volume 1. NAACL '03, pp. 48–54. https://doi.org/10.3115/1073445.1073462.
6. Vaswani, A., Shazeer, N., Parmar, N., Uszkoreit, J., Jones, L., Gomez, A. N., Kaiser, L., and Polosukhin, I. (2017). Attention is all you need. 30. In Advances in Neural Information Processing Systems, pp. 5998–6008.
7. Radford, A., Narasimhan, K., Salimans, T., and Sutskever, I. (2018). Improving Language Understanding by Generative Pre-training (OpenAI Blog).
8. Anil, R., Dai, A.M., Firat, O., Johnson, M., Lepikhin, D., Passos, A., Shakeri, S., Taropa, E., Bailey, P., and Chen, Z.; others (2023). Palm 2 technical report. Preprint at arXiv. https://doi.org/10.48550/arXiv.2305.10403.
9. Dubey, A., Jauhri, A., Pandey, A., Kadian, A., Al-Dahle, A., Letman, A., Mathur, A., Schelten, A., Yang, A., Fan, A., et al. (2024). The Llama 3 Herd of Models. Preprint at arXiv. https://doi.org/10.48550/arXiv.2407.21783.
10. Hovy, D., and Spruit, S.L. (2016). The social impact of natural language processing. Berlin, Germany. In Proceedings of the 54th Annual Meeting of the Association for Computational Linguistics (Volume 2: Short Papers), pp. 591–598. https://doi.org/10.18653/v1/P16-2096.
11. Crawford, K. (2017). The trouble with bias. In Keynote at Neural Information Processing Systems (NIPS), Long Beach, USA.
12. Blodgett, S.L., Barocas, S., Daumé III, H., and Wallach, H. (2020). Language (technology) is power: A critical survey of "bias" in NLP. In Proceedings of the 58th Annual Meeting of the Association for Computational Linguistics (Online), pp. 5454–5476. https://doi.org/10.18653/v1/2020.acl-main.485.
13. Dev, S., Monajatipoor, M., Ovalle, A., Subramonian, A., Phillips, J., and Chang, K.-W. (2021). Harms of gender exclusivity and challenges in non-binary representation in language technologies. In Proceedings of the 2021 Conference on Empirical Methods in Natural Language Processing. Online and Punta Cana, Dominican Republic, pp. 1968–1994. https://doi.org/10.18653/v1/2021.emnlp-main.150.
14. Savoldi, B., Papi, S., Negri, M., Guerberof-Arenas, A., and Bentivogli, L. (2024). What the harm? quantifying the tangible impact of gender bias in machine translation with a human-centered study. In Proceedings of the 2024 Conference on Empirical Methods in Natural Language Processing, pp. 18048–18076. https://doi.org/10.18653/v1/2024.emnlp-main.1002.
15. Stańczak, K., and Augenstein, I. (2021). A survey on gender bias in natural language processing. Preprint at arXiv. https://doi.org/10.48550/arXiv.2112.14168.
16. Costa-jussà, M.R. (2019). An analysis of gender bias studies in natural language processing. Nat. Mach. Intell. *1*, 495–496. https://doi.org/10.1038/s42256-019-0105-5.
17. Savoldi, B., Gaido, M., Bentivogli, L., Negri, M., and Turchi, M. (2021). Gender bias in machine translation. Trans. Assoc. Comput. Linguist. *9*, 845–874. https://doi.org/10.1162/tacl_a_00401.
18. Bojar, O., Chatterjee, R., Federmann, C., Haddow, B., Huck, M., Hokamp, C., Koehn, P., Logacheva, V., Monz, C., Negri, M., et al. (2015). Findings of the 2015 Workshop on Statistical Machine Translation. Lisbon, Portugal. In Proceedings of the Tenth Workshop on Statistical Machine Translation (WMT15), pp. 1–46. https://doi.org/10.18653/v1/W15-3001.
19. Bojar, O., Chatterjee, R., Federmann, C., Graham, Y., Haddow, B., Huck, M., Yepes, A.J., Koehn, P., Logacheva, V., Monz, C., et al. (2016). Findings of the 2016 Conference on Machine Translation (WMT16). In Proceedings of the First Conference on Machine Translation (WMT16) vol. 2. Berlin, Germany, pp. 131–198.
20. Bentivogli, L., Bisazza, A., Cettolo, M., and Federico, M. (2016). Neural versus Phrase-Based Machine Translation Quality: a Case Study. In Proceedings of the 2016 Conference on Empirical Methods in Natural Language Processing, EMNLP. Austin, Texas, USA, pp. 257–267. https://doi.org/10.18653/v1/D16-1025.
21. Sutskever, I., Vinyals, O., and Le, Q.V. (2014). Sequence to sequence learning with neural networks. In Proceedings of Advances in Neural

Information Processing Systems 27: Annual Conference on Neural Information Processing Systems. Montreal, Quebec, Canada, pp. 3104–3112.

22. Cho, K., van Merriënboer, B., Gülçehre, Ç., Bahdanau, D., Bougares, F., Schwenk, H., and Bengio, Y. (2014). Learning Phrase Representations using RNN Encoder–Decoder for Statistical Machine Translation. In Proceedings of Empirical Methods on Natural Language Processing (EMNLP). Doha, Qatar, pp. 1724–1734. https://doi.org/10.3115/v1/D14-1179.

23. Bahdanau, D., Cho, K., and Bengio, Y. (2014). Neural machine translation by jointly learning to align and translate. Preprint at arXiv. https://doi.org/10.48550/arXiv.1409.0473.

24. Xue, L., Constant, N., Roberts, A., Kale, M., Al-Rfou, R., Siddhant, A., Barua, A., and Raffel, C. (2021). mT5: A massively multilingual pre-trained text-to-text transformer. In Proceedings of the 2021 Conference of the North American Chapter of the Association for Computational Linguistics: Human Language Technologies, pp. 483–498. https://doi.org/10.18653/v1/2021.naacl-main.41.

25. Zhu, W., Liu, H., Dong, Q., Xu, J., Huang, S., Kong, L., Chen, J., and Li, L. (2024). Multilingual machine translation with large language models: Empirical results and analysis. Findings of the Association for Computational Linguistics: NAACL *2024*, 2765–2781. https://doi.org/10.18653/v1/2024.findings-naacl.176.

26. Richburg, A., and Carpuat, M. (2024). How multilingual are large language models fine-tuned for translation? In First Conference on Language Modeling (COLM).

27. Alves, D.M., Pombal, J., Guerreiro, N.M., Martins, P.H., Alves, J., Farajian, A., Peters, B., Rei, R., Fernandes, P., Agrawal, S., et al. (2024). Tower: An open multilingual large language model for translation-related tasks. In First Conference on Language Modeling (COLM).

28. Xu, H., Kim, Y.J., Sharaf, A., and Awadalla, H.H. (2024). A paradigm shift in machine translation: Boosting translation performance of large language models. Preprint at arXiv. https://doi.org/10.48550/arXiv.2309.11674.

29. Ackerman, L. (2019). Syntactic and cognitive issues in investigating gendered coreference. Glossa *4*.

30. Gygax, P.M., Elmiger, D., Zufferey, S., Garnham, A., Sczesny, S., von Stockhausen, L., Braun, F., and Oakhill, J. (2019). A Language Index of Grammatical Gender Dimensions to Study the Impact of Grammatical Gender on the Way We Perceive Women and Men. Front. Psychol. *10*, 1604. https://doi.org/10.3389/fpsyg.2019.01604.

31. Stahlberg, D., Braun, F., Irmen, L., and Sczesny, S. (2007). Representation of the Sexes in Language. Soc. Commun. 163–187.

32. Hord, L.C. (2016). Bucking the linguistic binary: Gender neutral language in English, Swedish, French, and German. Western Papers in Linguistics/Cahiers linguistiques de Western *3*, 4.

33. Hellinger, M., and Motschenbacher, H. (2015). Gender across Languages. The Linguistic Representation of Women and Men, *vol. IV* (John Benjamins).

34. Gheno, V. (2019). Femminili Singolari: Il Femminismo È Nelle Parole (Effequ).

35. Westveer, T., Sleeman, P., and Aboh, E.O. (2018). Discriminating dictionaries? feminine forms of profession nouns in dictionaries of french and german. Int. J. Lexicogr. *31*, 371–393. https://doi.org/10.1093/ijl/ecy013.

36. Sczesny, S., Formanowicz, M., and Moser, F. (2016). Can gender-fair language reduce gender stereotyping and discrimination? Front. Psychol. *7*, 25. https://doi.org/10.3389/fpsyg.2016.00025.

37. Silveira, J. (1980). Generic masculine words and thinking. Wom. Stud. Int. Q. *3*, 165–178. https://doi.org/10.1016/S0148-0685(80)92113-2.

38. Braun, F., Sczesny, S., and Stahlberg, D. (2005). Cognitive effects of masculine generics in german: An overview of empirical findings. Communications *30*, 1–21. https://doi.org/10.1515/comm.2005.30.1.1.

39. Ansara, Y.G., and Hegarty, P. (2014). Methodologies of misgendering: Recommendations for reducing cisgenderism in psychological research. Fem. Psychol. *24*, 259–270. https://doi.org/10.1177/0959353514526217.

40. McLemore, K.A. (2018). A minority stress perspective on transgender individuals' experiences with misgendering. Stigma and Health *3*, 53–64. https://doi.org/10.1037/sah0000070.

41. Strengers, Y., Qu, L., Xu, Q., and Knibbe, J. (2020). Adhering, steering, and queering: Treatment of gender in natural language generation Proceedings of the 2020 CHI Conference on Human Factors in Computing Systems. CHIPS *20*, 1–14. https://doi.org/10.1145/3313831.3376315.

42. Lauscher, A., Crowley, A., and Hovy, D. (2022). Welcome to the modern world of pronouns: Identity-inclusive natural language processing beyond gender. In Proceedings of the 29th International Conference on Computational Linguistics, pp. 1221–1232.

43. Gustafsson Sendén, M., Renström, E., and Lindqvist, A. (2021). Pronouns beyond the binary: The change of attitudes and use over time. Gend. Soc. *35*, 588–615.

44. Knisely, K.A. (2020). Le français non-binaire: Linguistic forms used by non-binary speakers of French. Foreign Lang. Ann. *53*, 850–876. https://doi.org/10.1111/flan.12500.

45. Attig, R., and López, A. (2020). Queer Community Input in Gender-Inclusive Translations (Linguistic Society of America). [Blog].

46. Scharrón-del Río, M.R., and Aja, A.A. (2020). Latinx: Inclusive language as liberation praxis. Journal of Latinx Psychology *8*, 7–20. https://doi.org/10.1037/lat0000140.

47. Comandini, G. (2021). Salve a tutte, tutt*, tuttu, tuttx e tutt@: l'uso delle strategie di neutralizzazione di genere nella comunità queer online. Testo e Senso *23*, 43–64.

48. Vanmassenhove, E. (2024). 9 gender bias in machine translation and the era of large language models. In Gendered Technology in Translation and Interpreting: Centering Rights in the Development of Language Technology, p. 225. https://doi.org/10.4324/9781003465508-12.

49. Piazzolla, S.A., Savoldi, B., and Bentivogli, L. (2024). Good, but not always fair: An evaluation of gender bias for three commercial machine translation systems. In HERMES-Journal of Language and Communication in Business, pp. 209–225. https://doi.org/10.7146/hjlcb.vi63.137553.

50. Page, M.J., McKenzie, J.E., Bossuyt, P.M., Boutron, I., Hoffmann, T.C., Mulrow, C.D., Shamseer, L., Tetzlaff, J.M., Akl, E.A., Brennan, S.E., et al. (2021). The prisma 2020 statement: an updated guideline for reporting systematic reviews. Britisch Medical Journal (BMJ) *372*, n71. https://doi.org/10.1136/bmj.n71.

51. Cho, W.I., Kim, J., Yang, J., and Kim, N.S. (2021). Towards cross-lingual generalization of translation gender bias. In Proceedings of the 2021 ACM Conference on Fairness, Accountability, and Transparency. FAccT '21, pp. 449–457. https://doi.org/10.1145/3442188.3445907.

52. Corbett, G.G. (1991). Gender. Cambridge Textbooks in Linguistics (Cambridge University Press). https://doi.org/10.1017/CBO9781139166119.

53. Sun, T., Gaut, A., Tang, S., Huang, Y., ElSherief, M., Zhao, J., Mirza, D., Belding, E., Chang, K.-W., and Wang, W.Y. (2019). Mitigating gender bias in natural language processing: Literature review. Florence, Italy. In Proceedings of the 57th Annual Meeting of the Association for Computational Linguistics, pp. 1630–1640. https://doi.org/10.18653/v1/P19-1159.

54. Costa-jussà, M.R. (2019). An analysis of Gender Bias studies in Natural Language Processing. Nat. Mach. Intell. *1*, 495–496. https://doi.org/10.1038/s42256-019-0105-5.

55. M.R. Costa-jussà, C. Hardmeier, W. Radford, and K. Webster, eds. (2019). Proceedings of the First Workshop on Gender Bias in Natural Language Processing (Florence, Italy: Association for Computational Linguistics).

56. E. Vanmassenhove, B. Savoldi, L. Bentivogli, J. Daems, and J. Hackenbuchner, eds. (2023). Proceedings of the First Workshop on Gender-Inclusive Translation Technologies (Tampere, Finland: European Association for Machine Translation).

57. van der Wees, M., Bisazza, A., and Monz, C. (2016). Measuring the effect of conversational aspects on machine translation quality. In Proceedings of COLING 2016, the 26th International Conference on Computational Linguistics: Technical Papers, pp. 2571–2581.

58. Bawden, R., Wisniewski, G., and Maynard, H. (2016). Investigating gender adaptation for speech translation. In Actes de la conférence conjointe JEP-TALN-RECITAL 2016(Volume 2: TALN (Posters), pp. 490–497.

59. Rabinovich, E., Patel, R.N., Mirkin, S., Specia, L., and Wintner, S. (2017). Personalized machine translation: Preserving original author traits. Valencia, Spain. In Proceedings of the 15th Conference of the European Chapter of the Association for Computational Linguistics(Volume 1: Long Papers), pp. 1074–1084. https://doi.org/10.18653/v1/E17-1101.

60. Vanmassenhove, E., Hardmeier, C., and Way, A. (2018). Getting gender right in neural machine translation. In Proceedings of the 2018 Conference on Empirical Methods in Natural Language Processing, pp. 3003–3008.

61. Liu, D., and Niehues, J. (2024). How transferable are attribute controllers on pretrained multilingual translation models? In Proceedings of the 18th Conference of the European Chapter of the Association for Computational Linguistics, *1* (Long Papers), pp. 334–348.

62. Sandoval, S., Zhao, J., Carpuat, M., and Daumé III, H. (2023). A rose by any other name would not smell as sweet: Social bias in names mistranslation. In Proceedings of the 2023 Conference on Empirical Methods in Natural Language Processing, pp. 3933–3945. https://doi.org/10.18653/v1/2023.emnlp-main.239.

63. Daems, J., and Hackenbuchner, J. (2022). DeBiasByUs: Raising awareness and creating a database of MT bias. In Proceedings of the 23rd Annual Conference of the European Association for Machine Translation, pp. 289–290.

64. Alrowili, S., and Shanker, V. (2022). Generative approach for gender-rewriting task with arabict5. In Proceedings of the Seventh Arabic Natural Language Processing Workshop (WANLP), pp. 491–495. https://doi.org/10.18653/v1/2022.wanlp-1.55.

65. Nunziatini, M., and Diego, S. (2024). Implementing gender-inclusivity in mt output using automatic post-editing with llms. 1. In Proceedings of the 25th Annual Conference of the European Association for Machine Translation, pp. 580–589.

66. Mash, A., Escolano, C., Sant, A., Melero, M., and de Luca Fornaciari, F. (2024). Unmasking biases: Exploring gender bias in English-Catalan machine translation through tokenization analysis and novel dataset. In Proceedings of the 2024 Joint International Conference on Computational Linguistics, Language Resources and Evaluation (LREC-COLING 2024), pp. 17144–17153.

67. Robinson, K., Kudugunta, S., Stella, R., Dev, S., and Bastings, J. (2024). MiTTenS: A dataset for evaluating gender mistranslation. In Proceedings of the 2024 Conference on Empirical Methods in Natural Language Processing, pp. 4115–4124. https://doi.org/10.18653/v1/2024.emnlp-main.238.

68. Savoldi, B., Piergentili, A., Fucci, D., Negri, M., and Bentivogli, L. (2024). A prompt response to the demand for automatic gender-neutral translation. In Proceedings of the 18th Conference of the European Chapter of the Association for Computational Linguistics (Volume 2: Short Papers), pp. 256–267.

69. Kotek, H., Dockum, R., and Sun, D. (2023). Gender bias and stereotypes in large language models. In Proceedings of The ACM Collective Intelligence Conference. CI '23, pp. 12–24. https://doi.org/10.1145/3582269.3615599.

70. Gaido, M., Savoldi, B., Bentivogli, L., Negri, M., and Turchi, M. (2020). Breeding gender-aware direct speech translation systems. In Proceedings of the 28th International Conference on Computational Linguistics, pp. 3951–3964. https://doi.org/10.18653/v1/2020.coling-main.350.

71. Bentivogli, L., Savoldi, B., Negri, M., Di Gangi, M.A., Cattoni, R., and Turchi, M. (2020). Gender in danger? evaluating speech translation technology on the MuST-SHE corpus. In Proceedings of the 58th Annual Meeting of the Association for Computational Linguistics, pp. 6923–6933. https://doi.org/10.18653/v1/2020.acl-main.619.

72. Gaido, M., Savoldi, B., Bentivogli, L., Negri, M., and Turchi, M. (2021). How to split: The effect of word segmentation on gender bias in speech translation. Findings of the Association for Computational Linguistics: ACL-IJCNLP *2021*, 3576–3589. https://doi.org/10.18653/v1/2021.findings-acl.313.

73. Costa-jussà, M.R., Basta, C., and Gállego, G.I. (2022). Evaluating gender bias in speech translation. In Proceedings of the Thirteenth Language Resources and Evaluation Conference, pp. 2141–2147. https://doi.org/10.18653/v1/2022.lrec-1.230.

74. Savoldi, B., Gaido, M., Bentivogli, L., Negri, M., and Turchi, M. (2022). On the dynamics of gender learning in speech translation. In Proceedings of the 4th Workshop on Gender Bias in Natural Language Processing (GeBNLP), pp. 94–111. https://doi.org/10.18653/v1/2022.gebnlp-1.12.

75. Savoldi, B., Gaido, M., Bentivogli, L., Negri, M., and Turchi, M. (2022). Under the morphosyntactic lens: A multifaceted evaluation of gender bias in speech translation. In Proceedings of the 60th Annual Meeting of the Association for Computational Linguistics (Volume 1: Long Papers), pp. 1807–1824. https://doi.org/10.18653/v1/2022.acl-long.127.

76. Fucci, D., Gaido, M., Papi, S., Cettolo, M., Negri, M., and Bentivogli, L. (2023). Integrating language models into direct speech translation: An inference-time solution to control gender inflection. In Proceedings of the 2023 Conference on Empirical Methods in Natural Language Processing, pp. 11505–11517. Singapore. https://doi.org/10.18653/v1/2023.emnlp-main.705.

77. Caglayan, O., Ive, J., Haralampieva, V., Madhyastha, P., Barrault, L., and Specia, L. (2020). Simultaneous machine translation with visual context. In Proceedings of the 2020 Conference on Empirical Methods in Natural Language Processing (EMNLP), pp. 2350–2361. https://doi.org/10.18653/v1/2020.emnlp-main.184.

78. Savoldi, B., Gaido, M., Negri, M., and Bentivogli, L. (2024). FBK@IWSLT test suites task: Gender bias evaluation with MuST-SHE. In Proceedings of the 21st International Conference on Spoken Language Translation (IWSLT 2024), pp. 65–71. https://doi.org/10.18653/v1/2024.iwslt-1.10.

79. Post, M., and Junczys-Dowmunt, M. (2024). Escaping the sentence-level paradigm in machine translation. Preprint at arXiv. https://doi.org/10.48550/arXiv.2304.12959.

80. Basta, C., Costa-jussà, M.R., and Fonollosa, J.A.R. (2020). Towards mitigating gender bias in a decoder-based neural machine translation model by adding contextual information. In Proceedings of the The Fourth Widening Natural Language Processing Workshop, pp. 99–102. https://doi.org/10.18653/v1/2020.winlp-1.25.

81. Stojanovski, D., Krojer, B., Peskov, D., and Fraser, A. (2020). ContraCAT: Contrastive coreference analytical templates for machine translation. In Proceedings of the 28th International Conference on Computational Linguistics, pp. 4732–4749. https://doi.org/10.18653/v1/2020.coling-main.417.

82. Gete, H., Etchegoyhen, T., Ponce, D., Labaka, G., Aranberri, N., Corral, A., Saralegi, X., Ellakuria, I., and Martin, M. (2022). Tando: A corpus for document-level machine translation. In Proceedings of the Thirteenth Language Resources and Evaluation Conference, pp. 3026–3037.

83. Currey, A., Nădejde, M., Pappagari, R.R., Mayer, M., Lauly, S., Niu, X., Hsu, B., and Dinu, G. (2022). Mt-geneval: A counterfactual and contextual dataset for evaluating gender accuracy in machine translation. In Proceedings of the 2022 Conference on Empirical Methods in Natural Language Processing, pp. 4287–4299.

84. Gete, H., and Etchegoyhen, T. (2023). An evaluation of source factors in concatenation-based context-aware neural machine translation. In Proceedings of the 14th International Conference on Recent Advances in Natural Language Processing, pp. 399–407.

85. Wicks, R., and Post, M. (2023). Identifying context-dependent translations for evaluation set production. In Proceedings of the Eighth Conference on Machine Translation, pp. 452–467. https://doi.org/10.18653/v1/2023.wmt-1.42.

86. Castilho, S., Mallon, C.Q., Meister, R., and Yue, S. (2023). Do online machine translation systems care for context? what about a gpt model? In Proceedings of the 24th Annual Conference of the European Association for Machine Translation, pp. 393–417.

87. Vincent, S., Flynn, R., and Scarton, C. (2023). Mtcue: Learning zero-shot control of extra-textual attributes by leveraging unstructured context in neural machine translation. Toronto, Canada. In Findings of the Association for Computational Linguistics: ACL 2023, pp. 8210–8226. https://doi.org/10.18653/v1/2023.findings-acl.521.

88. Gete, H., and Etchegoyhen, T. (2024). Does context help mitigate gender bias in neural machine translation? Findings of the Association for Computational Linguistics: EMNLP *14788*, 14788–14794. https://doi.org/10.18653/v1/2024.findings-emnlp.868.

89. Lardelli, M., Attanasio, G., and Lauscher, A. (2024). Building bridges: A dataset for evaluating gender-fair machine translation into German. In Findings of the Association for Computational Linguistics: ACL 2024, pp. 7542–7550. https://doi.org/10.18653/v1/2024.findings-acl.448.

90. Vincent, S. (2021). Towards personalised and document-level machine translation of dialogue. In Proceedings of the 16th Conference of the European Chapter of the Association for Computational Linguistics: Student Research Workshop, pp. 137–147. https://doi.org/10.18653/v1/2021.eacl-srw.19.

91. Castilho, S., Cavalheiro Camargo, J.L., Menezes, M., and Way, A. (2021). Dela corpus - a document-level corpus annotated with context-related issues. In Proceedings of the Sixth Conference on Machine Translation, pp. 566–577. Online.

92. Castilho, S. (2022). How much context span is enough? examining context-related issues for document-level MT. In Proceedings of the Thirteenth Language Resources and Evaluation Conference, pp. 3017–3025.

93. Daems, J. (2023). Gender-inclusive translation for a gender-inclusive sport: strategies and translator perceptions at the international quadball association. In Proceedings of the First Workshop on Gender-Inclusive Translation Technologies, pp. 37–47.

94. Castilho, S., Popović, M., and Way, A. (2020). On context span needed for machine translation evaluation. In Proceedings of the Twelfth Language Resources and Evaluation Conference, pp. 3735–3742.

95. Vincent, S.T., Barrault, L., and Scarton, C. (2022). Controlling extra-textual attributes about dialogue participants: A case study of English-to-Polish neural machine translation. In Proceedings of the 23rd Annual Conference of the European Association for Machine Translation, pp. 121–130.

96. Kostikova, A., Daems, J., and Lazarov, T. (2023). How adaptive is adaptive machine translation, really? a gender-neutral language use case. In Proceedings of the First Workshop on Gender-Inclusive Translation Technologies, pp. 95–97.

97. Saunders, D., Sallis, R., and Byrne, B. (2020). Neural machine translation doesn't translate gender coreference right unless you make it. In Proceedings of the Second Workshop on Gender Bias in Natural Language Processing, pp. 35–43.

98. Escolano, C., Ojeda, G., Basta, C., and Costa-jussa, M.R. (2021). Multitask learning for improving gender accuracy in neural machine translation. In Proceedings of the 18th International Conference on Natural Language Processing (ICON), pp. 12–17.

99. Escudé Font, J., and Costa-jussà, M.R. (2019). Equalizing gender bias in neural machine translation with word embeddings techniques. In Proceedings of the First Workshop on Gender Bias in Natural Language Processing, pp. 147–154. https://doi.org/10.18653/v1/W19-3821.

100. Le, N.T., Hansal, O., and Sadat, F. (2023). Challenges and issue of gender bias in under-represented languages: An empirical study on Inuktitut-English NMT. In Proceedings of the Sixth Workshop on the Use of Computational Methods in the Study of Endangered Languages, pp. 89–97.

101. Iluz, B., Elazar, Y., Yehudai, A., and Stanovsky, G. (2024). Applying intrinsic debiasing on downstream tasks: Challenges and considerations for machine translation. In Proceedings of the 2024 Conference on Empirical Methods in Natural Language Processing, pp. 14914–14921. https://doi.org/10.18653/v1/2024.emnlp-main.829.

102. Moryossef, A., Aharoni, R., and Goldberg, Y. (2019). Filling gender & number gaps in neural machine translation with black-box context injection. In Proceedings of the First Workshop on Gender Bias in Natural Language Processing, pp. 49–54. https://doi.org/10.18653/v1/W19-3807.

103. Lu, T., Aepli, N., and Rios, A. (2023). Reducing gender bias in NMT with FUDGE. In Proceedings of the First Workshop on Gender-Inclusive Translation Technologies, pp. 61–69.

104. Jain, N., Popović, M., Groves, D., and Vanmassenhove, E. (2021). Generating gender augmented data for NLP. In Proceedings of the 3rd Workshop on Gender Bias in Natural Language Processing, pp. 93–102. https://doi.org/10.18653/v1/2021.gebnlp-1.11.

105. Alhafni, B., Habash, N., and Bouamor, H. (2022). User-centric gender rewriting. In Proceedings of the 2022 Conference of the North American Chapter of the Association for Computational Linguistics: Human Language Technologies, pp. 618–631. https://doi.org/10.18653/v1/2022.naacl-main.46.

106. Vanmassenhove, E., Emmery, C., and Shterionov, D. (2021). Neutral rewriter: A rule-based and neural approach to automatic rewriting into gender neutral alternatives. In Proceedings of the 2021 Conference on Empirical Methods in Natural Language Processing, pp. 8940–8948. https://doi.org/10.18653/v1/2021.emnlp-main.704.

107. Veloso, L., Coheur, L., and Ribeiro, R. (2023). A rewriting approach for gender inclusivity in Portuguese. In Findings of the Association for Computational Linguistics: EMNLP 2023, pp. 8747–8759. https://doi.org/10.18653/v1/2023.findings-emnlp.585.

108. Amrhein, C., Schottmann, F., Sennrich, R., and Läubli, S. (2023). Exploiting biased models to de-bias text: A gender-fair rewriting model. In Proceedings of the 61st Annual Meeting of the Association for Computational Linguistics (Volume 1: Long Papers), pp. 4486–4506. https://doi.org/10.18653/v1/2023.acl-long.246.

109. Attanasio, G., Plaza del Arco, F.M., Nozza, D., and Lauscher, A. (2023). A tale of pronouns: Interpretability informs gender bias mitigation for fairer instruction-tuned machine translation. In Proceedings of the 2023 Conference on Empirical Methods in Natural Language Processing, pp. 3996–4014. https://doi.org/10.18653/v1/2023.emnlp-main.243.

110. Garg, S., Gheini, M., Emmanuel, C., Likhomanenko, T., Gao, Q., and Paulik, M. (2024). Generating gender alternatives in machine translation. In Proceedings of the 5th Workshop on Gender Bias in Natural Language Processing (GeBNLP), pp. 237–254. https://doi.org/10.18653/v1/2024.gebnlp-1.15.

111. Devinney, H., Björklund, J., and Björklund, H. (2022). Theories of "gender" in nlp bias research. In Proceedings of the 2022 ACM Conference on Fairness, Accountability, and Transparency. FAccT '22 (2083–2102). https://doi.org/10.1145/3531146.3534627.

112. Saunders, D., Sallis, R., and Byrne, B. (2022). First the worst: Finding better gender translations during beam search. Findings of the Association for Computational Linguistics: ACL *2022*, 3814–3823. https://doi.org/10.18653/v1/2022.findings-acl.301.

113. Savoldi, B., Gaido, M., Negri, M., and Bentivogli, L. (2023). Test suites task: Evaluation of gender fairness in MT with MuST-SHE and INES. In Proceedings of the Eighth Conference on Machine Translation, pp. 252–262. https://doi.org/10.18653/v1/2023.wmt-1.25.

114. Saunders, D., and Olsen, K. (2023). Gender, names and other mysteries: Towards the ambiguous for gender-inclusive translation. In Proceedings of the First Workshop on Gender-Inclusive Translation Technologies, pp. 85–93.

115. Rarrick, S., Naik, R., Poudel, S., and Chowdhary, V. (2024). GATE X-E : A challenge set for gender-fair translations from weakly-gendered languages. In Findings of the Association for Computational Linguistics: ACL 2024, pp. 8526–8546. https://doi.org/10.18653/v1/2024.findings-acl.504.

116. Vanmassenhove, E., Emmery, C., and Shterionov, D. (2021). NeuTral Rewriter: A rule-based and neural approach to automatic rewriting into gender neutral alternatives. In Proceedings of the 2021 Conference on Empirical Methods in Natural Language Processing, pp. 8940–8948. https://doi.org/10.18653/v1/2021.emnlp-main.704.

117. Lardelli, M., and Gromann, D. (2023). Gender-fair post-editing: A case study beyond the binary. In Proceedings of the 24th Annual Conference of the European Association for Machine Translation, pp. 251–260.

118. Piergentili, A., Fucci, D., Savoldi, B., Bentivogli, L., and Negri, M. (2023). Gender neutralization for an inclusive machine translation: from theoretical foundations to open challenges. In Proceedings of the First Workshop on Gender-Inclusive Translation Technologies, pp. 71–83.

CellPress
OPEN ACCESS

119. Piergentili, A., Savoldi, B., Fucci, D., Negri, M., and Bentivogli, L. (2023). Hi guys or hi folks? benchmarking gender-neutral machine translation with the GeNTE corpus. In Proceedings of the 2023 Conference on Empirical Methods in Natural Language Processing, pp. 14124–14140. https://doi.org/10.18653/v1/2023.emnlp-main.873.

120. Lauscher, A., Nozza, D., Miltersen, E., Crowley, A., and Hovy, D. (2023). What about “em”? how commercial machine translation fails to handle (neo-)pronouns. In Proceedings of the 61st Annual Meeting of the Association for Computational Linguistics (Volume 1: Long Papers), pp. 377–392. https://doi.org/10.18653/v1/2023.acl-long.23.

121. Crenshaw, K. (1989). Demarginalizing the intersection of race and sex: A black feminist critique of antidiscrimination doctrine, feminist theory and antiracist politics. Feminism and Politics *1*, 139–167.

122. Schlesinger, A., Edwards, W.K., and Grinter, R.E. (2017). Intersectional hci: Engaging identity through gender, race, and class. In Proceedings of the 2017 CHI Conference on Human Factors in Computing Systems. CHI ’17, pp. 5412–5427. https://doi.org/10.1145/3025453.3025766.

123. Buolamwini, J., and Gebru, T. (2018). Gender Shades: Intersectional Accuracy Disparities in Commercial Gender Classification. 81. In Proceedings of Machine Learning Research, pp. 77–91.

124. Hovy, D., Bianchi, F., and Fornaciari, T. (2020). “you sound just like your father” commercial machine translation systems include stylistic biases. In Proceedings of the 58th Annual Meeting of the Association for Computational Linguistics, pp. 1686–1690. https://doi.org/10.18653/v1/2020.acl-main.154.

125. Wang, J., Rubinstein, B., and Cohn, T. (2022). Measuring and mitigating name biases in neural machine translation. In Proceedings of the 60th Annual Meeting of the Association for Computational Linguistics, *1* (Long Papers)), pp. 2576–2590. https://doi.org/10.18653/v1/2022.acl-long.184.

126. Stewart, I., and Mihalcea, R. (2024). Whose wife is it anyway? assessing bias against same-gender relationships in machine translation. In Proceedings of the 5th Workshop on Gender Bias in Natural Language Processing (GeBNLP), pp. 365–375. https://doi.org/10.18653/v1/2024.gebnlp-1.23.

127. Costa-jussà, M., Smith, E., Ropers, C., Licht, D., Maillard, J., Ferrando, J., and Escolano, C. (2023). Toxicity in multilingual machine translation at scale. In Findings of the Association for Computational Linguistics: EMNLP 2023, pp. 9570–9586. https://doi.org/10.18653/v1/2023.findings-emnlp.642.

128. Soler Uguet, C., Bane, F., Aymo, M., Fernandes Torres, J.P., Zaretskaya, A., and Blanch Miró, T. (2023). Enhancing gender representation in neural machine translation: A comparative analysis of annotating strategies for English-Spanish and English-Polish language pairs. In Proceedings of Machine Translation Summit XIX, *2* (Users Track), pp. 171–172.

129. Kocmi, T., Limisiewicz, T., and Stanovsky, G. (2020). Gender coreference and bias evaluation at WMT 2020. In Proceedings of the Fifth Conference on Machine Translation, pp. 357–364.

130. Popović, M. (2021). Agree to disagree: Analysis of inter-annotator disagreements in human evaluation of machine translation output. In Proceedings of the 25th Conference on Computational Natural Language Learning, pp. 234–243. https://doi.org/10.18653/v1/2021.conll-1.18.

131. Gromann, D., Lardelli, M., Spiel, K., Burtscher, S., Klausner, L.D., Mettinger, A., Miladinovic, I., Schefer-Wenzl, S., Duh, D., and Bühn, K. (2023). Participatory research as a path to community-informed, gender-fair machine translation. In Proceedings of the First Workshop on Gender-Inclusive Translation Technologies, pp. 49–59.

132. Prates, M.O.R., Avelar, P.H., and Lamb, L.C. (2020). Assessing gender bias in machine translation: a case study with google translate. Neural Comput. Appl. *32*, 6363–6381. https://doi.org/10.1007/s00521-019-04144-6.

133. Janusheva, V. (2023). The gender-specific nouns denoting profession/role: The macedonian case. LL J. *26*, 115–133. https://doi.org/10.24071/llt.v26i1.5645.

134. López, Á., Rodríguez Barcia, S., and Cabeza Pereiro, M.d.C. (2020). Visibilizar o interpretar: respuesta al informe de la real academia española sobre el lenguaje inclusivo y cuestiones conexas. Anuario de glotopolítica.

135. Hamidi, F., Scheuerman, M.K., and Branham, S.M. (2018). Gender Recognition or Gender Reductionism? The Social Implications of Embedded Gender Recognition Systems Proceedings of the 2018 CHI Conference on Human Factors in Computing Systems. CHIPS *18*, 1–13. https://doi.org/10.1145/3173574.3173582.

136. Zimman, L., Hazenberg, E., and Meyerhoff, M. (2017). Trans people’s linguistic self-determination and the dialogic nature of identity. In Representing trans: Linguistic, legal and everyday perspectives, pp. 226–248.

137. Hu, Y., Wu, D., and Nucci, A. (2012). Pitch-based gender identification with two-stage classification. Secur. Commun. Network. *5*, 211–225. https://doi.org/10.1002/sec.308.

138. Li, B., Lian, X.-C., and Lu, B.-L. (2012). Gender classification by combining clothing, hair and facial component classifiers. Neurocomputing *76*, 18–27. https://doi.org/10.1016/j.neucom.2011.01.028.

139. Vanmassenhove, E., and Monti, J. (2021). gENder-IT: An annotated English-Italian parallel challenge set for cross-linguistic natural gender phenomena. In Proceedings of the 3rd Workshop on Gender Bias in Natural Language Processing, pp. 1–7. https://doi.org/10.18653/v1/2021.gebnlp-1.1.

140. Campolo, A., Sanfilippo, M.R., Whittaker, M., and Crawford, K. (2017). AI Now Report 2017 (AI Now Institute).

141. Friedman, B., and Nissenbaum, H. (1996). Bias in Computer Systems. ACM Trans. Inf. Syst. *14*, 330–347. https://doi.org/10.1145/230538.230561.

142. Friedler, S.A., Scheidegger, C., and Venkatasubramanian, S. (2021). The (im)possibility of fairness: different value systems require different mechanisms for fair decision making. Commun. ACM *64*, 136–143. https://doi.org/10.1145/3433949.

143. Jernite, Y. (2022). Let’s Talk about Biases in Machine Learning (Ethics and Society Newsletter #2).

144. Ponti, E.M., O’Horan, H., Berzak, Y., Vulić, I., Reichart, R., Poibeau, T., Shutova, E., and Korhonen, A. (2019). Modeling language variation and universals: A survey on typological linguistics for natural language processing. Comput. Linguist. *45*, 559–601. https://doi.org/10.1162/coli_a_00357.

145. Helm, P., Bella, G., Koch, G., and Giunchiglia, F. (2023). Diversity and language technology: how techno-linguistic bias can cause epistemic injustice. Preprint at arXiv. https://doi.org/10.48550/arXiv.2307.13714.

146. Lai, V., Ngo, N., Veyseh, A.P.B., Mn, H., Dernoncourt, F., Bui, T., and Nguyen, T. (2023). Chatgpt beyond english: Towards a comprehensive evaluation of large language models in multilingual learning. In Findings of the Association for Computational Linguistics: EMNLP 2023, pp. 13171–13189.

147. Ahia, O., Kumar, S., Gonen, H., Kasai, J., Mortensen, D.R., Smith, N.A., and Tsvetkov, Y. (2023). Do all languages cost the same? tokenization in the era of commercial language models. In Proceedings of the 2023 Conference on Empirical Methods in Natural Language Processing, pp. 9904–9923.

148. Holtermann, C., Röttger, P., Dill, T., and Lauscher, A. (2024). Evaluating the elementary multilingual capabilities of large language models with multiq. In Findings of the Association for Computational Linguistics ACL 2024, pp. 4476–4494.

149. Zhang, X., Li, S., Hauer, B., Shi, N., and Kondrak, G. (2023). Don’t trust ChatGPT when your question is not in English: A study of multilingual abilities and types of LLMs. In Proceedings of the 2023 Conference on Empirical Methods in Natural Language Processing, pp. 7915–7927. https://doi.org/10.18653/v1/2023.emnlp-main.491.

150. Petrov, A., La Malfa, E., Torr, P., and Bibi, A. (2023). Language model tokenizers introduce unfairness between languages. Adv. Neural Inf. Process. Syst. *36*, 36963–36990.

151. Brown, T., Mann, B., Ryder, N., Subbiah, M., Kaplan, J.D., Dhariwal, P., Neelakantan, A., Shyam, P., Sastry, G., Askell, A., et al. (2020). Language models are few-shot learners. Adv. Neural Inf. Process. Syst. *33*, 1877–1901.

152. Ouyang, L., Wu, J., Jiang, X., Almeida, D., Wainwright, C., Mishkin, P., Zhang, C., Agarwal, S., Slama, K., Ray, A., et al. (2022). Training language models to follow instructions with human feedback. Adv. Neural Inf. Process. Syst. *35*, 27730–27744.

153. Talat, Z., Névéol, A., Biderman, S., Clinciu, M., Dey, M., Longpre, S., Luccioni, S., Masoud, M., Mitchell, M., Radev, D., et al. (2022). You reap what you sow: On the challenges of bias evaluation under multilingual settings. In Proceedings of BigScience Episode #5 – Workshop on Challenges & Perspectives in Creating Large Language Models, pp. 26–41. https://doi.org/10.18653/v1/2022.bigscience-1.3.

154. Cao, Y.T., Sotnikova, A., Zhao, J., Zou, L.X., Rudinger, R., and Daume, I.I. I.,H. (2023). Multilingual large language models leak human stereotypes across language boundaries. Preprint at arXiv. https://doi.org/10.48550/arXiv.2312.07141.

155. Choenni, R., Lauscher, A., and Shutova, E. (2024). The echoes of multilinguality: Tracing cultural value shifts during language model fine-tuning. In Proceedings of the 62nd Annual Meeting of the Association for Computational Linguistics, *1* (Long Papers), pp. 15042–15058.

156. Naous, T., Ryan, M.J., Ritter, A., and Xu, W. (2024). Having beer after prayer? measuring cultural bias in large language models. In Proceedings of the 62nd Annual Meeting of the Association for Computational Linguistics (Volume 1: Long Papers), pp. 16366–16393.

157. Luthra, M., and Nijman, B. (2024). Lost in Translation? Approaches to Gender Representation in Multilingual Archives. In Proceedings of the 2nd International Workshop on Gender-Inclusive Translation Technologies, pp. 42–55.

158. Connell, R. (2020). Gender. In World Perspective (John Wiley & Sons).

159. Chakrapani, V. (2010). Hijras/transgender women in india: Hiv, human rights and social exclusion. In South Asian Born-Digital NGO Reports Collection Project.

160. Hossain, A. (2017). The paradox of recognition: hijra, third gender and sexual rights in bangladesh. Cult. Health Sex. *19*, 1418–1431.

161. Jacobs, S.-E., Thomas, W., and Lang, S. (1997). Two-spirit People: Native American Gender Identity, Sexuality, and Spirituality (University of Illinois Press).

162. Wang, W., Chen, Z., Chen, X., Wu, J., Zhu, X., Zeng, G., Luo, P., Lu, T., Zhou, J., Qiao, Y., et al. (2024). Visionllm: Large language model is also an open-ended decoder for vision-centric tasks. Adv. Neural Inf. Process. Syst. *36*.

163. Liu, H., Li, C., Wu, Q., and Lee, Y.J. (2024). Visual instruction tuning. Adv. Neural Inf. Process. Syst. *36*.

164. Radford, A., Wu, J., Child, R., Luan, D., Amodei, D., and Sutskever, I. (2019). Language models are unsupervised multitask learners. OpenAI blog *1*, 9.

165. Barrault, L., Chung, Y.-A., Meglioli, M.C., Dale, D., Dong, N., Duquenne, P.-A., Elsahar, H., Gong, H., Heffernan, K., Hoffman, J., et al. (2023). SeamlessM4T-Massively Multilingual & Multimodal Machine Translation. Preprint at arXiv. https://doi.org/10.48550/arXiv.2308.11596.

166. Shen, H., Shao, L., Li, W., Lan, Z., Liu, Z., and Su, J. (2024). A Survey on Multi-modal Machine Translation: Tasks, Methods and Challenges. Preprint at arXiv. https://doi.org/10.48550/arXiv.2405.12669.

167. Lyu, C., Du, Z., Xu, J., Duan, Y., Wu, M., Lynn, T., Aji, A.F., Wong, D.F., and Wang, L. (2024). A paradigm shift: The future of machine translation lies with large language models. In Proceedings of the 2024 Joint International Conference on Computational Linguistics, Language Resources and Evaluation (LREC-COLING 2024), pp. 1339–1352.

168. Gaido, M., Papi, S., Negri, M., and Bentivogli, L. (2024). Speech translation with speech foundation models and large language models: What is there and what is missing? In Proceedings of the 62nd Annual Meeting of the Association for Computational Linguistics (Volume 1: Long Papers), pp. 14760–14778.

169. Howard, P., Madasu, A., Le, T., Moreno, G.L., and Lal, V. (2023). Probing Intersectional Biases in Vision-Language Models with Counterfactual Examples. Preprint at arXiv. https://doi.org/10.48550/arXiv.2310.02988.

170. Attanasio, G., Savoldi, B., Fucci, D., and Hovy, D. (2024). Twists, humps, and pebbles: Multilingual speech recognition models exhibit gender performance gaps. In Proceedings of the 2024 Conference on Empirical Methods in Natural Language Processing, pp. 21318–21340. https://doi.org/10.18653/v1/2024.emnlp-main.1188.

171. Agnew, W., Bergman, A.S., Chien, J., Díaz, M., El-Sayed, S., Pittman, J., Mohamed, S., and McKee, K.R. (2024). The illusion of artificial inclusion. In Proceedings of the CHI Conference on Human Factors in Computing Systems, pp. 1–12.

172. Savoldi, B., Ramponi, A., Negri, M., and Bentivogli, L. (2025). Translation in the hands of many:centering lay users in machine translation interactions. Preprint at arXiv. https://doi.org/10.48550/arXiv.2502.13780.

173. Ibrahim, L., Huang, S., Ahmad, L., and Anderljung, M. (2024). Beyond static AI evaluations: advancing human interaction evaluations for LLM harms and risks. Preprint at arXiv. https://doi.org/10.48550/arXiv.2405.10632.

174. Lum, K., Anthis, J.R., Nagpal, C., and D'Amour, A. (2024). Bias in Language Models: Beyond Trick Tests and Toward RUTEd Evaluation. Preprint at arXiv. https://doi.org/10.48550/arXiv.2402.12649.

175. Gautam, V., Bingert, E., Zhu, D., Lauscher, A., and Klakow, D. (2024). Robust pronoun fidelity with english llms: Are they reasoning, repeating, or just biased? Transactions of the Association for Computational Linguistics *12*, 1755–1779.

176. Xu, W. (2019). Toward human-centered ai: a perspective from human-computer interaction. interactions *26*, 42–46.

177. Wang, Z.J., Choi, D., Xu, S., and Yang, D. (2021). Putting humans in the natural language processing loop: A survey. In Proceedings of the First Workshop on Bridging Human–Computer Interaction and Natural Language Processing, pp. 47–52.

178. Liao, Q.V., and Xiao, Z. (2023). Rethinking model evaluation as narrowing the socio-technical gap. Preprint at arXiv. https://doi.org/10.48550/arXiv.2306.03100.

179. Lardelli, M., Dill, T., Attanasio, G., and Lauscher, A. (2024). Sparks of fairness: Preliminary evidence of commercial machine translation as english-to-german gender-fair dictionaries. In Proceedings of the 2nd International Workshop on Gender-Inclusive Translation Technologies, pp. 12–21.

180. Moslem, Y., Haque, R., Kelleher, J.D., and Way, A. (2023). Adaptive machine translation with large language models. In Proceedings of the 24th Annual Conference of the European Association for Machine Translation, pp. 227–237.

181. Sarti, G., Htut, P.M., Niu, X., Hsu, B., Currey, A., Dinu, G., and Nadejde, M. (2023). RAMP: Retrieval and attribute-marking enhanced prompting for attribute-controlled translation. In Proceedings of the 61st Annual Meeting of the Association for Computational Linguistics (Volume 2: Short Papers), pp. 1476–1490. https://doi.org/10.18653/v1/2023.acl-short.126.

182. Garcia, X., and Firat, O. (2022). Using natural language prompts for machine translation. Preprint at arXiv. https://doi.org/10.48550/arXiv.2202.11822.

183. Yamada, M. (2023). Optimizing machine translation through prompt engineering: An investigation into chatgpt's customizability. 2. In Proceedings of Machine Translation Summit XIX,, pp. 195–204.

184. Garcia, X., Bansal, Y., Cherry, C., Foster, G., Krikun, M., Johnson, M., and Firat, O. (2023). The unreasonable effectiveness of few-shot learning for machine translation. In Proceedings of the 40th International Conference on Machine Learning vol. 202 of Proceedings of Machine Learning Research, pp. 10867–10878.

185. Piergentili, A., Savoldi, B., Negri, M., and Bentivogli, L. (2024). Enhancing Gender-Inclusive Machine Translation with Neomorphemes and Large Language Models. 1. In Proceedings of the 25th Annual Conference of the European Association for Machine Translation, pp. 300–314.

186. Hossain, T., Dev, S., and Singh, S. (2023). MISGENDERED: Limits of large language models in understanding pronouns. In Proceedings of the 61st Annual Meeting of the Association for Computational Linguistics

CellPress
OPEN ACCESS

(Volume 1: Long Papers), pp. 5352–5367. https://doi.org/10.18653/v1/2023.acl-long.293.

187. Ovalle, A., Mehrabi, N., Goyal, P., Dhamala, J., Chang, K.-W., Zemel, R., Galstyan, A., Pinter, Y., and Gupta, R. (2024). Tokenization matters: Navigating data-scarce tokenization for gender inclusive language technologies. Findings of the Association for Computational Linguistics: NAACL *2024*, 1739–1756.

188. Talat, Z., and Lauscher, A. (2022). Back to the future: On potential histories in NLP. Preprint at arXiv. https://doi.org/10.48550/arXiv.2210.06245.

189. Biderman, S., Schoelkopf, H., Anthony, Q.G., Bradley, H., O'Brien, K., Hallahan, E., Khan, M.A., Purohit, S., Prashanth, U.S., Raff, E., et al. (2023). Pythia: A suite for analyzing large language models across training and scaling. In International Conference on Machine Learning (PMLR), pp. 2397–2430.

190. Bartl, M., and Leavy, S. (2024). From 'showgirls' to 'performers': Fine-tuning with gender-inclusive language for bias reduction in llms. In Proceedings of the 5th Workshop on Gender Bias in Natural Language Processing (GeBNLP), pp. 280–294.

191. Lucy, L., Blodgett, S.L., Shokouhi, M., Wallach, H., and Olteanu, A. (2024). "One-Size-Fits-All"? Examining Expectations around What Constitute "Fair" or "Good" NLG System Behaviors. In Proceedings of the 2024 Conference of the North American Chapter of the Association for Computational Linguistics: Human Language Technologies (Volume 1: Long Papers), pp. 1054–1089. https://doi.org/10.18653/v1/2024.naacl-long.61.

192. Kirk, H.R., Vidgen, B., Röttger, P., and Hale, S.A. (2024). The benefits, risks and bounds of personalizing the alignment of large language models to individuals. Nat. Mach. Intell. *6*, 383–392. https://doi.org/10.1038/s42256-024-00820-y.

193. Anthis, J., Lum, K., Ekstrand, M., Feller, A., D'Amour, A., and Tan, C. (2024). The Impossibility of Fair LLMs. Preprint at arXiv. https://doi.org/10.48550/arXiv.2406.03198.

194. Eapen, J., and Adhithyan, V. (2023). Personalization and customization of llm responses. Int. J. Res. Publ. Rev. *4*, 2617–2627.

195. Mothukuri, V., Parizi, R.M., Pouriyeh, S., Huang, Y., Dehghantanha, A., and Srivastava, G. (2021). A survey on security and privacy of federated learning. Future Gener. Comput. Syst. *115*, 619–640.

196. Wachter, S. (2018). Normative challenges of identification in the internet of things: Privacy, profiling, discrimination, and the gdpr. Computer law & security review *34*, 436–449. https://doi.org/10.1049/cp.2018.0013.

197. Cho, W.I., Kim, J.W., Kim, S.M., and Kim, N.S. (2019). On measuring gender bias in translation of gender-neutral pronouns. In Proceedings of the First Workshop on Gender Bias in Natural Language Processing, pp. 173–181. https://doi.org/10.18653/v1/W19-3824.

198. Stanovsky, G., Smith, N.A., and Zettlemoyer, L. (2019). Evaluating gender bias in machine translation. In Proceedings of the 57th Annual Meeting of the Association for Computational Linguistics, pp. 1679–1684. https://doi.org/10.18653/v1/P19-1164.

199. Saunders, D., and Byrne, B. (2020). Reducing gender bias in neural machine translation as a domain adaptation problem. In Proceedings of the 58th Annual Meeting of the Association for Computational Linguistics, pp. 7724–7736. https://doi.org/10.18653/v1/2020.acl-main.690.

200. Font, J.E., and Costa-jussà, M.R. (2019). Equalizing gender bias in neural machine translation with word embeddings techniques. In Proceedings of the First Workshop on Gender Bias in Natural Language Processing, pp. 147–154. https://doi.org/10.18653/v1/W19-3821.

201. Habash, N., Bouamor, H., and Chung, C. (2019). Automatic gender identification and reinflection in arabic. In Proceedings of the First Workshop on Gender Bias in Natural Language Processing, pp. 155–165. https://doi.org/10.18653/v1/W19-3822.

202. Alhafni, B., Habash, N., and Bouamor, H. (2022). The arabic parallel gender corpus 2.0: Extensions and analyses. In Proceedings of the Thirteenth Language Resources and Evaluation Conference, pp. 1870–1884.

203. Bentivogli, L., Savoldi, B., Negri, M., Di Gangi, M.A., Cattoni, R., and Turchi, M. (2020). Gender in danger? evaluating speech translation technology on the MuST-SHE corpus. In Proceedings of the 58th Annual Meeting of the Association for Computational Linguistics (ACL), pp. 6923–6933. https://doi.org/10.18653/v1/2020.acl-main.619.

204. Renduchintala, A., Díaz, D., Heafield, K., Li, X., and Diab, M. (2021). Gender bias amplification during speed-quality optimization in neural machine translation. In Proceedings of the 59th Annual Meeting of the Association for Computational Linguistics and the 11th International Joint Conference on Natural Language Processing (Volume 2: Short Papers), pp. 99–109. https://doi.org/10.18653/v1/2021.acl-short.15.

205. Levy, S., Lazar, K., and Stanovsky, G. (2021). Collecting a large-scale gender bias dataset for coreference resolution and machine translation. Findings of the Association for Computational Linguistics: EMNLP *2021*, 2470–2480. https://doi.org/10.18653/v1/2021.findings-emnlp.211.

206. Costa-jussà, M., Andrews, P., Smith, E., Hansanti, P., Ropers, C., Kalbassi, E., Gao, C., Licht, D., and Wood, C. (2023). Multilingual holistic bias: Extending descriptors and patterns to unveil demographic biases in languages at scale. In Proceedings of the 2023 Conference on Empirical Methods in Natural Language Processing, pp. 14141–14156. https://doi.org/10.18653/v1/2023.emnlp-main.874.

Patterns, Volume *6*

Supplemental information

A decade of gender bias in machine translation

Beatrice Savoldi, Jasmijn Bastings, Luisa Bentivogli, and Eva Vanmassenhove

Supplemental information

Supplemental Table: Papers' Annotation Guidelines

The annotation guidelines are listed in Table S1.

Field	Description
Focus	Is the focus on gender bias?<br>• GENDER: focus on human gender translation, without explicit mention to the notion of bias<br>• GENDER BIAS: focus on human gender translation, with explicit mention to the notion of bias
Binary	How is gender conceptualized?<br>• BINARY: only binary gender, even if only implicitly<br>• NB-SECONDARY: discusses *at least* a third, non-binary category considered in a minor section<br>• NB - MAIN: centers on non-binary gender
Context	How much context is taken into account?<br>• SENTENCE: no additional context beside the sentence-level<br>• PARAGRAPH: from one up to 10 sentences as context<br>• DOCUMENT: more than 10 sentences as context
Language	What are the considered languages or language pairs?<br>• LANGUAGE CODE: indicate the languages or language pairs pairs considered using two or three letter codes, e.g., en-de for English to German. Consider only the languages for which a gender-based metric score (i.e. disaggregated by gender) is provided, and disregard aggregate overall quality translation scores used only as baselines<br>• NA: the label does not apply. The paper is not experimental and does not consider a specified set of languages.
Bias	Does the paper explicitely engage with the societal notion of gender bias?<br>i.e. it recognizes the societal and ethical component of bias, e.g. by discussing its potential for harms on already disadvantaged groups. Use Bentivogli et al. [1] as reference point of minimal engagement.<br>• YES: it engages with the societal notion of gender bias, at least as much as Bentivogli et al. [1]<br>• NO: it does not engage with the societal notion of gender bias at all, or not as much as Bentivogli et al. [1]
Human Involvement	Are people involved in assessing bias?<br>This excludes annotation work for data creation.<br>• NO: no human participant is involved<br>• MANUAL EVALUATION: human participants are involved for (model-centric) manual evaluations or validations, e.g. to correlate human judgments with automatic metrics<br>• SURVEY: human participants are involved to elicit their feedback, experiences and preferences. Can be further distinguished into SUREVY-NOMT if a survey is a available but not MT model is used in the study<br>• PARTICIPATORY: human participants are actively involved in participatory actions and approaches
Modality	Which modality is considered?<br>• TEXT: text-to-text<br>• AUDIO: speech-to-text<br>• VISUAL: image-guided-text-to-text

Mitigation	Is an approach to improve gender translation or a bias mitigation strategy proposed?<br>• YES: an approach to improve gender translation or a bias mitigation strategy is proposed<br>• NO: approach to improve gender translation or a bias mitigation strategy is not proposed (e.g. only analyses)<br>• COMPARISON: a comparison across baseline models for different modalities or architectures (e.g. document-level MT) is proposed to improve gender translation
Mitigation type	Specify the type of mitigation—only if previous field was annotated as either YES or OTHER.<br>• TRAINING: the mitigation strategy implies retraining from scratch<br>• FINE-TUNING: the mitigation strategy implies dedicated fine-tuning<br>• INFERENCE: the mitigation strategy applies at inference time<br>• DOUBLE-OUTPUT-REWRITER: a rewriter is applied a postprocessing step to the MT output to offer an alternative translation<br>• CONTROLLED LLM GENERATION: the mitigaton strategy relies on dedicated prompts to control the realization of gender translation—either as single or double output<br>• COMPARISON: a baseline model for different modalities or architectures (e.g. document-level MT) is proposed to improve gender translation<br>• OTHER: mitigations that do not fit any of the above, e.g., embeddings interventions, approaches to Statistical MT<br>• NA: the label does not apply, no mitigation strategy is proposed<br>.
Paradigm	Which MT paradigm is considered?<br>• SMT: Statistical Machine Translation<br>• NMT: neural machine translation<br>• LLM: large language models<br>• NA: the label does not apply. There is not explicit mention to any model, or focus only on human translation
Intersectional	Which sociodemographic axis are considered for potential bias and discrimination?<br>More than one label can apply.<br>• GENDER, AGE, RACE, RELIGION, SOCIAL CLASS, SEXUAL ORIENTATION

Table S1: Annotation Guidelines.

Supplemental Note: Full List of Reviewed Papers

2016 van der Wees et al.[2], Bawden et al.[3]

2017 Rabinovich et al.[4], Bawden[5]

2018 Popel[6], Vanmassenhove et al.[7], Michel and Neubig[8]

2019 Moryossef et al.[9], Escudé Font and Costa-jussà[10], Cho et al.[11], Stanovsky et al.[12], Habash et al.[13]

2020 Stafanovičs et al.[14], Basta et al.[15], Costa-jussà and de Jorge[16], Saunders et al.[17], Gonen and Webster[18], Gaido et al.[19], Stojanovski et al.[20], Rescigno et al.[21], Bentivogli et al.[22], Saunders and Byrne[23], Hovy et al.[24], González et al.[25], Kocmi et al.[26], Costa-jussà et al.[27], Caglayan et al.[28]

2021 Troles and Schmid[29], Savoldi et al.[30], Wisniewski et al.[31], Ciora et al.[32], Escolano et al.[33], Ramesh et al.[34], Levy et al.[35], Choubey et al.[36], Gaido et al.[37], Vanmassenhove et al.[38], Popović[39], Vincent[40], Renduchintala et al.[41], Castilho et al.[42], Wisniewski et al.[43], Jain et al.[44], Vamvas and Sennrich[45], Vanmassenhove et al.[46], Vanmassenhove and Monti[47]

2022 Wisniewski et al.[48], Costa-jussà et al.[49], Castilho[50], Gete et al.[51], Sólmundsdóttir et al.[52], Savoldi et al.[53], Měchura[54], Corral and Saralegi[55], Mohammadshahi et al.[56], Saunders et al.[57], Currey et al.[58], Karpinska et al.[59], Zhu et al.[60], Sharma et al.[61], Wisniewski et al.[62], Vincent et al.[63], Daems and Hackenbuchner[64], Wang et al.[65], Renduchintala and Williams[66], Wairagala et al.[67], Savoldi et al.[68], Alrowili and Shanker[69], Alhafni et al.[70],[71]

2023 Savoldi et al.[72], Gete and Etchegoyhen[73], Dinh and Niehues[74], Singh[75], Iluz et al.[76], Alhafni et al.[77], Sandoval et al.[78], Wicks and Post[79], Triboulet and Bouillon[80], Gromann et al.[81], Paolucci et al.[82], Lardelli and Gromann[83], Piergentili et al.[84], Saunders and Olsen[85], Kostikova et al.[86], Costa-jussà et al.[87],[88], Cabrera and Niehues[89], Daems[90], Fucci et al.[91], Piergentili et al.[92], Lu et al.[93], Castilho et al.[94], Paulo et al.[95], Le et al.[96], Sarti et al.[97], Lauscher et al.[98], Vincent et al.[99], Attanasio et al.[100], Lee et al.[101], Wang et al.[102], Veloso et al.[103], Amrhein et al.[104], Soler Uguet et al.[105], Sarti et al.[106]

2024 Savoldi et al.[107], Liu and Niehues[108], Mash et al.[109], Lee et al.[110], Costa-jussà et al.[111], Daems[112], Friðriksdóttir[113], Garg et al.[114], Gete and Etchegoyhen[115], Hackenbuchner et al.[116], Iluz et al.[117], Lardelli et al.[118],[119], Luthra and Nijman[120], Nunziatini and Diego[121], Piergentili et al.[122], Pikuliak et al.[123], Popovic and Lapshinova-Koltunski[124], Rarrick et al.[125], Robinson et al.[126], Sánchez et al.[127], Sant et al.[128], Savoldi et al.[129],[130], Sewunetie et al.[131], Stewart and Mihalcea[132], Üstün et al.[133], Zerva et al.[134]

Supplemental Method: Paper Selection Procedure for Literature Review

Our paper selection procedure was systematic and based on peer-reviewed papers in the ACL Anthology that mentioned machine translation (and paraphrases of that) as well as “gender” (and/or “bias”) in the title and/or abstract. We kept 133 papers that were considered in-scope based on the following parameters:

- MT needs to be the main focus of the paper, not a method towards a different goal. Based on this criterion, we excluded for example papers such as Adelani et al.[135]
- MT can be text-to-text as well as other modalities (e.g., speech-to-text).
- Rewriters for MT output were considered in scope, as they are a popular method to improve output, e.g., to make it more inclusive.
- Even if MT is not part of the core experiments, but the findings are overall intended to also inform and guide more inclusive MT such as in Paolucci et al.[82], then we considered the paper in-scope.
- (Human) gender translation needs to be in focus in the paper. This can be gender translation, gender bias, fairness, etc.

Mind that the above search selection method naturally includes all relevant LLM papers , as they need to focus on translation, and hence are expected to mention “translation” in their title or abstract.

Supplemental References

[S1] Bentivogli, L., Savoldi, B., Negri, M., Di Gangi, M. A., Cattoni, R., and Turchi, M. (2020). Gender in danger? evaluating speech translation technology on the MuST-SHE corpus. Proceedings of the 58th Annual Meeting of the Association for Computational Linguistics (ACL). (6923–6933). 10.18653/v1/2020.acl-main.619.

[S2] van der Wees, M., Bisazza, A., and Monz, C. (2016). Measuring the effect of conversational aspects on machine translation quality. Proceedings of COLING 2016, the 26th International Conference on Computational Linguistics: Technical Papers. (2571–2581).

[S3] Bawden, R., Wisniewski, G., and Maynard, H. (2016). Investigating gender adaptation for speech translation. Actes de la conférence conjointe JEP-TALN-RECITAL 2016. volume 2 : TALN (Posters). (490–497).

[S4] Rabinovich, E., Patel, R. N., Mirkin, S., Specia, L., and Wintner, S. (2017). Personalized machine translation: Preserving original author traits. Proceedings of the 15th Conference of the European Chapter of the Association for Computational Linguistics: Volume 1, Long Papers. Valencia, Spain (1074–1084). 10.18653/v1/E17-1101.

[S5] Bawden, R. (2017). Machine translation of speech-like texts: Strategies for the inclusion of context. Actes des 24ème Conférence sur le Traitement Automatique des Langues Naturelles. 19es REncontres jeunes Chercheurs en Informatique pour le TAL (RECITAL 2017). (1–14).

[S6] Popel, M. (2018). CUNI transformer neural MT system for WMT18. Proceedings of the Third Conference on Machine Translation: Shared Task Papers. (482–487). 10.18653/v1/W18-6424.

[S7] Vanmassenhove, E., Hardmeier, C., and Way, A. (2018). Getting gender right in neural machine translation. Proceedings of the 2018 Conference on Empirical Methods in Natural Language Processing. (3003–3008). 10.18653/v1/D18-1334.

[S8] Michel, P., and Neubig, G. (2018). Extreme adaptation for personalized neural machine translation. Proceedings of the 56th Annual Meeting of the Association for Computational Linguistics (Volume 2: Short Papers). (312–318). 10.18653/v1/P18-2050.

[S9] Moryossef, A., Aharoni, R., and Goldberg, Y. (2019). Filling gender & number gaps in neural machine translation with black-box context injection. Proceedings of the First Workshop on Gender Bias in Natural Language Processing. (49–54). 10.18653/v1/W19-3807.

[S10] Escudé Font, J., and Costa-jussà, M. R. (2019). Equalizing gender bias in neural machine translation with word embeddings techniques. Proceedings of the First Workshop on Gender Bias in Natural Language Processing. (147–154). 10.18653/v1/W19-3821.

[S11] Cho, W. I., Kim, J. W., Kim, S. M., and Kim, N. S. (2019). On measuring gender bias in translation of gender-neutral pronouns. Proceedings of the First Workshop on Gender Bias in Natural Language Processing. (173–181). 10.18653/v1/W19-3824.

[S12] Stanovsky, G., Smith, N. A., and Zettlemoyer, L. (2019). Evaluating gender bias in machine translation. Proceedings of the 57th Annual Meeting of the Association for Computational Linguistics. (1679–1684). 10.18653/v1/P19-1164.

[S13] Habash, N., Bouamor, H., and Chung, C. (2019). Automatic gender identification and reinflection in Arabic. Proceedings of the First Workshop on Gender Bias in Natural Language Processing. (155–165). 10.18653/v1/W19-3822.

[S14] Stafanovičs, A., Bergmanis, T., and Pinnis, M. (2020). Mitigating gender bias in machine translation with target gender annotations. Proceedings of the Fifth Conference on Machine Translation. (629–638).

[S15] Basta, C., Costa-jussà, M. R., and Fonollosa, J. A. R. (2020). Towards mitigating gender bias in a decoder-based neural machine translation model by adding contextual information. Proceedings of the The Fourth Widening Natural Language Processing Workshop. (99–102). 10.18653/v1/2020.winlp-1.25.

[S16] Costa-jussà, M. R., and de Jorge, A. (2020). Fine-tuning neural machine translation on gender-balanced datasets. Proceedings of the Second Workshop on Gender Bias in Natural Language Processing. (26–34).

[S17] Saunders, D., Sallis, R., and Byrne, B. (2020). Neural machine translation doesn't translate gender coreference right unless you make it. Proceedings of the Second Workshop on Gender Bias in Natural Language Processing. (35–43).

[S18] Gonen, H., and Webster, K. (2020). Automatically identifying gender issues in machine translation using perturbations. Findings of the Association for Computational Linguistics: EMNLP 2020. (1991–1995). 10.18653/v1/2020.findings-emnlp.180.

[S19] Gaido, M., Savoldi, B., Bentivogli, L., Negri, M., and Turchi, M. (2020). Breeding gender-aware direct speech translation systems. Proceedings of the 28th International Conference on Computational Linguistics. (3951–3964). 10.18653/v1/2020.coling-main.350.

[S20] Stojanovski, D., Krojer, B., Peskov, D., and Fraser, A. (2020). ContraCAT: Contrastive coreference analytical templates for machine translation. Proceedings of the 28th International Conference on Computational Linguistics. (4732–4749). 10.18653/v1/2020.coling-main.417.

[S21] Rescigno, A. A., Monti, J., Way, A., and Vanmassenhove, E. (2020). A case study of natural gender phenomena in translation: A comparison of Google Translate, bing Microsoft translator and DeepL for English to Italian, French and Spanish. Workshop on the Impact of Machine Translation (iMpacT 2020). (62–90).

[S22] Bentivogli, L., Savoldi, B., Negri, M., Di Gangi, M. A., Cattoni, R., and Turchi, M. (2020). Gender in danger? evaluating speech translation technology on the MuST-SHE corpus. Proceedings of the 58th Annual Meeting of the Association for Computational Linguistics. (6923–6933). 10.18653/v1/2020.acl-main.619.

[S23] Saunders, D., and Byrne, B. (2020). Reducing gender bias in neural machine translation as a domain adaptation problem. Proceedings of the 58th Annual Meeting of the Association for Computational Linguistics. (7724–7736). 10.18653/v1/2020.acl-main.690.

[S24] Hovy, D., Bianchi, F., and Fornaciari, T. (2020). "you sound just like your father" commercial machine translation systems include stylistic biases. Proceedings of the 58th Annual Meeting of the Association for Computational Linguistics. (1686–1690). 10.18653/v1/2020.acl-main.154.

[S25] González, A. V., Barrett, M., Hvingelby, R., Webster, K., and Søgaard, A. (2020). Type B reflexivization as an unambiguous testbed for multilingual multi-task gender bias. Proceedings of the 2020 Conference on Empirical Methods in Natural Language Processing (EMNLP). (2637–2648). 10.18653/v1/2020.emnlp-main.209.

[S26] Kocmi, T., Limisiewicz, T., and Stanovsky, G. (2020). Gender coreference and bias evaluation at WMT 2020. Proceedings of the Fifth Conference on Machine Translation. (357–364).

[S27] Costa-jussà, M. R., Li Lin, P., and España-Bonet, C. (2020). GeBioToolkit: Automatic extraction of gender-balanced multilingual corpus of Wikipedia biographies. Proceedings of the Twelfth Language Resources and Evaluation Conference. (4081–4088).

[S28] Caglayan, O., Ive, J., Haralampieva, V., Madhyastha, P., Barrault, L., and Specia, L. (2020). Simultaneous machine translation with visual context. Proceedings of the 2020 Conference on Empirical Methods in Natural Language Processing (EMNLP). (2350–2361). 10.18653/v1/2020.emnlp-main.184.

[S29] Troles, J.-D., and Schmid, U. (2021). Extending challenge sets to uncover gender bias in machine translation: Impact of stereotypical verbs and adjectives. Proceedings of the Sixth Conference on Machine Translation. (531–541).

[S30] Savoldi, B., Gaido, M., Bentivogli, L., Negri, M., and Turchi, M. (2021). Gender bias in machine translation. Transactions of the Association for Computational Linguistics *9*, 845–874. 10.1162/tacl_a_00401.

[S31] Wisniewski, G., Zhou, L., Ballier, N., and Yvon, F. (2021). Biais de genre dans un système de traduction automatiqueneuronale : une étude préliminaire (gender bias in neural translation : a preliminary study). Actes de la 28e Conférence sur le Traitement Automatique des Langues Naturelles. Volume 1 : conférence principale. (11–25).

[S32] Ciora, C., Iren, N., and Alikhani, M. (2021). Examining covert gender bias: A case study in Turkish and English machine translation models. Proceedings of the 14th International Conference on Natural Language Generation. (55–63).

[S33] Escolano, C., Ojeda, G., Basta, C., and Costa-jussa, M. R. (2021). Multi-task learning for improving gender accuracy in neural machine translation. Proceedings of the 18th International Conference on Natural Language Processing (ICON). (12–17).

[S34] Ramesh, K., Gupta, G., and Singh, S. (2021). Evaluating gender bias in Hindi-English machine translation. Proceedings of the 3rd Workshop on Gender Bias in Natural Language Processing. (16–23). 10.18653/v1/2021.gebnlp-1.3.

[S35] Levy, S., Lazar, K., and Stanovsky, G. (2021). Collecting a large-scale gender bias dataset for coreference resolution and machine translation. Findings of the Association for Computational Linguistics: EMNLP 2021. (2470–2480). 10.18653/v1/2021.findings-emnlp.211.

[S36] Choubey, P. K., Currey, A., Mathur, P., and Dinu, G. (2021). GFST: Gender-filtered self-training for more accurate gender in translation. Proceedings of the 2021 Conference on Empirical Methods in Natural Language Processing. (1640–1654). 10.18653/v1/2021.emnlp-main.123.

[S37] Gaido, M., Savoldi, B., Bentivogli, L., Negri, M., and Turchi, M. (2021). How to split: The effect of word segmentation on gender bias in speech translation. Findings of the Association for Computational Linguistics: ACL-IJCNLP 2021. (3576–3589). 10.18653/v1/2021.findings-acl.313.

[S38] Vanmassenhove, E., Shterionov, D., and Gwilliam, M. (2021). Machine translationese: Effects of algorithmic bias on linguistic complexity in machine translation. Proceedings of the 16th Conference of the European Chapter of the Association for Computational Linguistics: Main Volume. (2203–2213). 10.18653/v1/2021.eacl-main.188.

[S39] Popović, M. (2021). Agree to disagree: Analysis of inter-annotator disagreements in human evaluation of machine translation output. Proceedings of the 25th Conference on Computational Natural Language Learning. (234–243). 10.18653/v1/2021.conll-1.18.

[S40] Vincent, S. (2021). Towards personalised and document-level machine translation of dialogue. Proceedings of the 16th Conference of the European Chapter of the Association for Computational Linguistics: Student Research Workshop. (137–147). 10.18653/v1/2021.eacl-srw.19.

[S41] Renduchintala, A., Diaz, D., Heafield, K., Li, X., and Diab, M. (2021). Gender bias amplification during speed-quality optimization in neural machine translation. Proceedings of the 59th Annual Meeting of the Association for Computational Linguistics and the 11th International Joint Conference on Natural Language Processing (Volume 2: Short Papers). (99–109). 10.18653/v1/2021.acl-short.15.

[S42] Castilho, S., Cavalheiro Camargo, J. L., Menezes, M., and Way, A. (2021). Dela corpus - a document-level corpus annotated with context-related issues. Proceedings of the Sixth Conference on Machine Translation. Online (566–577).

[S43] Wisniewski, G., Zhu, L., Bailler, N., and Yvon, F. (2021). Screening gender transfer in neural machine translation. Proceedings of the Fourth BlackboxNLP Workshop on Analyzing and Interpreting Neural Networks for NLP. (311–321). 10.18653/v1/2021.blackboxnlp-1.24.

[S44] Jain, N., Popović, M., Groves, D., and Vanmassenhove, E. (2021). Generating gender augmented data for NLP. Proceedings of the 3rd Workshop on Gender Bias in Natural Language Processing. (93–102). 10.18653/v1/2021.gebnlp-1.11.

[S45] Vamvas, J., and Sennrich, R. (2021). Contrastive conditioning for assessing disambiguation in MT: A case study of distilled bias. Proceedings of the 2021 Conference on Empirical Methods in Natural Language Processing. (10246–10265). 10.18653/v1/2021.emnlp-main.803.

[S46] Vanmassenhove, E., Emmery, C., and Shterionov, D. (2021). NeuTral Rewriter: A rule-based and neural approach to automatic rewriting into gender neutral alternatives. Proceedings of the 2021 Conference on Empirical Methods in Natural Language Processing. (8940–8948). 10.18653/v1/2021.emnlp-main.704.

[S47] Vanmassenhove, E., and Monti, J. (2021). gENder-IT: An annotated English-Italian parallel challenge set for cross-linguistic natural gender phenomena. Proceedings of the 3rd Workshop on Gender Bias in Natural Language Processing. (1–7). 10.18653/v1/2021.gebnlp-1.1.

[S48] Wisniewski, G., Zhu, L., Ballier, N., and Yvon, F. (2022). Biais de genre dans un système de traduction automatique neuronale : une étude des mécanismes de transfert cross-langue [gender bias in a neural machine translation system: a study of crosslingual transfer mechanisms]. Traitement Automatique des Langues, Volume 63, Numéro 1 : Varia [Varia]. (37–61).

[S49] Costa-jussà, M. R., Basta, C., and Gállego, G. I. (2022). Evaluating gender bias in speech translation. Proceedings of the Thirteenth Language Resources and Evaluation Conference. (2141–2147). 10.18653/v1/2022.lrec-1.230.

[S50] Castilho, S. (2022). How much context span is enough? examining context-related issues for document-level MT. Proceedings of the Thirteenth Language Resources and Evaluation Conference. (3017–3025).

[S51] Gete, H., Etchegoyhen, T., Ponce, D., Labaka, G., Aranberri, N., Corral, A., Saralegi, X., Ellakuria, I., and Martin, M. (2022). Tando: A corpus for document-level machine translation. Proceedings of the Thirteenth Language Resources and Evaluation Conference. (3026–3037).

[S52] Sólmundsdóttir, A., Guðmundsdóttir, D., Stefánsdóttir, L. B., and Ingason, A. (2022). Mean machine translations: On gender bias in Icelandic machine translations. Proceedings of the Thirteenth Language Resources and Evaluation Conference. (3113–3121).

[S53] Savoldi, B., Gaido, M., Bentivogli, L., Negri, M., and Turchi, M. (2022). On the dynamics of gender learning in speech translation. Proceedings of the 4th Workshop on Gender Bias in Natural Language Processing (GeBNLP). (94–111). 10.18653/v1/2022.gebnlp-1.12.

[S54] Měchura, M. (2022). A taxonomy of bias-causing ambiguities in machine translation. Proceedings of the 4th Workshop on Gender Bias in Natural Language Processing (GeBNLP). (168–173). 10.18653/v1/2022.gebnlp-1.18.

[S55] Corral, A., and Saralegi, X. (2022). Gender bias mitigation for NMT involving genderless languages. Proceedings of the Seventh Conference on Machine Translation (WMT). (165–176).

[S56] Mohammadshahi, A., Nikoulina, V., Berard, A., Brun, C., Henderson, J., and Besacier, L. (2022). What do compressed multilingual machine translation models forget? Findings of the Association for Computational Linguistics: EMNLP 2022. (4308–4329). 10.18653/v1/2022.findings-emnlp.317.

[S57] Saunders, D., Sallis, R., and Byrne, B. (2022). First the worst: Finding better gender translations during beam search. Findings of the Association for Computational Linguistics: ACL 2022. (3814–3823). 10.18653/v1/2022.findings-acl.301.

[S58] Currey, A., Nadejde, M., Pappagari, R. R., Mayer, M., Lauly, S., Niu, X., Hsu, B., and Dinu, G. (2022). MT-GenEval: A counterfactual and contextual dataset for evaluating gender accuracy in machine translation. Proceedings of the 2022 Conference on Empirical Methods in Natural Language Processing. (4287–4299). 10.18653/v1/2022.emnlp-main.288.

[S59] Karpinska, M., Raj, N., Thai, K., Song, Y., Gupta, A., and Iyyer, M. (2022). DEMETR: Diagnosing evaluation metrics for translation. Proceedings of the 2022 Conference on Empirical Methods in Natural Language Processing. (9540–9561). 10.18653/v1/2022.emnlp-main.649.

[S60] Zhu, L., Wisniewski, G., Ballier, N., and Yvon, F. (2022). Flux d'informations dans les systèmes encodeur-décodeur. application à l'explication des biais de genre dans les systèmes de traduction automatique. (information flow in encoder-decoder systems applied to the explanation of gender bias in machine translation systems). Actes de la 29e Conférence sur le Traitement Automatique des Langues Naturelles. Atelier TAL et Humanités Numériques (TAL-HN). (10–18).

[S61] Sharma, S., Dey, M., and Sinha, K. (2022). How sensitive are translation systems to extra contexts? mitigating gender bias in neural machine translation models through relevant contexts. Findings of the Association for Computational Linguistics: EMNLP 2022. (1968–1984). 10.18653/v1/2022.findings-emnlp.143.

[S62] Wisniewski, G., Zhu, L., Ballier, N., and Yvon, F. (2022). Analyzing gender translation errors to identify information flows between the encoder and decoder of a NMT system. Proceedings of the Fifth BlackboxNLP Workshop on Analyzing and Interpreting Neural Networks for NLP. (153–163). 10.18653/v1/2022.blackboxnlp-1.13.

[S63] Vincent, S. T., Barrault, L., and Scarton, C. (2022). Controlling extra-textual attributes about dialogue participants: A case study of English-to-Polish neural machine translation. Proceedings of the 23rd Annual Conference of the European Association for Machine Translation. (121–130).

[S64] Daems, J., and Hackenbuchner, J. (2022). DeBiasByUs: Raising awareness and creating a database of MT bias. Proceedings of the 23rd Annual Conference of the European Association for Machine Translation. (289–290).

[S65] Wang, J., Rubinstein, B., and Cohn, T. (2022). Measuring and mitigating name biases in neural machine translation. Proceedings of the 60th Annual Meeting of the Association for Computational Linguistics (Volume 1: Long Papers). (2576–2590). 10.18653/v1/2022.acl-long.184.

[S66] Renduchintala, A., and Williams, A. (2022). Investigating failures of automatic translationin the case of unambiguous gender. Proceedings of the 60th Annual Meeting of the Association for Computational Linguistics (Volume 1: Long Papers). (3454–3469). 10.18653/v1/2022.acl-long.243.

[S67] Wairagala, E. P., Mukiibi, J., Tusubira, J. F., Babirye, C., Nakatumba-Nabende, J., Katumba, A., and Ssenkungu, I. (2022). Gender bias evaluation in Luganda-English machine translation. Proceedings of the 15th biennial conference of the Association for Machine Translation in the Americas (Volume 1: Research Track). (274–286).

[S68] Savoldi, B., Gaido, M., Bentivogli, L., Negri, M., and Turchi, M. (2022). Under the morphosyntactic lens: A multifaceted evaluation of gender bias in speech translation. Proceedings of the 60th Annual Meeting of the Association for Computational Linguistics (Volume 1: Long Papers). (1807–1824). 10.18653/v1/2022.acl-long.127.

[S69] Alrowili, S., and Shanker, V. (2022). Generative approach for gender-rewriting task with arabict5. Proceedings of the Seventh Arabic Natural Language Processing Workshop (WANLP). (491–495). 10.18653/v1/2022.wanlp-1.55.

[S70] Alhafni, B., Habash, N., and Bouamor, H. (2022). User-centric gender rewriting. Proceedings of the 2022 Conference of the North American Chapter of the Association for Computational Linguistics: Human Language Technologies. (618–631). 10.18653/v1/2022.naacl-main.46.

[S71] Alhafni, B., Habash, N., and Bouamor, H. (2022). The Arabic parallel gender corpus 2.0: Extensions and analyses. Proceedings of the Thirteenth Language Resources and Evaluation Conference. (1870–1884).

[S72] Savoldi, B., Gaido, M., Negri, M., and Bentivogli, L. (2023). Test suites task: Evaluation of gender fairness in MT with MuST-SHE and INES. Proceedings of the Eighth Conference on Machine Translation. (252–262). 10.18653/v1/2023.wmt-1.25.

[S73] Gete, H., and Etchegoyhen, T. (2023). An evaluation of source factors in concatenation-based context-aware neural machine translation. Proceedings of the 14th International Conference on Recent Advances in Natural Language Processing. (399–407).

[S74] Dinh, T. A., and Niehues, J. (2023). Perturbation-based QE: An explainable, unsupervised word-level quality estimation method for blackbox machine translation. Proceedings of Machine Translation Summit XIX, Vol. 1: Research Track. (59–71).

[S75] Singh, P. (2023). Gender inflected or bias inflicted: On using grammatical gender cues for bias evaluation in machine translation. Proceedings of the 13th International Joint Conference on Natural Language Processing and the 3rd Conference of the Asia-Pacific Chapter of the Association for Computational Linguistics: Student Research Workshop. (17–23). 10.18653/v1/2023.ijcnlp-srw.3.

[S76] Iluz, B., Limisiewicz, T., Stanovsky, G., and Mareček, D. (2023). Exploring the impact of training data distribution and subword tokenization on gender bias in machine translation. Proceedings of the 13th International Joint Conference on Natural Language Processing and the 3rd Conference of the Asia-Pacific Chapter of the Association for Computational Linguistics (Volume 1: Long Papers). (885–896). 10.18653/v1/2023.ijcnlp-main.57.

[S77] Alhafni, B., Obeid, O., and Habash, N. (2023). The user-aware Arabic gender rewriter. Proceedings of the First Workshop on Gender-Inclusive Translation Technologies. (3–11).

[S78] Sandoval, S., Zhao, J., Carpuat, M., and Daumé III, H. (2023). A rose by any other name would not smell as sweet: Social bias in names mistranslation. Proceedings of the 2023 Conference on Empirical Methods in Natural Language Processing. (3933–3945). 10.18653/v1/2023.emnlp-main.239.

[S79] Wicks, R., and Post, M. (2023). Identifying context-dependent translations for evaluation set production. Proceedings of the Eighth Conference on Machine Translation. (452–467). 10.18653/v1/2023.wmt-1.42.

[S80] Triboulet, B., and Bouillon, P. (2023). Evaluating the impact of stereotypes and language combinations on gender bias occurrence in NMT generic systems. Proceedings of the Third Workshop on Language Technology for Equality, Diversity and Inclusion. (62–70).

[S81] Gromann, D., Lardelli, M., Spiel, K., Burtscher, S., Klausner, L. D., Mettinger, A., Miladinovic, I., Schefer-Wenzl, S., Duh, D., and Bühn, K. (2023). Participatory research as a path to community-informed, gender-fair machine translation. Proceedings of the First Workshop on Gender-Inclusive Translation Technologies. (49–59).

[S82] Paolucci, A. B., Lardelli, M., and Gromann, D. (2023). Gender-fair language in translation: A case study. Proceedings of the First Workshop on Gender-Inclusive Translation Technologies. (13–23).

[S83] Lardelli, M., and Gromann, D. (2023). Gender-fair post-editing: A case study beyond the binary. Proceedings of the 24th Annual Conference of the European Association for Machine Translation. (251–260).

[S84] Piergentili, A., Fucci, D., Savoldi, B., Bentivogli, L., and Negri, M. (2023). Gender neutralization for an inclusive machine translation: from theoretical foundations to open challenges. Proceedings of the First Workshop on Gender-Inclusive Translation Technologies. (71–83).

[S85] Saunders, D., and Olsen, K. (2023). Gender, names and other mysteries: Towards the ambiguous for gender-inclusive translation. Proceedings of the First Workshop on Gender-Inclusive Translation Technologies. (85–93).

[S86] Kostikova, A., Daems, J., and Lazarov, T. (2023). How adaptive is adaptive machine translation, really? a gender-neutral language use case. Proceedings of the First Workshop on Gender-Inclusive Translation Technologies. (95–97).

[S87] Costa-jussà, M., Smith, E., Ropers, C., Licht, D., Maillard, J., Ferrando, J., and Escolano, C. (2023). Toxicity in multilingual machine translation at scale. Findings of the Association for Computational Linguistics: EMNLP 2023. (9570–9586). 10.18653/v1/2023.findings-emnlp.642.

[S88] Costa-jussà, M., Andrews, P., Smith, E., Hansanti, P., Ropers, C., Kalbassi, E., Gao, C., Licht, D., and Wood, C. (2023). Multilingual holistic bias: Extending descriptors and patterns to unveil demographic biases in languages at scale. Proceedings of the 2023 Conference on Empirical Methods in Natural Language Processing. (14141–14156). 10.18653/v1/2023.emnlp-main.874.

[S89] Cabrera, L., and Niehues, J. (2023). Gender lost in translation: How bridging the gap between languages affects gender bias in zero-shot multilingual translation. Proceedings of the First Workshop on Gender-Inclusive Translation Technologies. (25–35).

[S90] Daems, J. (2023). Gender-inclusive translation for a gender-inclusive sport: strategies and translator perceptions at the international quadball association. Proceedings of the First Workshop on Gender-Inclusive Translation Technologies. (37–47).

[S91] Fucci, D., Gaido, M., Papi, S., Cettolo, M., Negri, M., and Bentivogli, L. (2023). Integrating language models into direct speech translation: An inference-time solution to control gender inflection. Proceedings of the 2023 Conference on Empirical Methods in Natural Language Processing. Singapore (11505–11517). 10.18653/v1/2023.emnlp-main.705.

[S92] Piergentili, A., Savoldi, B., Fucci, D., Negri, M., and Bentivogli, L. (2023). Hi guys or hi folks? benchmarking gender-neutral machine translation with the GeNTE corpus. Proceedings of the 2023 Conference on Empirical Methods in Natural Language Processing. (14124–14140). 10.18653/v1/2023.emnlp-main.873.

[S93] Lu, T., Aepli, N., and Rios, A. (2023). Reducing gender bias in NMT with FUDGE. Proceedings of the First Workshop on Gender-Inclusive Translation Technologies. (61–69).

[S94] Castilho, S., Mallon, C. Q., Meister, R., and Yue, S. (2023). Do online machine translation systems care for context? what about a gpt model? Proceedings of the 24th Annual Conference of the European Association for Machine Translation. (393–417).

[S95] Paulo, M., Cabarrão, V., Moniz, H., Menezes, M., Grewcock, R., and Farah, E. (2023). Context-aware and gender-neutral translation memories. Proceedings of the 24th Annual Conference of the European Association for Machine Translation. (437–444).

[S96] Le, N. T., Hansal, O., and Sadat, F. (2023). Challenges and issue of gender bias in under-represented languages: An empirical study on Inuktitut-English NMT. Proceedings of the Sixth Workshop on the Use of Computational Methods in the Study of Endangered Languages. (89–97).

[S97] Sarti, G., Htut, P. M., Niu, X., Hsu, B., Currey, A., Dinu, G., and Nadejde, M. (2023). RAMP: Retrieval and attribute-marking enhanced prompting for attribute-controlled translation. Proceedings of the 61st Annual Meeting of the Association for Computational Linguistics (Volume 2: Short Papers). (1476–1490). 10.18653/v1/2023.acl-short.126.

[S98] Lauscher, A., Nozza, D., Miltersen, E., Crowley, A., and Hovy, D. (2023). What about “em”? how commercial machine translation fails to handle (neo-)pronouns. Proceedings of the 61st Annual Meeting of the Association for Computational Linguistics (Volume 1: Long Papers). (377–392). 10.18653/v1/2023.acl-long.23.

[S99] Vincent, S., Flynn, R., and Scarton, C. (2023). Mtcue: Learning zero-shot control of extra-textual attributes by leveraging unstructured context in neural machine translation. Findings of the Association for Computational Linguistics: ACL 2023. Toronto, Canada (8210–8226). 10.18653/v1/2023.findings-acl.521.

[S100] Attanasio, G., Plaza del Arco, F. M., Nozza, D., and Lauscher, A. (2023). A tale of pronouns: Interpretability informs gender bias mitigation for fairer instruction-tuned machine translation. Proceedings of the 2023 Conference on Empirical Methods in Natural Language Processing. (3996–4014). 10.18653/v1/2023.emnlp-main.243.

[S101] Lee, M., Koh, H., Lee, K.-i., Zhang, D., Kim, M., and Jung, K. (2023). Target-agnostic gender-aware contrastive learning for mitigating bias in multilingual machine translation. Proceedings of the 2023 Conference on Empirical Methods in Natural Language Processing. (16825–16839). 10.18653/v1/2023.emnlp-main.1046.

[S102] Wang, L., Liu, S., Xu, M., Song, L., Shi, S., and Tu, Z. (2023). A survey on zero pronoun translation. Proceedings of the 61st Annual Meeting of the Association for Computational Linguistics (Volume 1: Long Papers). (3325–3339). 10.18653/v1/2023.acl-long.187.

[S103] Veloso, L., Coheur, L., and Ribeiro, R. (2023). A rewriting approach for gender inclusivity in Portuguese. Findings of the Association for Computational Linguistics: EMNLP 2023. (8747–8759). 10.18653/v1/2023.findings-emnlp.585.

[S104] Amrhein, C., Schottmann, F., Sennrich, R., and Läubli, S. (2023). Exploiting biased models to de-bias text: A gender-fair rewriting model. Proceedings of the 61st Annual Meeting of the Association for Computational Linguistics (Volume 1: Long Papers). (4486–4506). 10.18653/v1/2023.acl-long.246.

[S105] Soler Uguet, C., Bane, F., Aymo, M., Fernandes Torres, J. P., Zaretskaya, A., and Blanch Miró, T. (2023). Enhancing gender representation in neural machine translation: A comparative analysis of annotating strategies for English-Spanish and English-Polish language pairs. Proceedings of Machine Translation Summit XIX, Vol. 2: Users Track. (171–172).

[S106] Sarti, G., Feldhus, N., Sickert, L., and van der Wal, O. (2023). Inseq: An interpretability toolkit for sequence generation models. Proceedings of the 61st Annual Meeting of the Association for Computational Linguistics (Volume 3: System Demonstrations). (421–435). 10.18653/v1/2023.acl-demo.40.

[S107] Savoldi, B., Piergentili, A., Fucci, D., Negri, M., and Bentivogli, L. (2024). A prompt response to the demand for automatic gender-neutral translation. Proceedings of the 18th Conference of the European Chapter of the Association for Computational Linguistics (Volume 2: Short Papers). (256–267).

[S108] Liu, D., and Niehues, J. (2024). How transferable are attribute controllers on pretrained multilingual translation models? Proceedings of the 18th Conference of the European Chapter of the Association for Computational Linguistics (Volume 1: Long Papers). (334–348).

[S109] Mash, A., Escolano, C., Sant, A., Melero, M., and de Luca Fornaciari, F. (2024). Unmasking biases: Exploring gender bias in English-Catalan machine translation through tokenization analysis and novel dataset. Proceedings of the 2024 Joint International Conference on Computational Linguistics, Language Resources and Evaluation (LREC-COLING 2024). (17144–17153).

[S110] Lee, M., Koh, H., Kim, M., and Jung, K. (2024). Fine-grained gender control in machine translation with large language models. Proceedings of the 2024 Conference of the North American Chapter of the Association for Computational Linguistics: Human Language Technologies (Volume 1: Long Papers). (5416–5430).

[S111] Costa-jussà, M., Andrews, P., Basta, C., Ciro, J., Falenska, A., Goldfarb-Tarrant, S., Mosquera, R., Nozza, D., and Sánchez, E. (2024). Overview of the shared task on machine translation gender bias evaluation with multilingual holistic bias. Proceedings of the 5th Workshop on Gender Bias in Natural Language Processing (GeBNLP). (399–404). `10.18653/v1/2024.gebnlp-1.26`.

[S112] Daems, J. (2024). Pilot testing gender-inclusive translations and machine translations for German quadball referee certification test takers. Proceedings of the 2nd International Workshop on Gender-Inclusive Translation Technologies. (56–57).

[S113] Friðriksdóttir, S. R. (2024). The GenderQueer test suite. Proceedings of the Ninth Conference on Machine Translation. (327–340). `10.18653/v1/2024.wmt-1.26`.

[S114] Garg, S., Gheini, M., Emmanuel, C., Likhomanenko, T., Gao, Q., and Paulik, M. (2024). Generating gender alternatives in machine translation. Proceedings of the 5th Workshop on Gender Bias in Natural Language Processing (GeBNLP). (237–254). `10.18653/v1/2024.gebnlp-1.15`.

[S115] Gete, H., and Etchegoyhen, T. (2024). Does context help mitigate gender bias in neural machine translation? Findings of the Association for Computational Linguistics: EMNLP 2024. (14788–14794). `10.18653/v1/2024.findings-emnlp.868`.

[S116] Hackenbuchner, J., Daems, J., Tezcan, A., and Maladry, A. (2024). You shall know a word's gender by the company it keeps: Comparing the role of context in human gender assumptions with MT. Proceedings of the 2nd International Workshop on Gender-Inclusive Translation Technologies. (31–41).

[S117] Iluz, B., Elazar, Y., Yehudai, A., and Stanovsky, G. (2024). Applying intrinsic debiasing on downstream tasks: Challenges and considerations for machine translation. Proceedings of the 2024 Conference on Empirical Methods in Natural Language Processing. (14914–14921). `10.18653/v1/2024.emnlp-main.829`.

[S118] Lardelli, M., Attanasio, G., and Lauscher, A. (2024). Building bridges: A dataset for evaluating gender-fair machine translation into German. Findings of the Association for Computational Linguistics: ACL 2024. (7542–7550). `10.18653/v1/2024.findings-acl.448`.

[S119] Lardelli, M., Dill, T., Attanasio, G., and Lauscher, A. (2024). Sparks of fairness: Preliminary evidence of commercial machine translation as English-to-German gender-fair dictionaries. Proceedings of the 2nd International Workshop on Gender-Inclusive Translation Technologies. (12–21).

[S120] Luthra, M., and Nijman, B. (2024). Lost in translation? approaches to gender representation in multilingual archives. Proceedings of the 2nd International Workshop on Gender-Inclusive Translation Technologies. (42–55).

[S121] Nunziatini, M., and Diego, S. (2024). Implementing gender-inclusivity in mt output using automatic post-editing with llms. Proceedings of the 25th Annual Conference of the European Association for Machine Translation (Volume 1). (580–589).

[S122] Piergentili, A., Savoldi, B., Negri, M., and Bentivogli, L. (2024). Enhancing gender-inclusive machine translation with neomorphemes and large language models. Proceedings of the 25th Annual Conference of the European Association for Machine Translation (Volume 1). (300–314).

[S123] Pikuliak, M., Oresko, S., Hrckova, A., and Simko, M. (2024). Women are beautiful, men are leaders: Gender stereotypes in machine translation and language modeling. Findings of the Association for Computational Linguistics: EMNLP 2024. (3060–3083). 10.18653/v1/2024.findings-emnlp.173.

[S124] Popovic, M., and Lapshinova-Koltunski, E. (2024). Gender and bias in Amazon review translations: by humans, MT systems and ChatGPT. Proceedings of the 2nd International Workshop on Gender-Inclusive Translation Technologies. (22–30).

[S125] Rarrick, S., Naik, R., Poudel, S., and Chowdhary, V. (2024). GATE X-E : A challenge set for gender-fair translations from weakly-gendered languages. Findings of the Association for Computational Linguistics: ACL 2024. (8526–8546). 10.18653/v1/2024.findings-acl.504.

[S126] Robinson, K., Kudugunta, S., Stella, R., Dev, S., and Bastings, J. (2024). MiTTenS: A dataset for evaluating gender mistranslation. Proceedings of the 2024 Conference on Empirical Methods in Natural Language Processing. (4115–4124). 10.18653/v1/2024.emnlp-main.238.

[S127] Sánchez, E., Andrews, P., Stenetorp, P., Artetxe, M., and Costa-jussà, M. R. (2024). Gender-specific machine translation with large language models. Proceedings of the Fourth Workshop on Multilingual Representation Learning (MRL 2024). (148–158). 10.18653/v1/2024.mrl-1.10.

[S128] Sant, A., Escolano, C., Mash, A., De Luca Fornaciari, F., and Melero, M. (2024). The power of prompts: Evaluating and mitigating gender bias in MT with LLMs. Proceedings of the 5th Workshop on Gender Bias in Natural Language Processing (GeBNLP). (94–139). 10.18653/v1/2024.gebnlp-1.7.

[S129] Savoldi, B., Gaido, M., Negri, M., and Bentivogli, L. (2024). FBK@IWSLT test suites task: Gender bias evaluation with MuST-SHE. Proceedings of the 21st International Conference on Spoken Language Translation (IWSLT 2024). (65–71). 10.18653/v1/2024.iwslt-1.10.

[S130] Savoldi, B., Papi, S., Negri, M., Guerberof-Arenas, A., and Bentivogli, L. (2024). What the harm? quantifying the tangible impact of gender bias in machine translation with a human-centered study. Proceedings of the 2024 Conference on Empirical Methods in Natural Language Processing. (18048–18076). 10.18653/v1/2024.emnlp-main.1002.

[S131] Sewunetie, W., Tonja, A., Belay, T., Nigatu, H. H., Gebremeskel, G., Mossie, Z., Seid, H., and Yimam, S. (2024). Gender bias evaluation in machine translation for Amharic, Tigrigna, and afaan oromoo. Proceedings of the 2nd International Workshop on Gender-Inclusive Translation Technologies. (1–11).

[S132] Stewart, I., and Mihalcea, R. (2024). Whose wife is it anyway? assessing bias against same-gender relationships in machine translation. Proceedings of the 5th Workshop on Gender Bias in Natural Language Processing (GeBNLP). (365–375). 10.18653/v1/2024.gebnlp-1.23.

[S133] Üstün, A., Aryabumi, V., Yong, Z., Ko, W.-Y., D'souza, D., Onilude, G., Bhandari, N., Singh, S., Ooi, H.-L., Kayid, A., Vargus, F., Blunsom, P., Longpre, S., Muennighoff, N., Fadaee, M., Kreutzer, J., and Hooker, S. (2024). Aya model: An instruction finetuned open-access multilingual language model. Proceedings of the 62nd Annual Meeting of the Association for Computational Linguistics (Volume 1: Long Papers). (15894–15939). 10.18653/v1/2024.acl-long.845.

[S134] Zerva, C., Blain, F., C. De Souza, J. G., Kanojia, D., Deoghare, S., Guerreiro, N. M., Attanasio, G., Rei, R., Orasan, C., Negri, M., Turchi, M., Chatterjee, R., Bhattacharyya, P., Freitag, M., and Martins, A. (2024). Findings of the quality estimation shared task at WMT 2024: Are LLMs closing the gap in QE? Proceedings of the Ninth Conference on Machine Translation. (82–109). 10.18653/v1/2024.wmt-1.3.

[S135] Adelani, D., Zhang, M., Shen, X., Davody, A., Kleinbauer, T., and Klakow, D. (2021). Preventing author profiling through zero-shot multilingual back-translation. Proceedings of the 2021 Conference on Empirical Methods in Natural Language Processing. (8687–8695). 10.18653/v1/2021.emnlp-main.684.
